# Transcript profiles of wild and domesticated sorghum under water-stressed conditions and the differential impact on dhurrin metabolism

**DOI:** 10.1007/s00425-022-03831-4

**Published:** 2022-01-27

**Authors:** Galaihalage K. S. Ananda, Sally L. Norton, Cecilia Blomstedt, Agnelo Furtado, Birger Lindberg Møller, Roslyn Gleadow, Robert J. Henry

**Affiliations:** 1 Queensland Alliance for Agriculture and Food Innovation, The University of Queensland, St Lucia, QLD Australia; 2 Australian Grains Genebank, Agriculture Victoria, Horsham, VIC Australia; 3 School of Biological Sciences, Monash University, Clayton, VIC Australia; 4 Plant Biochemistry Laboratory, Department of Plant and Environmental Sciences, University of Copenhagen, Copenhagen, Denmark

**Keywords:** Cyanogenesis, Dhurrin metabolism, Gene expression, Sorghum, Water-stress, Wild sorghum

## Abstract

**Main conclusion:**

**Australian native species of sorghum contain negligible amounts of dhurrin in their leaves and the cyanogenesis process is regulated differently under water-stress in comparison to domesticated sorghum species.**

**Abstract:**

Cyanogenesis in forage sorghum is a major concern in agriculture as the leaves of domesticated sorghum are potentially toxic to livestock, especially at times of drought which induces increased production of the cyanogenic glucoside dhurrin. The wild sorghum species endemic to Australia have a negligible content of dhurrin in the above ground tissues and thus represent a potential resource for key agricultural traits like low toxicity. In this study we investigated the differential expression of cyanogenesis related genes in the leaf tissue of the domesticated species *Sorghum bicolor* and the Australian native wild species *Sorghum macrospermum* grown in glasshouse-controlled water-stress conditions using RNA-Seq analysis to analyse gene expression. The study identified genes, including those in the cyanogenesis pathway, that were differentially regulated in response to water-stress in domesticated and wild sorghum. In the domesticated sorghum, dhurrin content was significantly higher compared to that in the wild sorghum and increased with stress and decreased with age whereas in wild sorghum the dhurrin content remained negligible. The key genes in dhurrin biosynthesis, *CYP79A1*, *CYP71E1* and *UGT85B1*, were shown to be highly expressed in *S. bicolor*. *DHR* and *HNL* encoding the dhurrinase and α-hydroxynitrilase catalysing bio-activation of dhurrin were also highly expressed in *S. bicolor*. Analysis of the differences in expression of cyanogenesis related genes between domesticated and wild sorghum species may allow the use of these genetic resources to produce more acyanogenic varieties in the future.

**Supplementary Information:**

The online version contains supplementary material available at 10.1007/s00425-022-03831-4.

## Introduction

Water-stress is a key abiotic stress factor in crop production causing reduced yields (Farooq et al. 2009) and inducing vastly altered expression profiles for genes related to plant growth and development (Takahashi et al. 2018; Luo et al. 2014; Shankar et al. 2013; Ahuja et al. 2010). Water-stress tolerance in sorghum is regulated by the combined effects of genotype and environment (Borrell et al. 2006). Domesticated sorghum, *Sorghum bicolor* (L.) Moench is tolerant to water-stress but is susceptible to severe droughts (Borrell et al. 2014). RNA sequencing technologies have been used in sorghum to analyse drought tolerance (Dugas et al. 2011), fungal resistance (Mizuno et al. 2012) and the role of miRNAs (Calviño et al. 2011). The differential gene expression of *S. bicolor* in response to water-stress has been investigated in several studies (Abdel-Ghany et al. 2020; Johnson et al. 2014; Dugas et al. 2011) and has identified many up-regulated and down-regulated genes encoding formation of protective molecules and regulatory factors such as dehydrins (DHN), aquaporins (AQP), abscisic acid (ABA) responsive proteins, drought-responsive element binding proteins (DREB), cryoprotectants, detoxification related genes, osmoprotectants as well as membrane composition and signalling molecules (Abdel-Ghany et al. 2020; Azzouz-Olden et al. 2020; Gosal et al. 2009; Fetter et al. 2004).

In sorghum, water-stress induces the accumulation of the cyanogenic glucoside, dhurrin ((*S*)-4-hydroxymandelonitrile-β-d-glucopyranoside) (Gleadow et al. 2016; Neilson et al. 2015; Gleadow and Moller 2014; O’Donnell et al. 2013). Cyanogenic glycosides are amino acid derived bioactive compounds identified in more than 3000 plant species (Gleadow and Moller 2014). In addition to sorghum (Kojima et al. 1979), species such as almonds (Sánchez-Pérez et al. 2019), cassava (Jorgensen et al. 2005), macadamia nuts (Dahler et al. 1995) and barley (Knoch et al. 2016) produce cyanogenic glycosides.

Dhurrin is present in all major tissues of domesticated sorghum species except the mature grain (Nielsen et al. 2016a; Kahn et al. 1997). Dhurrin is part of a two-component defence system activated upon cell destruction e.g., as caused by a chewing herbivore. The bio-activation process is termed cyanogenesis and results in detonation of a hydrogen cyanide (HCN) bomb and concomitant production of stoichiometric amounts of *p-*hydroxybenzaldehyde (Gleadow and Moller 2014; Møller 2010) and is catalysed by specific β-glucosidases (dhurrinases, DHR1 and DHR2) and an α-hydroxynitrilase (HNL). HCN inhibits metalloenzymes in cytochrome *c* oxidase and may disrupt the mitochondrial respiratory electron transport chain (Nielsen et al. 2016a) and when ingested at sufficiently high concentrations be lethal in humans (Loyd and Gray 1970) and animals (Finnie et al. 2011). Cyanogenic glucosides may therefore serve as effective agents against generalist herbivores (Ballhorn et al. 2008; Zagrobelny et al. 2004; Gleadow and Woodrow 2002b). The dhurrin content in forage sorghum may be high and upon bio-activation generate a HCN content exceeding the 600-ppm maximum content for safe grazing by cattle (Gleadow et al. 2016). Humans may inadvertently have selected for cyanogenic plants over non-cyanogenic plants during evolution (Cowan et al. 2020; Jones 1998) either because their improved resistance to herbivores (McKey et al. 2010; Jones 1998) or better nitorgen use efficiency (Myrans et al. 2020; Rosati et al. 2019b). Dhurrin levels decrease with tissue age in sorghum and the highest biosynthesis and accumulation rate is observed in young sorghum seedlings (Busk and Møller 2002; Gleadow and Woodrow 2002b).

The biosynthetic pathway of CNglcs has been extensively studied in *S. bicolor* (Møller and Conn 1979) and the pathway intermediates and enzymes have been identified (Jones et al. 1999; Bak et al. 1998; Kahn et al. 1997; Sibbesen et al. 1994). In the first step, tyrosine is converted to (*E*)-*p*-hydroxyphenyl acetaldoxime by CYP79A1 (Sibbesen et al. 1995). Then CYP71E1 catalyses the conversion of the oxime into the cyanohydrin *p*-hydroxymandelonitrile (Bak et al. 1998) which upon glycosylation, catalysed by UGT85B1, is converted into dhurrin (Jones et al. 1999). In these processes, the NADPH-dependent cytochrome P450 oxidoreductase (POR) serves as an obligatory electron donor to the two P450s (Jensen et al. 2021; Halkier and Møller 1991) and is localized in the endoplasmic reticulum forming an enzyme complex (metabolon) with CYP79A1 and CYP71E1 that recruits the UGT85B1 (Laursen et al. 2015; Jensen et al. 2011; Nielsen et al. 2008). The formation of a metabolon prevents the escape of toxic intermediates. In sorghum, uptake of excess nitrogen is primarily stored as nitrate but a trade-off mechanism in partitioning nitrogen between dhurrin and nitrate may operate (Gleadow et al. 2016).

In addition to its role in defence against herbivores, dhurrin also serves as a storage form of reduced nitrogen (Bjarnholt et al. 2018; Nielsen et al. 2016b; Pičmanová et al. 2015; Jenrich et al. 2007). The recycle pathway proceeds without the release of HCN and is catalysed by a glutathione transferase (GSTL1 or GSTL2) and a heteromer of the nitrilases, NIT4A and NIT4B2 (Bjarnholt et al. 2018). In a detoxification reaction catalysed by β-cyanoalanine synthase (CAS) and using cysteine as substrate, HCN released by the bio-activation reaction may be incorporated into β-cyanoalanine (Piotrowski and Volmer 2006) and further converted to asparagine, aspartate and ammonia by heteromers of nitrilases (NIT4) of the A and B types (Jenrich et al. 2007).

Seventeen wild sorghum species endemic to Australia (Ananda et al. 2020) are known and vary in their resistance to water-stress (Myrans et al. 2020). The effects of severe water-stress conditions on the growth, morphology, physiological and biological characteristics of wild sorghum species from different subgenera have recently been reported (Cowan et al. 2020). Whereas water-stress significantly increases the dhurrin levels in the above ground tissue (leaves and sheath) of *S. bicolor,* dhurrin levels remained unchanged in the wild relatives, except for a significant decrease in the sheath tissue in some wild species. Water-stress caused a significant growth reduction in *S. bicolor* whereas the wild species were more tolerant. The wild sorghum species, *S. macrospermum* E. D. Garber maintained a high relative growth rate and an insignificant aboveground dhurrin content under water-stress. The dhurrin levels in the leaves of *S. macrospermum* was 1000-fold lower than in the leaves of *S. bicolor,* while water-stress did not significantly increase the leaf dhurrin levels of *S. macrospermum*. Another recent study (Cowan et al. 2022) supports these findings showing that the leaf dhurrin content of wild sorghum species are significantly lower than those in *S. bicolor*. However, the dhurrin levels in the roots of wild sorghum species are similar to the levels in the domesticated *S. bicolor* (Cowan et al. 2021). The observed differences in dhurrin regulation between domesticated and wild species may mirror the differences in selective pressures encountered in natural and cultivated habitats (Bredeson et al. 2016). The maintained dhurrin content in the roots of wild sorghum may represent a recyclable reduced nitrogen store facilitating growth in the nutrient poor environments, characteristic of the native ranges of the wild species (Cowan et al. 2020; Myrans et al. 2020; Pičmanová et al. 2015; Dillon et al. 2007).

Crop wild relatives are expected to harbour valuable genetic traits that can be used in crop improvement (Ananda et al. 2020). A major constraint in utilising the genetic resources in wild crop relatives is hindrance in gene transfer between domesticated crops and their wild relatives (Bevan et al. 2017). However, the Australian endemic wild sorghum species, *S. macrospermum* is closely related to domesticated *S. bicolor* (Ananda et al. 2021), with successful introgression of the two species reported by Kuhlman et al. (2010). The regulation of the synthesis of dhurrin in wild sorghum species in comparison to domesticated *S. bicolor* has not been studied. This study provides new knowledge on the differences in gene expression profiles in wild and domesticated sorghum including dhurrin biosynthesis, bio-activation and recycling.

## Materials and methods

### Plant material, sample collection and processing

The domesticated species of sorghum *S. bicolor* (L.) Moench (AGG 314746) and one wild species *S. macrospermum* E. D. Garber (AGG 302367) were selected based on the results of Cowan et al. (2020). Seeds of each accession were obtained from the Australian Grains Genebank (AGG), Horsham, Victoria and germinated according to the reported optimised protocol (Cowan et al. 2020). The two genotypes were grown at the glasshouse complex at the University of Queensland (UQ), QLD (27.4975° S, 153.0137° E) using a complete randomised design. The experiment was conducted in July–October 2020. Seeds were planted in 4 L ANOVA pots in a UQ23 soil mix (250 L) (70% Composite Pine Bark 0–5 mm, 30% Coco Peat and Fertilizers and other augments/M^3^ (1 kg Yates Flowtrace, 1 kg iron sulphate heptahydrate, 0.4 kg superphosphate [Ca (H_2_PO_4_)_2_], 0.03 kg copper sulphate, 1 kg gypsum, 1 kg dolomite, 6 g Osmocte L^−1^)), with five replicates for each well-watered condition (control) and water-stressed (stress) conditions. At the latter conditions, only four replicates of *S. macrospermum* survived to the very end of the experiment. The glasshouse was maintained at 28 °C and 18 °C ± 1.5 °C day/night with an average photoperiod of 14 h and with an average light intensity of 1100 µmol quanta m^−2^ s^−1^. The growth room was equipped with four Heliospectra LX602-G growth lights (blue 450 nm, white 5700 K, red 660 nm). For *S. bicolor,* all plants were watered daily to 100% soil water capacity for the first 10 d. After 10 d, the well-watered (control) plants were maintained at 100% soil water capacity, whilst the water-stressed (stress) plants were maintained at a soil water capacity of 15% obtained after decreasing the water content gradually over 10 days period following Cowan et al. (2020). For *S. macrospermum*, the same method was followed except that the water-stressed period was initiated using 47 days old plants and continued only for 18 days due to the slower growth of the plants. Plants were watered daily according to the requirements. For the water-stressed *S. macrospermum* plants, the weight of each individual pot was measured daily as the weights varied quite a lot.

The soil water capacity was assessed using the following equation (Hasanuzzaman et al. 2017). Target soil water content *W*_T*:*_

$$ W_{{\text{T }}} = W_{{\text{p}}} + W_{{\text{D}}} + \% {\text{RSWC}} \times W_{{\text{s}}} , $$

*W*_p_—weight of an empty pot, *W*_D_—dry soil weight, %RSWC relative soil water content (15% in this study), *W*_s_—soil water content (wet soil pot weight-dry soil pot weight).

The weight of the plants/pots was measured daily, and plants were watered according to the following equation

$$ {\text{Required amount of water}} = {\text{Target soil water content }} - {\text{Average actual weight}}{.} $$

Leaf samples were collected from each individual plant after 10 days of either well-watered or water-stressed growth (20 days old plants) and 47 days of water-stressed growth (57 days old plants) for *S. bicolor* and 10 days of either well-watered or water-stressed growth (57 days old plants) and 18 days water-stressed growth (65 days old plants) for *S. macrospermum* using destructive harvesting (Fig. S1b). The use of different harvest times for the two species was necessary due to their different growth rates. From each plant, the three youngest leaves which were fully expanded were collected. Each leaf was divided vertically along the mid rib into two equal parts and the left side was used for chemical analysis while the right side was used for RNA extraction. The material collected for chemical analysis was oven dried at 65 °C, then the three samples from each replicate were mixed and finely ground using a Qiagen Tissue Lyser II. The leaves collected for RNA extraction were immediately frozen in liquid nitrogen, briefly stored on dry ice, and then transferred to a − 80 °C freezer for long term storage. The leaves were pulverised using a Qiagen Tissue Lyser II under cryogenic conditions and stored at − 80 °C until RNA extraction.

### RNA extraction and sequencing

Total RNA was extracted from the pulverised leaf tissues of 36 sorghum samples (two time points and two treatments, five biological replicates for *S. bicolor* and four biological replicates for *S. macrospermum*) using an optimised methodology based on the Trizol—Qiagen RNAeasy mini kit (Furtado 2014). RNA was extracted separately from each of the three leaves harvested from each plant. The quality and quantity of the RNA were assessed using spectrometry with A260/280 and A260/230 absorbance ratios (NanoDrop, Thermofisher Scientific, USA) as well as using a 2100 Agilent Bioanalyzer (Agilent Technologies, Santa Clara, CA, USA) to monitor and secure 260/280 nm absorption ratios between 1.9 and 2.0 and RIN number above 6.5 for all RNA samples used for sequencing. All three RNA samples extracted from one replicate were combined affording a final RNA sample of 150–200 ng µL^−1^ that was sequenced at the Ramaciotti Centre, University of NSW, Australia using an Illumina NovaSeq 6000 platform.

### Transcriptome analysis and determination of gene expression levels

RNA-Seq analysis was performed using CLC Genomic Workbench (CLC-GWB) software (CLC Genomics Workbench 11.0, http://www.clcbio.com). Raw reads were imported to CLC-GWB, and the sequence quality trimmed at 0.01 quality limits (Phred score equivalent to > 20, 95% of the reads had a Phred score greater than 35, averaged across all bases) at a sequence length of 1000 bp. The trimmed reads were mapped to the reference mRNA transcriptome of *S. bicolor* (GCF_000003195.3). Then a count data table was generated in CLC, and differential gene expression was analysed for different comparison groups (Table S1). Results were filtered based on the FDR *p* value (≤ 0.01) and Principal Component Analysis (PCA) was conducted in CLC. An expression browser was created in CLC and exported to Omics Box (https://www.biobam.com/omicsbox/) for functional annotation and finally the Kyoto Encyclopedia of Genes and Genomes (KEGG) pathways were generated for the up- and down-regulated genes. A Venn diagram was drawn for the differentially expressed genes using the online tool in Bioinformatics and Evolutionary Genomics (http://bioinformatics.psb.ugent.be/webtools/Venn/). The top 10 up- and down-regulated genes identified in each comparison group were filtered based on the fold change values and KEGG pathway analysis was undertaken in Omics Box. Using the MapMan software (v. 3.0.0) (Thimm et al. 2004) metabolism overview and regulation overview of different comparison groups were designed. Furthermore, the differential expression of genes in the cyanogenesis pathway in sorghum (Table 1) was studied.

**Table 1:** Details of the genes which involve in cyanogenesis pathway in sorghum which were studied in this study

NCBI accession number	Plaza gene ID	Gene name
XM_002466054.2	Sobic_001G012300	Tyrosine *N*-monooxygenase—*CYP79A1*
XM_002466052.2	Sobic.001G012200	4-Hydroxyphenylacetaldehyde oxime monooxygenase-like—*CYP71E1*
XM_002463473.2	Sobic.001G012400	Cyanohydrin beta-glucosyltransferase—*UGT85B1*
XM_002441984.2	Sobic.008G079800	4-Hydroxy-7-methoxy-3-oxo-3,4-dihydro-2H-1,4-benzoxazin-2-yl glucoside beta-D-glucosidase 2—dhurrinase 1
XM_002443028.2	Sobic.008G080400	4-Hydroxy-7-methoxy-3-oxo-3,4-dihydro-2H-1,4-benzoxazin-2-yl glucoside beta-d-glucosidase 2—dhurrinase 2
XM_021446455.1	Sobic.008G080100	Dhurrinase—like 3
XM_021460447.1	Sobic.004G335500	*P*-(*S*)-hydroxymandelonitrile lyase—*HNL*
XM_002447428.2	Sobic.006G016900	Bifunctional L-3-cyanoalanine synthase/cysteine synthase 2, mitochondrial—*CAS C1*
XM_002452453.2	Sobic.004G225200	Bifunctional nitrilase/nitrile hydratase—*NIT4A*
XM_021459324.1	Sobic.004G225100	Bifunctional nitrilase/nitrile hydratase—*NIT4B2*
XM_002447182.2	Sobic.006G243200	Probable isoaspartyl peptidase/l-asparaginase 2
XM_002464220.2	Sobic.001G174700	Isoaspartyl peptidase/l-asparaginase 1
XM_021447365.1	Sobic.001G012600	*SbMATE2* (transporter)
XM_002464023.2	Sobic.001G133900	*SbCGTR1* (transporter)

To identify the source of the unmapped reads in RNA-Seq analysis, the unmapped reads were mapped against the ribosomal database SILVA (Quast et al. 2013) followed by the remaining unmapped reads mapping against the long noncoding RNA database (PNRD: Plant Non-coding RNAs database; cau.edu.cn). Then the remaining unmapped reads were subjected to de-novo assembly in CLC GWB and BLAST analysis and coding potential analysis was conducted with the resulting contigs in Omics Box.

### Chemical analysis

Hydrogen cyanide potential (HCNp), nitrate and phenylpropanoids were determined using finely ground oven-dried leaf tissue. HCNp is the total amount of HCN produced per unit plant material following addition of excess amounts of β-glucosidase (β-d-glucoside glucohydrolase, G4511, Sigma-Aldrich, Sydney, Australia) to ensure complete hydrolysis of the total amount of dhurrin present. Evolved HCN was determined colorimetrically (O’Donnell et al. 2013) as modified by Cowan et al. (2020). Each milligram of HCN is equivalent to 11.5 mg of dhurrin in the plant tissue. The nitrate concentration was determined colorimetrically (O’Donnell et al. 2013). Phenylpropanoids were measured using 10–20 mg dried tissue following Myrans et al. (2021).

### Statistical analysis

Results of the chemical analysis (HCNp, NO_3_^−^ and phenolics) were analysed using SigmaPlot v.14.5 (Systat Software) by two-way ANOVA, with the species and each stress treatment as the variables. Average values ± the standard error was plotted for *S. bicolor* (*n* = 5 for each treatment) and *S. macrospermum* (*n* = 4 for each treatment). Data sets were tested for normality (Shapiro–Wilk) and equal variance (Brown–Forsythe). All data sets passed these tests, except the analysis of nitrate in *S. macrospermum,* where reciprocal transformation was carried out. For all tests, a *p* value of < 0.05 was considered significant. When an interaction was detected, post-hoc comparisons were undertaken using Tukey’s test.

## Results

### Imposition and assessment of water-stress

**Fig. 1:**
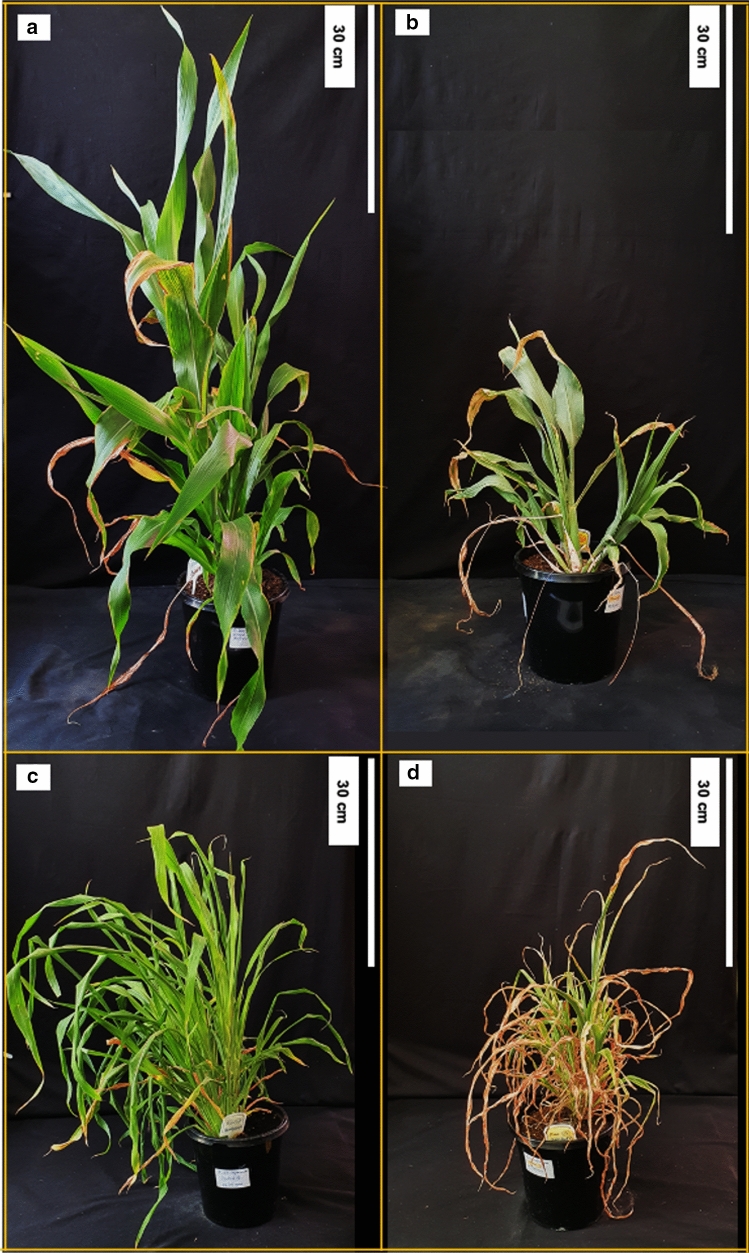
Sorghum plants grown under well-watered and water-stressed conditions. *S. bicolor* well-watered (control) (**a**) and water-stressed (47 days) (**b**). *S. macrospermum* well-watered (control) (**c**) and water-stressed (18 days) (**d**)

To monitor and adjust the level of imposed water-stress, the weight of randomly selected pots was measured daily, and the required amount of water added as described in the methods. According to the weight measurements, the water-stressed *S. bicolor* plants had a lower actual weight than the target weight for the day. The daily water usage of the *S. macrospermum* plants was higher than that of the *S. bicolor* plants (Fig. S1a). This is likely due to the higher number of tillers formed by the *S. macrospermum* plants. Compared to the *S. bicolor* plants, the growth of the *S. macrospermum* plants was slower. Accordingly, identical water-stress periods could not be maintained for the two species. In both species, the water-stress treatment was effective with the water-stressed plants being smaller than the well-watered plants and carrying severely wilted leaves (Fig. 1).

### Transcriptome analysis and determination of gene expression levels

RNA-Seq followed by differential gene expression analysis was performed for *S. bicolor* and *S. macrospermum* to compare gene expression profiles at different time points and growth conditions within and between the two species (Table S1). In RNA-Seq, the average mapping percentage of the paired-end trimmed reads was 37% (Table S2). The unmapped reads were mapped against the ribosomal database SILVA (Quast et al. 2013) affording a mapping percentage of 15% of the total reads and against the long noncoding RNA database (PNRD: plant non-coding RNAs database; cau.edu.cn) affording an additional mapping percentage of 15% of the total reads. The remaining 33% of the total reads were mapped against the whole genome of *S. bicolor.* The total mapping percentage was therefore 99% (Table S3).

In the BLAST analysis of the contigs, 71% of the contigs had no BLAST hits whereas in the coding potential analysis only 50% of the contigs had complete Open Reading Frames (ORF) while the rest had 3′ or 5′ partial ORFs (Table S4).

### Age and water-stress result in differential expression of distinct genes

**Fig. 2:**
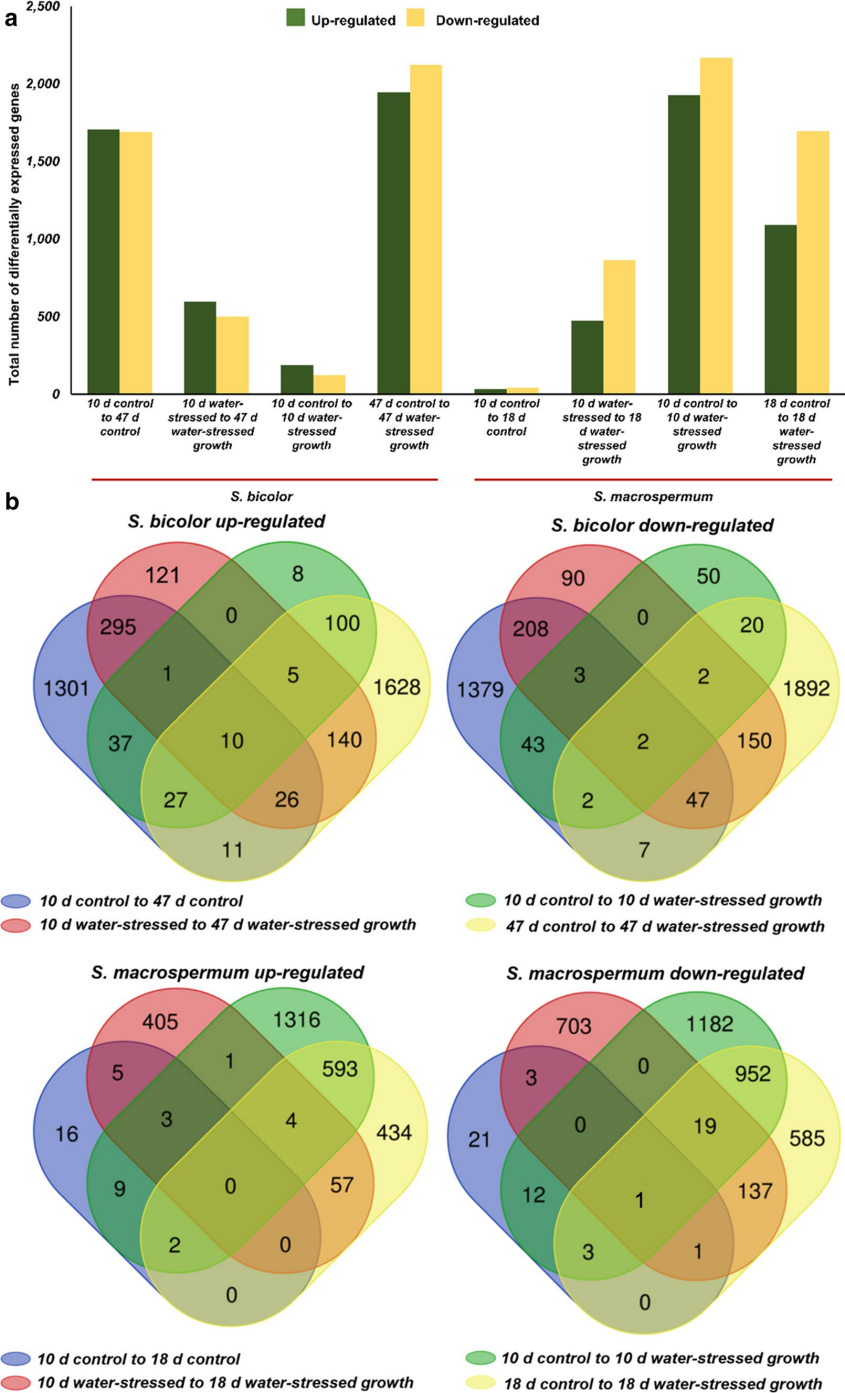
Comparison of differentially expressed genes within and between *S. bicolor* and *S. macrospermum* depending on plant tissue age and imposed water-stressed growth illustrated by the number of up- and down-regulated genes (**a**) and using Venn diagrams (**b**)

Differential gene expression analysis was performed at the FDR *p* value ≤ 0.01 for each comparison group. Comparison of the transcript profiles of *S. bicolor* control at day 47 (47 days) and water-stressed at 47 days showed 4069 differentially expressed genes (DEGs). This is the highest number of DEGs observed between the analysed groups and demonstrate a highly complex response to long term water-stressed growth. The lowest number of DEGs was observed between the *S. macrospermum* control at day 10 and day 18 (18 days). The 76 DEGs identified indicate that an 8 day additional growth under well-watered conditions results in minute changes in the gene expression profile. The number of up- and down-regulated DEGs within and between *S. bicolor* and *S. macrospermum* independent of plant age and imposed water-stressed growth is shown on Fig. 2a.

Analysis of the Venn diagrams identifies 10 DEGs in *S. bicolor* that were up-regulated in all treatments and 2 DEGs that were down-regulated (Fig. 2b and Table S7). In *S. macrospermum*, a single DEG was down-regulated in all treatments whereas none of the DEGs were up-regulated in all treatments (Fig. 2b, Table S7). In the water-stressed *S. bicolor* plants at 10 days and 47 days (Fig. 2b), 8 and 1628 DEGs were up-regulated exclusively whereas 50 and 1892 DEGs were down-regulated (Fig. 2b), respectively. Similarly, 1316 and 434 DEGs were up-regulated in the water-stressed *S. macrospermum* plants at 10 days and 18 days (Fig. 2b), while 1,182 and 585 genes were exclusively down-regulated (Fig. 2b), respectively.

**Fig. 3:**
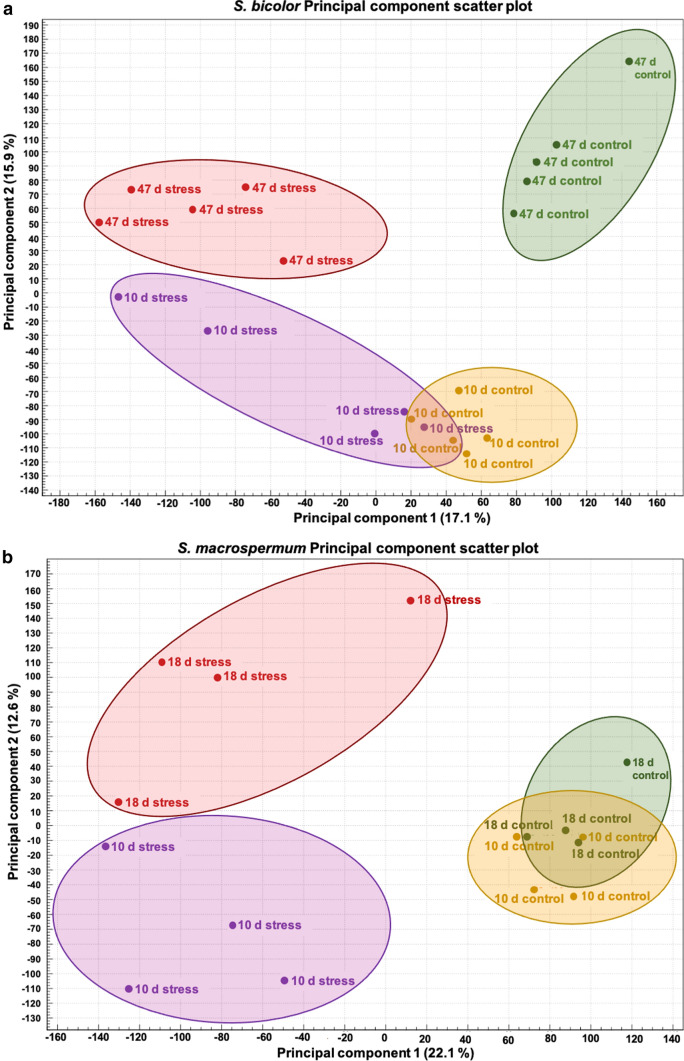
Principal component scatter plots of differentially expressed genes in *S. bicolor* and *S. macrospermum* depending on plant tissue age and imposed water-stressed growth. **a** *S. bicolor* principal component scatter plot. **b** *S. macrospermum* principal component scatter plot

Principal component scatter plots of DEGs in *S. bicolor* and *S. macrospermum* show that the total variance present in the data set was 40.4% and 44.1%, respectively. In *S. bicolor*, PC1 contributed to 17.1%, PC2 contributed to 15.9% and the PC3 contributed to 7.4% of the total variance (Fig. 3a). The corresponding values in *S. macrospermum* were 22.1%, 12.6% and 9.4%. PCA was able to differentiate the samples based on plant age and water-stressed growth. PCA was not able to differentiate the 10 days control and water-stressed growth samples in *S. bicolor* and the 10 days and 18 days control samples in *S. macrospermum* (Fig. 3b).

### KEGG pathway analysis of top 10 DEGs

The top 10 up- and down-regulated DEGs from each of the comparison groups were selected based on the fold change values of all the comparison groups and KEGG pathway analysis was used to identify the associated pathways. Up-regulated DEGs were associated with common pathways such as purine and thiamine metabolism pathways in the comparison groups of *S. bicolor* control 10 days vs 47 days, *S. bicolor* water-stressed growth 10 days vs 47 days, *S. bicolor* 47 days control vs water-stressed growth, and *S. macrospermum* control 10 days vs 18 days. Down-regulated DEGs were linked to purine and thiamine metabolism in the comparison groups of *S. macrospermum* control 10 days vs 18 days and *S. macrospermum* water-stressed growth 10 days vs 18 days. No KEGG defined pathways could be associated with the top 10 up- or down-regulated genes in *S. bicolor* control 47 days vs water-stressed growth 47 days (Table 2). The details of the top 10 up- and down-regulated DEGs are given in the supplementary information (Tables S5 and S6).

**Table 2:** Details of the KEGG pathways associated with the top 10 up- and down-regulated genes in each of the comparison groups

Comparison group	KEGG Pathways associated with	
	Top 10 up-regulated genes	Top 10 down-regulated genes
*S. bicolor* 10 days control to 47 days control	Photosynthesis, steroid hormone biosynthesis, steroid biosynthesis, purine metabolism, steroid degradation, thiamine metabolism	Ubiquinone and other terpenoid-quinone biosynthesis, biosynthesis of co-factors
*S. bicolor* 10 days water-stressed to 47 days water-stressed growth	Purine metabolism, vitamin B6 metabolism, glycerophospholipid metabolism, thiamine metabolism	Nitrogen metabolism
*S. bicolor* 10 days control to 10 days water-stressed growth	Starch and sucrose metabolism, MAPK signalling pathway, Plant hormone signal transduction	Plant pathogen interaction
*S. bicolor* 47 days control to 47 days water-stressed growth	None	Steroid biosynthesis, diterpenoid biosynthesis
*S. macrospermum* 10 days control to 18 days control	Purine metabolism, thiamine metabolism, MAPK signalling pathway, plant hormone signal transduction	Purine metabolism, thiamine metabolism, protein processing in endoplasmic reticulum, lysine degradation
*S. macrospermum* 10 days water-stressed to 18 days water-stressed growth	Steroid biosynthesis, RNA transport	Purine metabolism, butanoate metabolism, alanine, aspartate and glutamate metabolism, sulphur metabolism, thiamine metabolism, biotin metabolism, protein processing in endoplasmic reticulum
*S. macrospermum* 10 days control to 10 days water-stressed growth	Metabolism of xenobiotics by cytochrome P450, glycolysis/gluconeogenesis, tyrosine metabolism, pyruvate metabolism, biotin metabolism, butanoate metabolism, naphthalene degradation, chloroalkane and chloroalkene degradation, alpha-linolenic acid metabolism, glycine, serine and threonine metabolism, fatty acid degradation, alanine, aspartate and glutamate metabolism, retinol metabolism, methane metabolism	Pentose and glucuronate interconversions
*S. macrospermum* 18 days control to 18 days water-stressed growth	Flavonoid biosynthesis, phenylpropanoid biosynthesis, stilbenoid, diarylheptanoid and gingerol biosynthesis, riboflavin metabolism, thiamine metabolism	Protein processing in endoplasmic reticulum, lysine degradation

Among the top 10 up- and down-regulated genes from all eight comparison groups, 50% and 37.5% of the up- and down-regulated genes in *S. bicolor*, respectively*,* were functionally uncharacterized. In *S. macrospermum*, the corresponding numbers of genes with unknown functions were 27.5% and 30%. Among these top 10 DEGs, the only commonly expressed gene in both the species was XM_021447547.1, which encodes a late embryogenesis abundant (LEA) protein. Some of the individual comparison groups include DEGs encoding LEA proteins highly up-regulated upon water-stressed growth in *S. bicolor* (gene ID: XM_021447547.1 and XM_002444176.2) as well as in *S. macrospermum* (gene ID: XM_021448835.1), which have been linked with water-stress in other studies (Johnson et al. 2014). In addition, genes which encode lipid transfer proteins, ATP binding proteins, chromatin regulators, electron transfer proteins, cysteine type peptidases, ion binding proteins, transcription regulators and proteins involved in protein metabolism pathway are highly differentially expressed in all individual comparison groups (Tables S5 and S6). Supplementary Figs. S2–S9 provide a metabolism-based overview of the genes which are differentially expressed in *S. bicolor* and *S. macrospermum* independent of plant age and imposed water-stress. These results match the results of the PCA (Fig. 3) by showing few differences in gene expression between *S. bicolor* control 10 days and water-stressed growth 10 days (Fig. S4) and *S. macrospermum* control 10 days and 18 days (Fig. S6). Marked differences in the expression of genes related to photorespiration, C1-metabolism, lipid metabolism and mitochondrial electron transport were observed dependent on plant tissue age and imposed water-stressed growth (Figs. S2–S9).

### Role of hormones in water-stress response

**Fig. 4:**
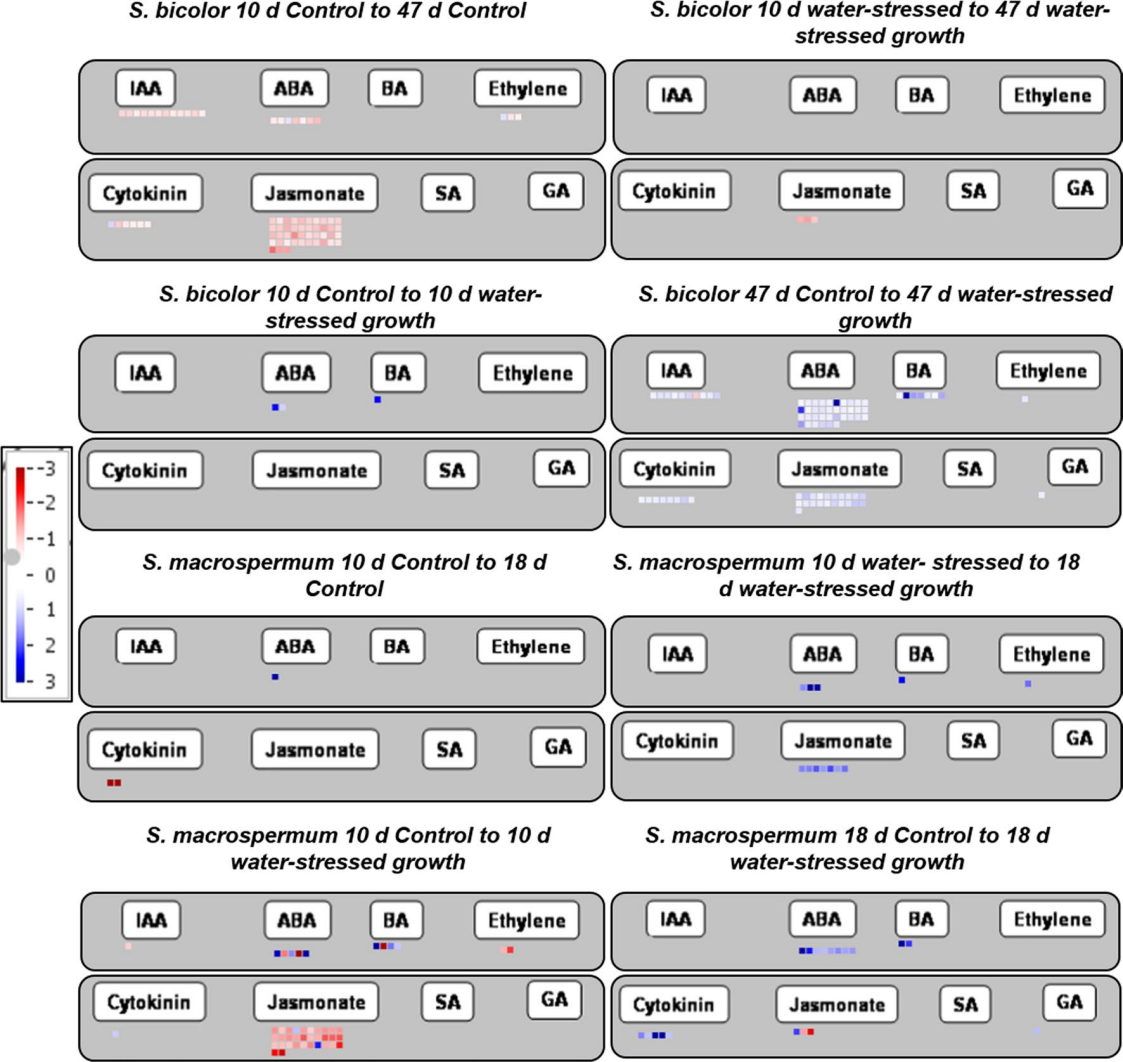
The effect of plant tissue age and water-stressed growth on the transcript levels of genes involved in plant hormone metabolism in *S. bicolor* and *S. macrospermum*. Red: down-regulated gene transcript, Blue: up-regulated gene transcript

An overview of the effect of plant tissue age and water-stressed growth on the transcript levels of genes involved in plant hormone metabolism is presented in Fig. 4. Genes which encode enzymes catalysing biosynthesis of abscisic acid (ABA), benzyl adenine (BA), cytokinin, indole acetic acid (IAA), jasmonate, ethylene and gibberellic acid were highly expressed in water-stressed *S. bicolor* plants. In older plant tissues, the genes which produce IAA, jasmonate and ABA were down-regulated. In *S. macrospermum*, genes which regulate ABA, BA, cytokinin, jasmonate, and ethylene production were up-regulated in water-stressed plants in young as well as older tissues (Fig. 4), except for the jasmonate biosynthetic transcripts in the 10 days experiment which were down-regulated.

### Differential expression of genes involved in cyanogenesis

**Fig. 5:**
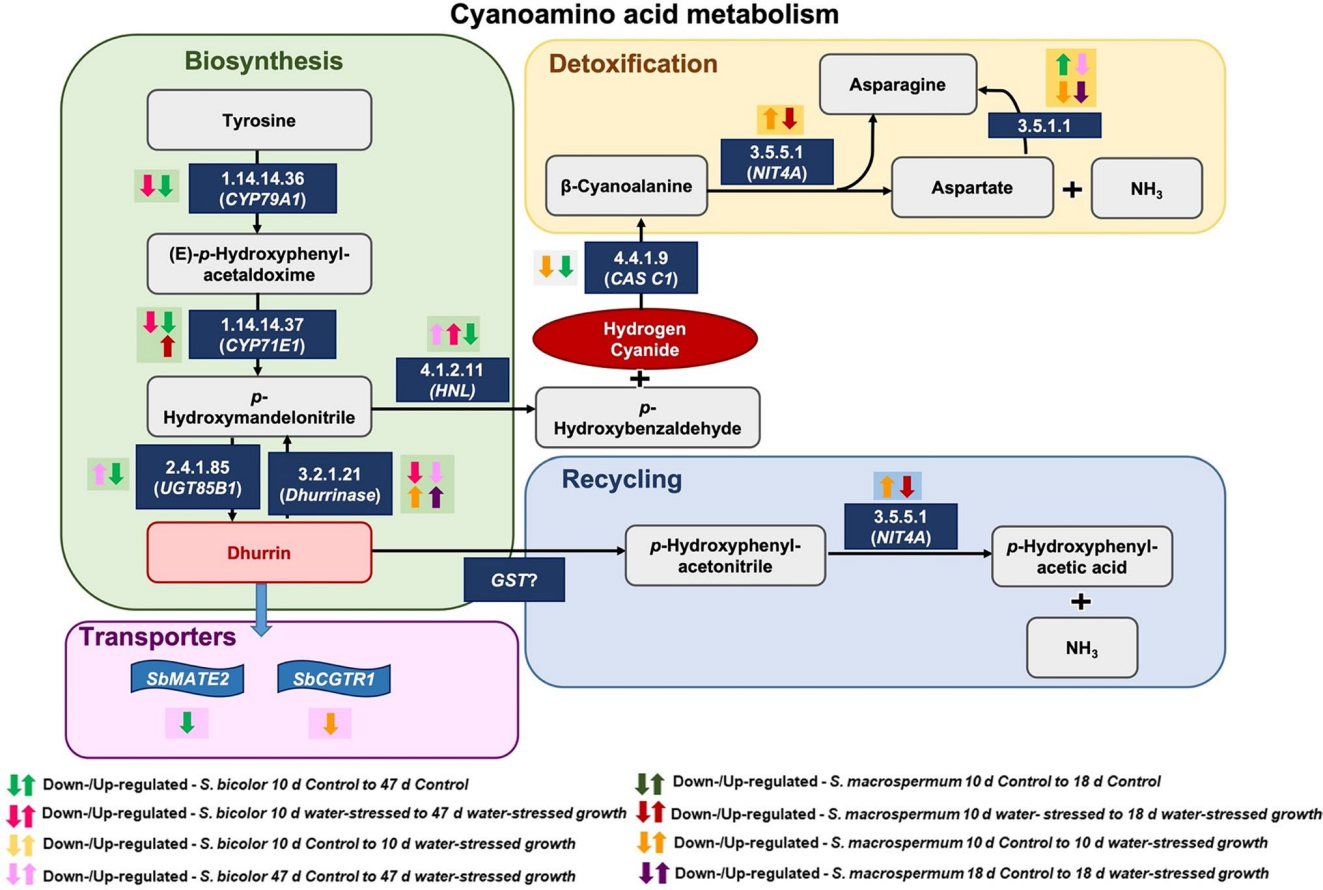
Differentially expressed genes under different conditions in the cyanoamino acid metabolism pathway in sorghum. Different colours of arrows represent different comparison groups. The up arrows indicate up-regulated genes and down arrows indicate the enzyme codes in Omics box

The expression values and the transcripts per million of the selected cyanogenesis related genes for the two species under all conditions are given in the supplementary table S8. The expression values of the cyanogenesis genes were comparatively much higher in the *S. bicolor* samples relative to the *S. macrospermum* samples (Table S8). The genes which are differentially expressed in the cyanogenesis pathway were determined by KEGG pathway analysis for each comparison group in both *S. bicolor* and *S. macrospermum*. In *S. bicolor*, between the control plants at 10 days and 47 days, the genes *CYP79A1* (1.14.14.36), *CYP71E1* (1.14.14.37), *UGT85B1* (2.4.1.85), *HNL* (4.1.2.11), and cysteine synthase 2 (*CAS C1*) (4.4.1.9), were down-regulated whereas isoaspartyl peptidase/l-asparaginase 2 (3.5.1.1) was up-regulated. Compared to the water-stressed plants at 10 days, the genes *CYP79A1* (1.14.14.36), *CYP71E1* (1.14.14.37), and beta-glucosidase (3.2.1.21) were down-regulated while *HNL* (4.1.2.11) was up-regulated in the water-stressed grown plants at 47 days. In the comparison between the 10 days control and water-stressed plants, none of the genes related to dhurrin synthesis, recycling or bio-activation were differentially expressed. In water-stressed compared to control plants at 47 days, isoaspartyl peptidase/l-asparaginase 1/2 (3.5.1.1) and dhurrinase-1 (3.2.1.21) were down-regulated whereas *UGT85B1* (2.4.1.85)*, HNL* (4.1.2.11) were up-regulated. Furthermore, the dhurrin transporter *SbMATE2* was down-regulated in the 47 days control plants compared to the 10 days control plants (Table S9, Fig. 5). In *S. macrospermum*, none of the genes in the dhurrin biosynthetic pathway were differentially expressed in the control plants at 18 days compared to the control plants at day 10. However, the gene *NIT4A* (3.5.5.4/1) was down-regulated whereas *CYP71E1* (1.14.14.37) was up-regulated in the water-stressed 18 days plants relative to the water-stressed 10 days plants. Compared to the control 10 days plants, the genes cysteine synthase 2 (*CAS C1*) (4.4.1.9), and isoaspartyl peptidase/l-asparaginase 1/2 (3.5.1.1) were down-regulated while dhurrinase-1, dhurrinase-2 and dhurrinase-like 3 (3.2.1.21)*,* and *NIT4A* (3.5.5.4/1) were up-regulated in the water-stressed plants at 10 days. In the final combination of the control 18 days plants relative to water-stressed 18 days plants, the isoaspartyl peptidase/l-asparaginase 1/2 (3.5.1.1) gene was down-regulated whereas dhurrinase-1, dhurrinase-2 and dhurrinase-like 3 (3.2.1.21) were up-regulated Moreover, the putative dhurrin transporter *SbCGTR1* was down-regulated in the 10 days water-stressed growth compared to the 10 days control plants (Table S9, Fig. 5).

### Chemical analysis

**Fig. 6:**
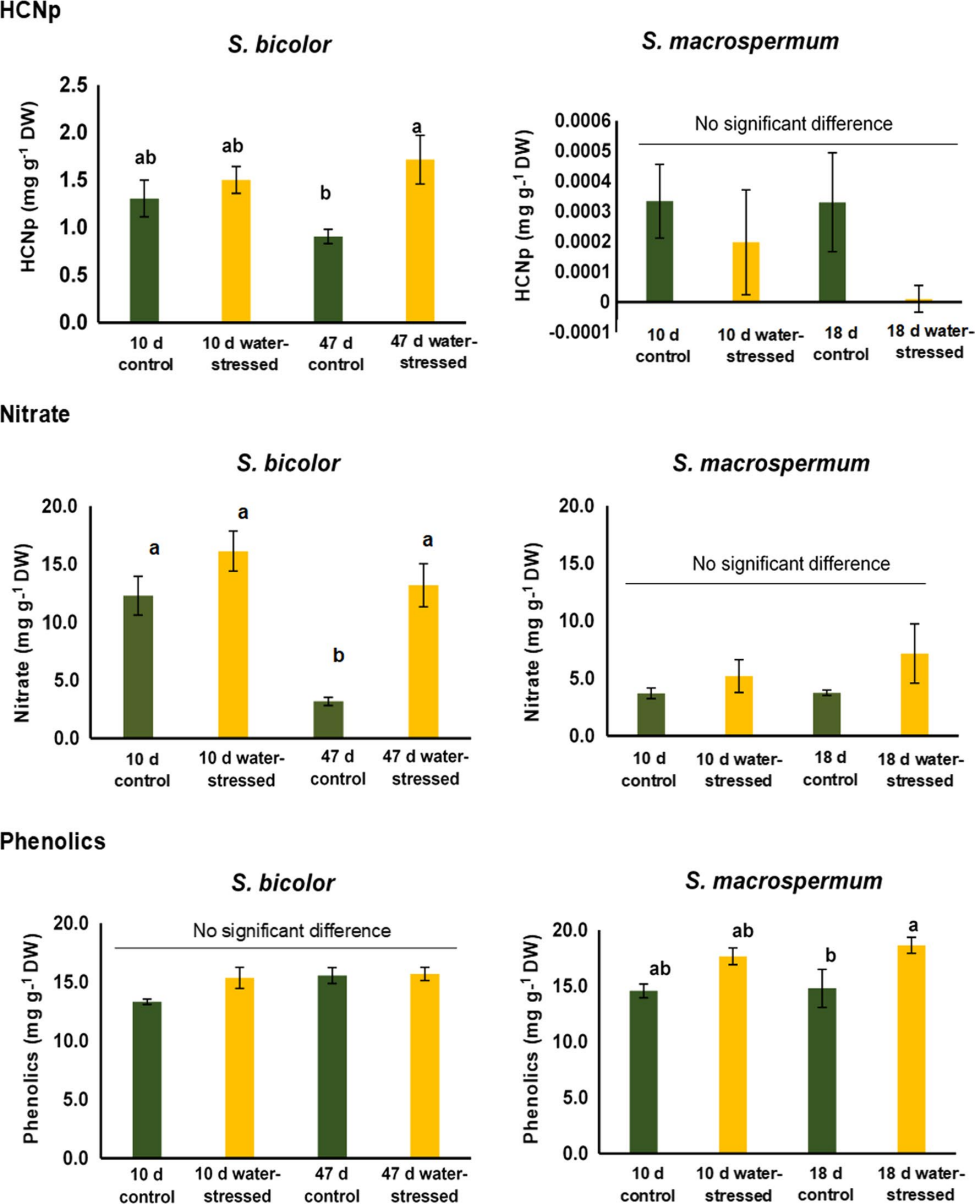
The effect of plant age and water-stress on the HCNp, nitrate and phenylpropanoid content in *S. bicolor* and *S. macrospermum*. Columns marked with different letters are significantly different (*p* < 0.05)

The average HCNp of leaf tissues of sorghum plants was measured under control and water-stressed growth conditions as a means to assess the total content of dhurrin. At all full-watered and water-stressed conditions tested, the dhurrin content of the *S. bicolor* leaves was around 1000-fold higher than that of the corresponding *S. macrospermum* leaf samples. In *S. bicolor*, the dhurrin level decreased with tissue age but showed significant increases under water-stressed growth conditions. The highest dhurrin levels were found in leaves of *S. bicolor* harvested from 47 days water-stressed plants. In *S. macrospermum* leaves, the dhurrin levels were minute and showed no significant changes with plant age and following water-stressed growth conditions (Fig. 6).

The average NO_3_ concentration was comparatively higher in *S. bicolor* tissues, with a significant difference between control and water-stressed plants at 47 days with lower NO_3_ concentrations in the well-watered plants (Fig. 6). There were no significant differences between the NO_3_ concentrations in the *S. macrospermum* samples.

The total phenylpropanoid content in *S. bicolor* leaves did not show significant changes with tissue age and water-stressed growth (Fig. 6). In *S. macrospermum*, the total content of phenylpropanoids showed a significant increase in the 10 days water-stressed leaf tissues but did not increase with extended growth under water-stressed conditions (Fig. 6).

## Discussion

Plants respond to different biotic and abiotic stresses by altering their gene expression profiles as monitored by transcriptome analysis (Takahashi et al. 2018; Shankar et al. 2013). Water-stress increases the dhurrin content in leaves of domesticated sorghum (O'Donnell et al. 2013) but not in the leaves of wild sorghum (Cowan et al. 2020). Several studies of the effects of biotic and abiotic stresses have also been carried out on other cyanogenic species, including cassava (Brown et al. 2016), lima bean (Ballhorn et al. 2011) and eucalypt (Gleadow and Woodrow 2002a). However, there have been no studies investigating the differences in gene expression in wild sorghum species in response to water-stress. In the current study, growth of the domesticated sorghum species *S. bicolor* was shown to differ from that of the wild sorghum species *S. macrospermum* plants. Under the glasshouse conditions used, the *S. macrospermum* plants grew more slowly than the *S. bicolor* plants. As a result of producing more tillers, the transpiration rate in *S. macrospermum* plants was higher than that of *S. bicolor,* which led to a higher daily water usage. *S. macrospermum* plants were severely affected by the water-stressed growth conditions and showed more severe symptoms compared to the *S. bicolor* plants.

Differential gene expression analysis following imposed water-stressed growth conditions showed down-regulation of the expression of many genes suggesting that many of metabolic processes were slowed down or stopped due to the stress. Comparison of the differential gene expression between the control treatments of both species at the two time points show approximately the same number of up and down regulated genes within each species. These genes would be involved in general plant metabolism related to growth and development and the total number is much higher in *S. bicolor* than in *S. macrospermum*. The Venn diagrams illustrate the number of unique genes expressed in water-stressed plants of both species. Many of the genes were involved in functions such as protein binding and ion binding but further analysis is required to identify their specific roles related to water-stress. In the PCA, the variance between the control 10 days and water-stressed 10 days plants of *S. bicolor* and between control 10 days and 18 days plants of *S. macrospermum* were not able to differentiate them into separate groups. As reflected by the metabolism overview also, there is not much difference in gene expression levels in response to short-term water-stressed growth in younger *S. bicolor* plants. The high number of differentially expressed genes in the *S. bicolor* water-stressed plants at 47 days compared to the control plants indicate a complex response to long term water-limitation. In *S. macrospermum* control plants, the difference in gene expression profiles with age were minor.

Plant hormones control the growth of the plant under stress conditions (Miransari 2016; Hasanuzzaman et al. 2013). This study highlights the differential expression of genes which regulate stress related hormones. ABA is a plant hormone that improves resistance to drought stress (Ullah et al. 2018) by regulating stomatal activities, protein and lipid synthesis, root development and leaf senescence (Tuteja 2007). Drought and salinity stress are reported to increase ABA levels in plants (Hasanuzzaman et al. 2013) and thereby regulate the function of aquaporins (Li et al. 2014) and enhance root growth (Lynch and Brown 2008). Jasmonic acid, ethylene, IAA, gibberellins, and cytokinins play other important roles in drought stress tolerance (Ullah et al. 2018). Thus, IAA enhances drought tolerance by increasing ABA and jasmonic content and activating auxin responsive genes in white clover (Zhang et al. 2020). Further studies are required to understand the relationship of these genes with the age of sorghum plants as different responses were observed between the young and old tissues of the two species.

KEGG pathway analysis was performed on the top 10 up- and down-regulated genes in each comparison group in an attempt to identify metabolic pathways in which these highly differentially expressed genes were involved. Among these genes, only the gene XM_021447547.1, which encodes a LEA protein was commonly expressed in both species while the others were uniquely expressed. In addition, and in agreement with previous studies (Johnson et al. 2014), we report that genes encoding LEA proteins were among the top 10 up-regulated genes in stressed plants of both the species with very high fold change values. LEA proteins are a group of hydrophilic proteins which accumulate under water deficit conditions (Battaglia and Covarrubias 2013) and act e.g. by preventing inactivation of lactate dehydrogenase enzymes (Reyes et al. 2008), stabilizing membranes (Stacy and Aalen 1998), and supporting ion sequestration (Hara et al. 2005). Furthermore, lipid transfer proteins were up-regulated in the *S. bicolor* water-stressed plants (Johnson et al. 2014).

Cyanogenesis in forage sorghum can be problematic, especially in countries like Australia where sorghum is an important source of forage (Cowan et al. 2020). The HCNp is high in young plants, in heavily fertilised plants and in sorghum exposed to drought (O’Donnell et al. 2013). Efforts have been made to develop low-cyanide sorghum using mutagenesis (Blomstedt et al. 2012). Another option is to exploit the naturally low dhurrin content of other species of sorghum in the tertiary gene pool (Cowan et al. 2020). In the present study we demonstrate that, even under severe stress, *S. bicolor* leaves have around a 1000-fold higher HCNp than the wild sorghums. In agreement with previous studies, a high HCNp was found in young *S. bicolor* plants and that the HCNp increased with stress in older *S. bicolor* plants (Cowan et al. 2020; Blomstedt et al. 2018; Gleadow et al. 2016). The high concentration of dhurrin in stressed *S. bicolor* plants may be a direct stress response. *S. macrospermum* plants showed an opposite response to water-stressed growth by maintaining very low concentrations of dhurrin in their leaf tissue independent of tissue age or imposed water-stress, although the low dhurrin concentrations made exact comparisons difficult.

Nitrate concentrations in plants are dependent on the genotype, tissue type, and water availability (Rosati et al. 2019a). Our results show that the nitrate concentration in leaves was higher in domesticated sorghum than the wild sorghum plants. Nitrate concentration increased at water-stressed growth in both species but only significantly in *S. bicolor*. The rate of nitrate uptake from the soil and nitrate reductase enzyme activity are key determinants of the nitrate concentration in plants (Rosati et al. 2019a). Studies have shown that drought stress reduces nitrate reductase activity (Fresneau et al. 2007; Kaiser and Huber 2001).

Phenylpropanoids are a large class of natural products. They are known to provide protection against biotic and abiotic challenges (Myrans et al. 2021; Varela et al. 2016; Hura et al. 2008). Drought-stress increases the content of phenylpropanoids in *Populus euphratica* (Guignard et al. 2005), *Populus nigra* (Hale et al. 2005), and *Amaranthus tricolor* (Sarker and Oba 2018). In the current study, water-stressed *S. macrospermum* plants had a significantly higher content of phenylpropanoids indicating that they also play a role in stress tolerance in wild sorghums. The metabolic relationship between the formation of dhurrin and other phenylpropanoids requires further investigation.

In the comparative study of transcript levels of genes responsible for the biosynthesis of dhurrin and its recycling and bio-activation in domesticated and wild sorghum subjected to water-stressed growth (Fig. 5), most of the gene expression profiles obtained mirrored the analyses of dhurrin content. None of the genes involved in these processes were registered among the top 10 DEGs in the eight comparison groups (Table 1). *CYP79A1* (1.14.14.36) is the first committed enzyme in dhurrin biosynthesis, and its transcript shows a 19.4- and 4.9-fold higher expression in the leaves of 10 days *S. bicolor* seedlings in comparison with leaves from 47 days old sorghum plants grown under well-watered and water-stressed conditions, respectively (Table S9). *CYP71E1* (1.14.14.37) is the second enzyme in the dhurrin pathway and the corresponding fold reduction in its transcript is 3.9 and 1.4-fold, implying that dhurrin formation shows an age dependent decrease and is induced as a result of water-stress (Fig. 5) in agreement with previous data (Cowan et al. 2020; Busk and Møller 2002; Gleadow and Woodrow 2002b). Dhurinase2, the key gene in the bio-activation pathway was down-regulated in the older leaves as was *CAS1* which is active in preventing auto-toxicity from released HCN (Table S9).

The dhurrin content of the wild sorghum *S. macrospermum* is minute and no significant increase was observed water-stressed growth conditions (Fig. 6). No significant expression was observed for the gene *CYP79A1*. Nevertheless, significant increased expression of the *CYP71E1* gene involved in the biosynthesis of dhurrin and of several dhurrinase genes was observed under water-stressed conditions. The *CAS C1* and *NIT4A* genes are involved in detoxification of HCN resulting from bio-activation of dhurrin as well as HCN release concomitant with the synthesis of ethylene (Jenrich et al. 2007). This may explain the observed shifting expression patterns. The increased expression of the transcripts encoding dhurrinase-1 and 2 and of dhurrinase-like 3 under water-stressed growth implies that these β-glycosidases may have other in vivo substrates than dhurrin.

Although, some studies have indicated that the wild sorghum species are more tolerant to drought (Cowan et al. 2020), we observed that the wild sorghum *S. macrospermum* is more susceptible to water-stressed growth and grows slower than domesticated *S. bicolor*. It might be that wild sorghum species were more adapted to natural harsh environments and show a different pattern of growth under artificial conditions.

Cyanogenesis is believed to come at a metabolic cost to the plant by utilizing the resources which could be used in plant growth and development (Herms and Mattson 1992). Although, cultivated sorghum plants use cyanogenesis as an herbivore defence mechanism, wild sorghum leaves might have roles other than defence. *S. macrospermum* is endemic to northern Australia, and the low nitrogen availability of the soil (Dillon et al. 2007) in their habitat, might result in the available nitrogen being utilized in general metabolic and growth process rather than cyanogenesis also, dhurrin may be instantly converted into free nitrogen (Cowan et al. 2022). Therefore, wild sorghum species have low dhurrin content in the leaves and the plants might rely on other defence mechanisms such as trichomes on the leaf surface (Johnson 1975).

Identification of potential candidate genes (transcription factors) that control the differential regulation of dhurrin biosynthesis in domesticated and wild sorghum is a vital step in developing more drought tolerant sorghum species. On the other hand, wild sorghum species such as *S. macrospermum* with a very low dhurrin content in their leaves can be “redomesticated” by introducing genes related to higher yield and biomass from the already domesticated sorghum species e.g., as shown in wild tomato (Lemmon et al. 2018; Zsögön et al. 2018). The close phylogenetic relationship of domesticated *S. bicolor* and the wild species *S. macrospermum* (Ananda et al. 2021) provides a great advantage to the introgression of beneficial traits of sorghum species.

## Conclusions

In this study, we identified genes which are differentially expressed in response to water-stress in domesticated and wild sorghum species and specifically highlighted the different expression patterns of the genes in the dhurrin synthesis and bio-activation pathways in response to water-stressed growth conditions. Under all conditions tested, the dhurrin content was around 1000-fold higher in *S. bicolor* leaves as compared to *S. macrospermum* leaves. In *S. bicolor*, dhurrin concentration decreased with age and increased with stress while in *S. macrospermum* the levels remained minute. Beneficial traits of *S. macrospermum* such as its acyanogenic leaves may be introgressed into domesticated sorghum to provide safe fodder for livestock especially under conditions of drought.

### *Author contribution statement*

RJH: conceptualization, methodology, supervision, project administration, funding acquisition, resources and writing—review and editing. RG: conceptualization, supervision, funding acquisition, writing—review and editing. BLM: conceptualization, funding acquisition, writing—review and editing. AF: methodology, formal analysis, supervision, data curation, writing—review and editing. SLN: methodology, resources and writing—review and editing. CB: methodology, data collection, formal analysis, resources and writing—review and editing. GKSA: formal analysis, data curation, investigation, writing—original draft.

## Supplementary Information

Below is the link to the electronic supplementary material.

Supplementary file1 (DOCX 27303 KB)

## Data Availability

All data and materials used and described in this study are made available for non-commercial research purposes. The data that support the findings of this study are openly available in Sequence Read Archive (SRA) under the BioProject number PRJNA736757 (http://www.ncbi.nlm.nih.gov/bioproject/736757).

## Abbreviations

ABA: Abscisic acid
AGG: Australian Grains Genebank
AQP: Aquaporins
BA: Benzyl adenine
BGD: β-Glucosidase
CAS: β-Cyanoalanine synthase
CLC-GWB: CLC Genomic Workbench
CNglcs: Cyanogenic glycosides
d: Day
DEGs: Differentially expressed genes
DHN: Dehydrin proteins
DREB: Drought-responsive element binding proteins
HCN: Hydrogen cyanide
HCNp: Hydrogen cyanide potential
HNL: α-Hydroxynitrilase
IAA: Indole acetic acid
KEGG: Kyoto Encyclopedia of Genes and Genomes
LEA: Late embryogenesis abundant protein
NIT4: Nitrilase 4
ORF: Open reading frames
PCA: Principal component analysis
POR: P450 oxidoreductase
UGT: UDP-glucosyltransferase
UQ: University of Queensland
