# Supplementary material for: Transcript profiles of wild and domesticated sorghum under water-stressed conditions and the differential impact on dhurrin metabolism
Source: Planta. 2022 Jan 27;255(2):51. doi: 10.1007/s00425-022-03831-4 (PMC8795013; doi:10.1007/s00425-022-03831-4)
Supplement: Supplementary file 1 — Supplementary file1 (DOCX 27303 KB) [file 425_2022_3831_MOESM1_ESM.docx]

**Supplementary Materials**

**Transcript profiles of wild and domesticated sorghum under water-stressed conditions and the differential impact on dhurrin metabolism**

Galaihalage K. S. Ananda^1^, Sally L. Norton^2^, Cecilia Blomstedt^3^, Agnelo Furtado^1^, Birger Lindberg Møller^4^, Roslyn Gleadow^1,3^ and Robert J. Henry^1*^

^1^ Queensland Alliance for Agriculture and Food Innovation, The University of Queensland, St Lucia, QLD, Australia,

^2^ Australian Grains Genebank, Agriculture Victoria, Horsham, Vic, Australia,

^3^ School of Biological Sciences, Monash University, Clayton, Vic, Australia,

^4^ Plant Biochemistry Laboratory, Department of Plant and Environmental Sciences, University of Copenhagen, Copenhagen, Denmark

**Correspondence:**

Robert J. Henry, Queensland Alliance for Agriculture and Food Innovation, The University of Queensland, St Lucia, QLD, Australia

robert.henry@uq.edu.au

**Supplementary Data**

There is one supplementary file at Planta containing 9 figures and 9 tables.

**Table S1:** Details of samples included in each comparison groups

| **Comparison group** | **Samples** | **Comparing condition** |
| --- | --- | --- |
| *S. bicolor 10 d Control to 47 d Control* | 01SbC10P1Rep1, 02SbC10P2Rep2, 03SbC10P3Rep3, 04SbC10P4Rep4, 05SbC10P5Rep5, 11SbC47P6Rep1, 12SbC47P7Rep2, 13SbC47P8Rep3, 14SbC47P9Rep4, 15SbC47P10Rep5 | Age |
| *S. bicolor 10 d water-stressed to 47 d water-stressed growth* | 06SbS10P1Rep1, 07SbS10P2Rep2, 08SbS10P3Rep3, 09SbS10P4Rep4, 10SbS10P5Rep5, 16SbS47P6Rep1, 17SbS47P7Rep2, 18SbS47P8Rep3, 19SbS47P9Rep4, 20SbS47P10Rep5 | Age and Stress |
| *S. bicolor 10 d Control to 10 d water-stressed growth* | 01SbC10P1Rep1, 02SbC10P2Rep2, 03SbC10P3Rep3, 04SbC10P4Rep4, 05SbC10P5Rep5, 06SbS10P1Rep1, 07SbS10P2Rep2, 08SbS10P3Rep3, 09SbS10P4Rep4, 10SbS10P5Rep5 | Stress |
| *S. bicolor 47 d Control to 47 d water-stressed growth* | 11SbC47P6Rep1, 12SbC47P7Rep2, 13SbC47P8Rep3, 14SbC47P9Rep4, 15SbC47P10Rep5, 16SbS47P6Rep1, 17SbS47P7Rep2, 18SbS47P8Rep3, 19SbS47P9Rep4, 20SbS47P10Rep5 | Stress |
| *S. macrospermum 10 d Control to 18 d Control* | 21SmC10P1Rep1, 22SmC10P2Rep2, 23SmC10P3Rep3, 24SmC10P4Rep4, 29SmC18P5Rep1, 30SmC18P6Rep2, 31SmC18P8Rep3, 32SmC18P9Rep4 | Age |
| *S. macrospermum 10 d water- stressed to 18 d water-stressed growth* | 25SmS10P1Rep1, 26SmS10P2Rep2, 27SmS10P3Rep3, 28SmS10P4Rep4, 33SmS18P5Rep1, 34SmS18P6Rep2, 35SmS18P7Rep3, 36SmS18P8Rep4 | Age and Stress |
| *S. macrospermum 10 d Control to 10 d water-stressed growth* | 21SmC10P1Rep1, 22SmC10P2Rep2, 23SmC10P3Rep3, 24SmC10P4Rep4, 25SmS10P1Rep1, 26SmS10P2Rep2, 27SmS10P3Rep3, 28SmS10P4Rep4 | Stress |
| *S. macrospermum 18 d Control to 18 d water-stressed growth* | 29SmC18P5Rep1, 30SmC18P6Rep2, 31SmC18P8Rep3, 32SmC18P9Rep4, 33SmS18P5Rep1, 34SmS18P6Rep2, 35SmS18P7Rep3, 36SmS18P8Rep4 | Stress |

| **Sample** | **RAW reads (M)** | **0.01 trimmed reads (M)** | **Single reads** | | | | **Paired end reads** | | | | |
| --- | --- | --- | --- | --- | --- | --- | --- | --- | --- | --- | --- |
|  |  |  | **Mapped (M)** | **Mapping %** | **Unmapped (M)** | **Unmapping %** | **Mapped in pairs (M)** | **Mapping %** | **Mapped in broken pairs (M)** | **Unmapped (M)** | **Unmapping %** |
| 01SbC10P1 | 125.73 | 122.11 | 0.68 | 34.71 | 1.27 | 65.29 | 42.24 | 35.15 | 2.38 | 75.54 | 62.86 |
| 02SbC10P2 | 119.11 | 115.79 | 0.59 | 33.93 | 1.14 | 66.07 | 38.93 | 34.23 | 2.23 | 72.90 | 63.92 |
| 03SbC10P3 | 120.48 | 116.92 | 0.66 | 34.01 | 1.29 | 65.99 | 39.53 | 34.38 | 2.29 | 73.14 | 63.62 |
| 04SbC10P4 | 120.67 | 117.35 | 0.61 | 35.13 | 1.13 | 64.87 | 40.86 | 35.34 | 2.19 | 72.56 | 62.77 |
| 05SbC10P5 | 147.98 | 143.9 | 0.79 | 34.40 | 1.51 | 65.60 | 48.28 | 34.10 | 2.78 | 90.54 | 63.94 |
| 06SbS10P1 | 111.66 | 108.76 | 0.58 | 36.69 | 1.00 | 63.31 | 39.23 | 36.60 | 2.08 | 65.87 | 61.45 |
| 07SbS10P2 | 129.17 | 125.65 | 0.65 | 34.98 | 1.21 | 65.02 | 43.40 | 35.06 | 2.50 | 77.90 | 62.92 |
| 08SbS10P3 | 121.26 | 117.65 | 0.76 | 37.81 | 1.25 | 62.19 | 43.58 | 37.69 | 2.36 | 69.69 | 60.27 |
| 09SbS10P4 | 136.73 | 132.5 | 0.87 | 34.91 | 1.63 | 65.09 | 44.18 | 33.98 | 2.78 | 83.04 | 63.88 |
| 10SbS10P5 | 115.72 | 112.49 | 0.64 | 38.17 | 1.04 | 61.83 | 42.66 | 38.50 | 2.15 | 65.99 | 59.55 |
| 11SbC47P6 | 135.57 | 131.88 | 0.77 | 37.78 | 1.27 | 62.22 | 48.09 | 37.04 | 2.61 | 79.15 | 60.95 |
| 12SbC47P7 | 128.54 | 124.96 | 0.76 | 38.05 | 1.23 | 61.95 | 45.62 | 37.10 | 2.46 | 74.89 | 60.90 |
| 13SbC47P8 | 123.10 | 119.63 | 0.75 | 38.74 | 1.19 | 61.26 | 44.22 | 37.58 | 2.43 | 71.02 | 60.35 |
| 14SbC47P9 | 115.75 | 112.31 | 0.70 | 37.74 | 1.15 | 62.26 | 41.81 | 37.85 | 2.26 | 66.39 | 60.10 |
| 15SbC47P10 | 121.98 | 118.34 | 0.76 | 37.74 | 1.25 | 62.26 | 44.39 | 38.16 | 2.35 | 69.60 | 59.82 |
| 16SbS47P6 | 117.14 | 113.99 | 0.68 | 38.36 | 1.09 | 61.64 | 43.05 | 38.36 | 2.34 | 66.84 | 59.55 |
| 17SbS47P7 | 125.92 | 122.43 | 0.71 | 37.34 | 1.20 | 62.66 | 44.61 | 37.01 | 2.45 | 73.46 | 60.95 |
| 18SbS47P8 | 116.54 | 113.36 | 0.68 | 38.64 | 1.08 | 61.36 | 42.82 | 38.37 | 2.27 | 66.51 | 59.60 |
| 19SbS47P9 | 135.66 | 132.20 | 0.73 | 38.45 | 1.18 | 61.55 | 49.85 | 38.26 | 2.67 | 77.77 | 59.69 |
| 20SbS47P10 | 109.64 | 106.73 | 0.66 | 40.28 | 0.98 | 59.72 | 41.35 | 39.34 | 2.07 | 61.68 | 58.68 |
| 21SmC10P1 | 130.24 | 126.53 | 0.75 | 35.38 | 1.37 | 64.62 | 42.25 | 33.96 | 4.40 | 77.77 | 62.51 |
| 22SmC10P2 | 121.73 | 118.11 | 0.83 | 39.72 | 1.26 | 60.28 | 43.50 | 37.50 | 4.52 | 68.00 | 58.61 |
| 23SmC10P3 | 119.46 | 115.79 | 0.75 | 36.47 | 1.31 | 63.53 | 39.96 | 35.14 | 4.25 | 69.51 | 61.12 |
| 24SmC10P4 | 124.02 | 120.61 | 0.74 | 38.70 | 1.17 | 61.30 | 43.79 | 36.89 | 4.39 | 70.52 | 59.41 |
| 25SmS10P1 | 126.02 | 122.56 | 0.70 | 37.31 | 1.18 | 62.69 | 44.03 | 36.49 | 4.59 | 72.06 | 59.71 |
| 26SmS10P2 | 112.75 | 109.36 | 0.84 | 43.17 | 1.11 | 56.83 | 44.44 | 41.37 | 4.37 | 58.60 | 54.56 |
| 27SmS10P3 | 121.10 | 117.78 | 0.70 | 38.06 | 1.14 | 61.94 | 42.56 | 36.70 | 4.43 | 68.95 | 59.47 |
| 28SmS10P4 | 105.31 | 102.30 | 0.75 | 44.44 | 0.94 | 55.56 | 42.90 | 42.64 | 4.09 | 53.62 | 53.29 |
| 29SmC18P5 | 130.81 | 126.93 | 0.72 | 33.55 | 1.42 | 66.45 | 41.14 | 32.97 | 4.41 | 79.24 | 63.50 |
| 30SmC18P6 | 113.04 | 109.61 | 0.80 | 40.20 | 1.18 | 59.80 | 40.76 | 37.87 | 4.12 | 62.74 | 58.30 |
| 31SmC18P8 | 128.74 | 125.08 | 0.74 | 35.90 | 1.32 | 64.10 | 42.12 | 34.24 | 4.33 | 76.58 | 62.24 |
| 32SmC18P9 | 115.12 | 111.44 | 0.75 | 36.54 | 1.31 | 63.46 | 38.87 | 35.54 | 3.96 | 66.55 | 60.84 |
| 33SmS18P5 | 109.94 | 106.97 | 0.69 | 41.19 | 0.98 | 58.81 | 42.45 | 40.31 | 4.00 | 58.86 | 55.90 |
| 34SmS18P6 | 110.52 | 107.34 | 0.67 | 37.18 | 1.14 | 62.82 | 37.73 | 35.75 | 3.84 | 63.96 | 60.61 |
| 35SmS18P7 | 128.64 | 124.66 | 0.81 | 34.74 | 1.53 | 65.26 | 40.28 | 32.93 | 4.29 | 77.75 | 63.56 |
| 36SmS18P8 | 113.45 | 110.16 | 0.75 | 40.21 | 1.11 | 59.79 | 41.81 | 38.60 | 4.20 | 62.30 | 57.52 |

**Table S2:** Details of single reads and paired end reads mapping in RNA sequencing

**Table S3:** Details of mapping percentages of one sample of *S. bicolor* in RNA sequencing

| **Method** |  | **Number of reads (paired + single + broken)** | **Percentage (%)** | **Percentage of the total reads (%)** |
| --- | --- | --- | --- | --- |
| **RNA Seq** | Total reads | 122,108,537 |  |  |
|  | Mapped | 45,299,445 | 37.10 | 37.10 |
|  | Unmapped | 76,809,092 | 62.90 | 62.90 |
| **Ribosomal RNA mapping** | Total reads | 76,809,092 |  | 62.90 |
|  | Mapped | 18,351,882 | 23.89 | 15.03 |
|  | Unmapped | 58,457,210 | 76.11 | 47.87 |
| **Long Noncoding RNA mapping** | Total reads | 58,457,210 |  | 47.87 |
|  | Mapped | 18,020,711 | 30.83 | 14.76 |
|  | Unmapped | 40,436,499 | 69.17 | 33.12 |
| **Total Genome mapping** | Total reads | 40,436,499 |  | 33.12 |
|  | Mapped | 40,111,249 | 99.20 | 32.84 |
|  | Unmapped | 325,250 | 0.80 | 0.24 |

**Table S4:** Details of BLAST annotation and coding potential analysis of the contigs of *S. bicolor*

| **BLAST Annotation** | |
| --- | --- |
| Total contigs | 62,283 |
| With BLAST (no hits) | 44,261 |
| With BLAST | 5,081 |
| With GO Mapping | 2,504 |
| With GO Annotation | 10,437 |
| **Coding Potential Analysis (ORF Type)** | |
| Complete | 15,864 |
| 3’ Partial | 3,754 |
| 5’ Partial | 10,215 |
| Internal | 1,221 |

**Table S5:** Description of the top 10 up- and down-regulated genes in different comparison groups of *S. bicolor*

| **Condition** | **Gene ID** | **Gene name** | **Up- or down-regulated** | **Fold Change** |
| --- | --- | --- | --- | --- |
| *S. bicolor 10 d Control to 47 d Control* | XR_002448965.1 | PREDICTED: uncharacterized LOC110431526 (LOC110431526), misc_RNA | Up | 7,106.04 |
|  | XM_021452698.1 | PREDICTED: protein HIRA (LOC8062538), transcript variant X1, mRNA | Up | 823.29 |
|  | XR_002447300.1 | PREDICTED: uncharacterized LOC110430086 (LOC110430086), transcript variant X6, ncRNA | Up | 794.51 |
|  | XR_002446826.1 | PREDICTED: uncharacterized LOC110429724 (LOC110429724), transcript variant X1, ncRNA | Up | 664.72 |
|  | XM_021460625.1 | PREDICTED: glutamyl-tRNA (Gln) amidotransferase subunit A (LOC8064826), transcript variant X2, mRNA | Up | 636.32 |
|  | XM_021451463.1 | PREDICTED: uncharacterized LOC8080432 (LOC8080432), transcript variant X5, mRNA | Up | 604.4 |
|  | XM_021463065.1 | PREDICTED: anthocyanin regulatory R-S protein (LOC8056591), transcript variant X2, mRNA | Up | 594.41 |
|  | XM_021462672.1 | PREDICTED: actin-related protein 9 (LOC8067782), transcript variant X2, mRNA | Up | 549.27 |
|  | XR_002452324.1 | PREDICTED: uncharacterized LOC110434684 (LOC110434684), transcript variant X6, ncRNA | Up | 519.38 |
|  | XM_002455302.2 | PREDICTED: probable 3-beta-hydroxysteroid-Delta (8), Delta (7)-isomerase (LOC8085052), transcript variant X2, mRNA | Up | 491.24 |
|  | XM_021448766.1 | PREDICTED: uncharacterized LOC8065041 (LOC8065041), transcript variant X4, mRNA | Down | -2516.60 |
|  | XM_002464363.2 | PREDICTED: non-specific lipid-transfer protein 2P (LOC8067073), mRNA | Down | -2114.90 |
|  | XM_002455580.2 | PREDICTED: glutaredoxin-C1 (LOC8086360), mRNA | Down | -1576.49 |
|  | XM_021451126.1 | PREDICTED: uncharacterized WD repeat-containing protein C2A9.03 (LOC8080108), transcript variant X4, mRNA | Down | -1244.88 |
|  | XM_002455581.2 | PREDICTED: glutaredoxin-C1 (LOC8086361), mRNA | Down | -785.80 |
|  | XM_021455104.1 | PREDICTED: probable LRR receptor-like serine/threonine-protein kinase At1g06840 (LOC8055302), transcript variant X2, mRNA | Down | -780.15 |
|  | XM_021458295.1 | PREDICTED: protein PHYLLO, chloroplastic (LOC8055409), transcript variant X5, mRNA | Down | -743.61 |
|  | XR_002454959.1 | PREDICTED: protein FAR1-RELATED SEQUENCE 5 (LOC8080870), transcript variant X10, misc_RNA | Down | -731.01 |
|  | XM_021448765.1 | PREDICTED: uncharacterized LOC8065041 (LOC8065041), transcript variant X3, mRNA | Down | -676.35 |
|  | XM_021449964.1 | PREDICTED: uncharacterized LOC110431213 (LOC110431213), transcript variant X6, mRNA | Down | -671.13 |
| *S. bicolor 10 d water-stressed to 47 d water-stressed growth* | XM_021446394.1 | PREDICTED: scarecrow-like protein 9 (LOC110429790), transcript variant X3, mRNA | Up | 1,179.42 |
|  | XM_002438473.2 | PREDICTED: phospholipase A1-II 7 (LOC8072679), mRNA | Up | 1,223.29 |
|  | XM_021446682.1 | PREDICTED: pyridoxal kinase (LOC110429921), transcript variant X1, mRNA | Up | 1,006.56 |
|  | XM_021449640.1 | PREDICTED: uncharacterized LOC110431053 (LOC110431053), transcript variant X5, mRNA | Up | 809.98 |
|  | XM_021452920.1 | PREDICTED: putative disease resistance protein RGA3 (LOC8067274), transcript variant X5, mRNA | Up | 608.81 |
|  | XM_021461625.1 | PREDICTED: uncharacterized LOC8076785 (LOC8076785), transcript variant X4, mRNA | Up | 521.95 |
|  | XM_021464869.1 | PREDICTED: PP2A regulatory subunit TAP46 (LOC8060578), transcript variant X4, mRNA | Up | 512.48 |
|  | XR_002447298.1 | PREDICTED: uncharacterized LOC110430086 (LOC110430086), transcript variant X4, ncRNA | Up | 512.33 |
|  | XR_002447436.1 | PREDICTED: uncharacterized LOC110430154 (LOC110430154), transcript variant X1, ncRNA | Up | 491.33 |
|  | XR_002452972.1 | PREDICTED: uncharacterized LOC110435328 (LOC110435328), transcript variant X4, ncRNA | Up | 486.09 |
|  | XM_021454267.1 | PREDICTED: uncharacterized LOC8079218 (LOC8079218), transcript variant X6, mRNA | Down | -1,667.50 |
|  | XM_021446848.1 | PREDICTED: transcription elongation factor SPT6-like (LOC8077045), transcript variant X4, mRNA | Down | -1,414.58 |
|  | XM_021455104.1 | PREDICTED: probable LRR receptor-like serine/threonine-protein kinase At1g06840 (LOC8055302), transcript variant X2, mRNA | Down | -1,278.08 |
|  | XM_021454480.1 | PREDICTED: uncharacterized LOC110433006 (LOC110433006), transcript variant X1, mRNA | Down | -850.81 |
|  | XR_002450362.1 | PREDICTED: uncharacterized LOC110433006 (LOC110433006), transcript variant X5, misc_RNA | Down | -792.51 |
|  | XR_002446633.1 | PREDICTED: uncharacterized LOC110429557 (LOC110429557), transcript variant X3, ncRNA | Down | -628.92 |
|  | XM_021445862.1 | PREDICTED: sulfate transporter 1.2 (LOC8081890), transcript variant X3, mRNA | Down | -614.96 |
|  | XM_021462428.1 | PREDICTED: alpha carbonic anhydrase 1, chloroplastic (LOC8075274), transcript variant X3, mRNA | Down | -537.98 |
|  | XR_002448003.1 | PREDICTED: uncharacterized LOC110430623 (LOC110430623), transcript variant X3, ncRNA | Down | -511.06 |
|  | XR_002451008.1 | PREDICTED: uncharacterized LOC110433537 (LOC110433537), transcript variant X12, ncRNA | Down | -499.42 |
| *S. bicolor 10 d Control to 10 d water-stressed growth* | XM_002438289.2 | PREDICTED: protein LE25 (LOC8078263), mRNA | Up | 2,136.05 |
|  | XM_021452698.1 | PREDICTED: protein HIRA (LOC8062538), transcript variant X1, mRNA | Up | 1,900.95 |
|  | XR_002448965.1 | PREDICTED: uncharacterized LOC110431526 (LOC110431526), misc_RNA | Up | 1,413.90 |
|  | XR_002452096.1 | PREDICTED: probable starch synthase 4, chloroplastic/amyloplastic (LOC8056415), transcript variant X7, misc_RNA | Up | 990.47 |
|  | XR_002452106.1 | PREDICTED: uncharacterized LOC110434577 (LOC110434577), transcript variant X5, ncRNA | Up | 888.95 |
|  | XR_002446826.1 | PREDICTED: uncharacterized LOC110429724 (LOC110429724), transcript variant X1, ncRNA | Up | 731.06 |
|  | XM_021458006.1 | PREDICTED: uncharacterized LOC110434200 (LOC110434200), transcript variant X3, mRNA | Up | 702.8 |
|  | XM_021451463.1 | PREDICTED: uncharacterized LOC8080432 (LOC8080432), transcript variant X5, mRNA | Up | 578.42 |
|  | XR_002449628.1 | PREDICTED: serine/threonine-protein kinase EDR1 (LOC8059428), transcript variant X5, misc_RNA | Up | 510.03 |
|  | XR_002448509.1 | PREDICTED: uncharacterized LOC110431026 (LOC110431026), transcript variant X18, ncRNA | Up | 493.48 |
|  | XM_021448766.1 | PREDICTED: uncharacterized LOC8065041 (LOC8065041), transcript variant X4, mRNA | Down | -2,286.32 |
|  | XM_021447262.1 | PREDICTED: putative disease resistance protein RGA3 (LOC8061311), transcript variant X4, mRNA | Down | -1,024.24 |
|  | XM_002462990.2 | PREDICTED: putative cell wall protein (LOC8056345), mRNA | Down | -973.94 |
|  | XM_002456977.2 | PREDICTED: cysteine protease XCP2 (LOC8074134), mRNA | Down | -701.24 |
|  | XM_021463899.1 | PREDICTED: putative disease resistance RPP13-like protein 1 (LOC8070959), transcript variant X5, mRNA | Down | -699.05 |
|  | XM_021462053.1 | PREDICTED: probable disease resistance protein RF9 (LOC8085995), transcript variant X3, mRNA | Down | -684.16 |
|  | XM_021446394.1 | PREDICTED: scarecrow-like protein 9 (LOC110429790), transcript variant X3, mRNA | Down | -603.22 |
|  | XM_021454636.1 | PREDICTED: putative wall-associated receptor kinase-like 16 (LOC110433045), transcript variant X2, mRNA | Down | -580.89 |
|  | XM_021452102.1 | PREDICTED: cysteine-rich receptor-like protein kinase 10 (LOC8084085), transcript variant X7, mRNA | Down | -580.53 |
|  | XM_021449296.1 | PREDICTED: disease resistance protein RPM1-like (LOC110430973), transcript variant X2, mRNA | Down | -499.67 |
| *S. bicolor 47 d Control to 47 d water-stressed growth* | XM_021451126.1 | PREDICTED: uncharacterized WD repeat-containing protein C2A9.03 (LOC8080108), transcript variant X4, mRNA | Up | 13,771.30 |
|  | XM_002438289.2 | PREDICTED: protein LE25 (LOC8078263), mRNA | Up | 1,295.42 |
|  | XM_002459721.2 | PREDICTED: uncharacterized LOC8059722 (LOC8059722), mRNA | Up | 1,167.21 |
|  | XM_021453777.1 | PREDICTED: uncharacterized LOC8059008 (LOC8059008), transcript variant X1, mRNA | Up | 1,146.36 |
|  | XM_021447547.1 | PREDICTED: late embryogenesis abundant protein, group 3-like (LOC110430243), mRNA | Up | 1,019.21 |
|  | XM_021462156.1 | PREDICTED: DNA-binding protein EMBP-1 (LOC8057096), transcript variant X1, mRNA | Up | 843.28 |
|  | XM_021456729.1 | PREDICTED: auxilin-like protein 1 (LOC8058204), transcript variant X2, mRNA | Up | 774.14 |
|  | XM_002444176.2 | PREDICTED: late embryogenesis abundant protein D-34 (LOC8069889), mRNA | Up | 692.92 |
|  | XR_002447436.1 | PREDICTED: uncharacterized LOC110430154 (LOC110430154), transcript variant X1, ncRNA | Up | 560.45 |
|  | XR_002454330.1 | PREDICTED: uncharacterized LOC110436550 (LOC110436550), transcript variant X7, ncRNA | Up | 526.31 |
|  | XM_021454538.1 | PREDICTED: ent-copalyl diphosphate synthase 1, chloroplastic (LOC8064919), transcript variant X3, mRNA | Down | -1,185.05 |
|  | XR_002450362.1 | PREDICTED: uncharacterized LOC110433006 (LOC110433006), transcript variant X5, misc_RNA | Down | -1,161.70 |
|  | XM_021465335.1 | PREDICTED: achilleol B synthase (LOC8073228), transcript variant X1, mRNA | Down | -1,113.28 |
|  | XM_021460952.1 | PREDICTED: cysteine-rich receptor-like protein kinase 6 (LOC110435424), transcript variant X2, mRNA | Down | -1,076.35 |
|  | XR_002446769.1 | PREDICTED: uncharacterized LOC110429661 (LOC110429661), transcript variant X2, ncRNA | Down | -722.78 |
|  | XR_002451373.1 | PREDICTED: uncharacterized LOC110433932 (LOC110433932), transcript variant X14, misc_RNA | Down | -654.61 |
|  | XM_021462237.1 | PREDICTED: uncharacterized LOC8077088 (LOC8077088), transcript variant X1, mRNA | Down | -615.45 |
|  | XR_002448003.1 | PREDICTED: uncharacterized LOC110430623 (LOC110430623), transcript variant X3, ncRNA | Down | -613.69 |
|  | XR_002447096.1 | PREDICTED: protein FAR1-RELATED SEQUENCE 6 (LOC8060896), transcript variant X4, misc_RNA | Down | -607.29 |
|  | XR_002448468.1 | PREDICTED: uncharacterized LOC8065497 (LOC8065497), transcript variant X3, misc_RNA | Down | -571.16 |

**Table S6:** Description of the top 10 up- and down-regulated genes in different comparison groups of *S. macrospermum*

| **Condition** | **Gene ID** | **Gene name** | **Up or down regulated** | **Fold Change** |
| --- | --- | --- | --- | --- |
| *S. macrospermum 10 d Control to 18 d Control* | XM_021446732.1 | PREDICTED: probable E3 ubiquitin-protein ligase HIP1 (LOC8071210), transcript variant X1, mRNA | Up | 1,367.54 |
|  | XM_002457500.2 | PREDICTED: serine/arginine-rich splicing factor 11 (LOC8072378), transcript variant X1, mRNA | Up | 977.18 |
|  | XM_021459764.1 | PREDICTED: protein photoperiod-independent early flowering 1 (LOC8073897), transcript variant X8, mRNA | Up | 970.87 |
|  | XM_021460047.1 | PREDICTED: coleoptile phototropism protein 1 (LOC8068928), transcript variant X2, mRNA | Up | 827.70 |
|  | XM_021465363.1 | PREDICTED: uncharacterized LOC8060583 (LOC8060583), transcript variant X2, mRNA | Up | 164.56 |
|  | XM_021448002.1 | PREDICTED: probable ethylene response sensor 2 (LOC8076011), transcript variant X2, mRNA | Up | 137.60 |
|  | XM_021446860.1 | PREDICTED: uncharacterized LOC8077664 (LOC8077664), transcript variant X2, mRNA | Up | 130.24 |
|  | XM_002457184.2 | PREDICTED: U3 small nucleolar RNA-associated protein 18 homolog (LOC8078766), transcript variant X3, mRNA | Up | 123.53 |
|  | XM_021448319.1 | PREDICTED: phosphoribosylamine-glycine ligase (LOC8068993), transcript variant X3, mRNA | Up | 116.05 |
|  | XR_002448937.1 | PREDICTED: uncharacterized LOC110431474 (LOC110431474), misc_RNA | Up | 82.93 |
|  | XM_021448545.1 | PREDICTED: chromatin structure-remodeling complex protein SYD (LOC8061515), transcript variant X5, mRNA | Down | -3,332.58 |
|  | XM_021448547.1 | PREDICTED: chromatin structure-remodeling complex protein SYD (LOC8061515), transcript variant X6, mRNA | Down | -1,870.35 |
|  | XM_002440559.2 | PREDICTED: hemolysin A (LOC8069504), transcript variant X1, mRNA | Down | -1,274.62 |
|  | XM_021460041.1 | PREDICTED: oligoribonuclease (LOC8077814), transcript variant X2, mRNA | Down | -896.70 |
|  | XM_021452939.1 | PREDICTED: histone-lysine N-methyltransferase TRX1 (LOC8062009), transcript variant X3, mRNA | Down | -845.79 |
|  | XR_002454170.1 | PREDICTED: eIF-2-alpha kinase GCN2 (LOC8073711), transcript variant X4, misc_RNA | Down | -833.74 |
|  | XM_021450683.1 | PREDICTED: uncharacterized LOC110431557 (LOC110431557), transcript variant X5, mRNA | Down | -713.14 |
|  | XM_021452920.1 | PREDICTED: putative disease resistance protein RGA3 (LOC8067274), transcript variant X5, mRNA | Down | -219.39 |
|  | XM_021447357.1 | PREDICTED: uncharacterized LOC8061061 (LOC8061061), mRNA | Down | -178.68 |
|  | XM_021462839.1 | PREDICTED: G-type lectin S-receptor-like serine/threonine-protein kinase At2g19130 (LOC8073019), mRNA | Down | -135.85 |
| *S. macrospermum 10 d water- stressed to 18 d water-stressed growth* | XR_002448965.1 | PREDICTED: uncharacterized LOC110431526 (LOC110431526), misc_RNA | Up | 6,020.84 |
|  | XM_021453798.1 | PREDICTED: nucleolar GTP-binding protein 1 (LOC8074422), transcript variant X6, mRNA | Up | 2,440.05 |
|  | XM_002436739.2 | PREDICTED: anthranilate O-methyltransferase 2 (LOC8065709), transcript variant X1, mRNA | Up | 2,314.67 |
|  | XM_002438095.2 | PREDICTED: U3 small nucleolar ribonucleoprotein protein IMP3 (LOC8061490), transcript variant X2, mRNA | Up | 2,123.88 |
|  | XM_021453655.1 | PREDICTED: uncharacterized LOC8080981 (LOC8080981), transcript variant X2, mRNA | Up | 1,706.25 |
|  | XM_021446043.1 | PREDICTED: achilleol B synthase (LOC8074270), transcript variant X2, mRNA | Up | 1,293.68 |
|  | XR_002449848.1 | PREDICTED: uncharacterized LOC110432399 (LOC110432399), transcript variant X4, misc_RNA | Up | 967.46 |
|  | XR_002446647.1 | PREDICTED: uncharacterized protein At1g04910 (LOC8079887), transcript variant X2, misc_RNA | Up | 942.46 |
|  | XM_002440317.2 | PREDICTED: RNA polymerase sigma factor SigA1 (LOC8066548), transcript variant X2, mRNA | Up | 821.13 |
|  | XM_002453039.2 | PREDICTED: uncharacterized LOC8084448 (LOC8084448), transcript variant X1, mRNA | Up | 766.09 |
|  | XM_021464920.1 | PREDICTED: probable gamma-aminobutyrate transaminase 4 (LOC8076546), mRNA | Down | -13,600.53 |
|  | XM_021460055.1 | PREDICTED: cytochrome b5 (LOC8075154), transcript variant X2, mRNA | Down | -3,966.22 |
|  | XR_002451789.1 | PREDICTED: uncharacterized LOC110434355 (LOC110434355), transcript variant X10, ncRNA | Down | -1,639.01 |
|  | XM_021451570.1 | PREDICTED: probable transcription factor At3g04930 (LOC8063399), transcript variant X2, mRNA | Down | -1,287.04 |
|  | XM_002467370.2 | PREDICTED: probable methyltransferase PMT21 (LOC8086276), transcript variant X1, mRNA | Down | -1,212.09 |
|  | XR_002446634.1 | PREDICTED: putative disease resistance RPP13-like protein 3 (LOC8067150), transcript variant X2, misc_RNA | Down | -1,169.97 |
|  | XM_021459204.1 | PREDICTED: thiosulfate/3-mercaptopyruvate sulfurtransferase 1, mitochondrial (LOC8074692), transcript variant X2, mRNA | Down | -1,048.13 |
|  | XM_021448547.1 | PREDICTED: chromatin structure-remodeling complex protein SYD (LOC8061515), transcript variant X6, mRNA | Down | -934.68 |
|  | XR_002450838.1 | PREDICTED: piezo-type mechanosensitive ion channel homolog (LOC8069573), transcript variant X3, misc_RNA | Down | -918.16 |
|  | XM_021455319.1 | PREDICTED: endoplasmic reticulum oxidoreductin-1 (LOC8085791), transcript variant X1, mRNA | Down | -860.73 |
| *S. macrospermum 10 d Control to 10 d water-stressed growth* | XM_021464920.1 | *PREDICTED: probable gamma-aminobutyrate transaminase 4 (LOC8076546), mRNA* | Up | 11,252.95 |
|  | XM_021459660.1 | PREDICTED: RNA-binding protein cabeza (LOC110434870), transcript variant X1, mRNA | Up | 5,463.37 |
|  | XM_021445682.1 | PREDICTED: uncharacterized LOC8082661 (LOC8082661), transcript variant X6, mRNA | Up | 5,030.39 |
|  | XM_021448835.1 | PREDICTED: probable protein phosphatase 2C 59 (LOC110430818), transcript variant X2, mRNA | Up | 3,957.88 |
|  | XM_021460055.1 | PREDICTED: cytochrome b5 (LOC8075154), transcript variant X2, mRNA | Up | 3,329.75 |
|  | XM_002457500.2 | PREDICTED: serine/arginine-rich splicing factor 11 (LOC8072378), transcript variant X1, mRNA | Up | 3,033.69 |
|  | XM_021464354.1 | PREDICTED: alcohol dehydrogenase 1 (LOC110436814), transcript variant X3, mRNA | Up | 2,356.08 |
|  | XM_021455657.1 | PREDICTED: CTP synthase-like (LOC110433469), transcript variant X2, mRNA | Up | 2,313.72 |
|  | XM_021447547.1 | PREDICTED: late embryogenesis abundant protein, group 3-like (LOC110430243), mRNA | Up | 2,150.75 |
|  | XM_002446459.2 | PREDICTED: seed biotin-containing protein SBP65 (LOC8080308), mRNA | Up | 1,578.90 |
|  | XM_021459666.1 | PREDICTED: RNA-binding protein cabeza (LOC110434870), transcript variant X7, mRNA | Down | -4,493.29 |
|  | XM_002438095.2 | PREDICTED: U3 small nucleolar ribonucleoprotein protein IMP3 (LOC8061490), transcript variant X2, mRNA | Down | -2,713.47 |
|  | XM_002458326.2 | PREDICTED: uncharacterized LOC8059152 (LOC8059152), mRNA | Down | -1,886.63 |
|  | XR_002449848.1 | PREDICTED: uncharacterized LOC110432399 (LOC110432399), transcript variant X4, misc_RNA | Down | -1,774.48 |
|  | XM_021454830.1 | PREDICTED: cysteine-rich receptor-like protein kinase 10 (LOC8081707), transcript variant X3, mRNA | Down | -1,589.41 |
|  | XM_021448659.1 | PREDICTED: uncharacterized LOC8155683 (LOC8155683), transcript variant X5, mRNA | Down | -1,576.52 |
|  | XM_002440559.2 | PREDICTED: hemolysin A (LOC8069504), transcript variant X1, mRNA | Down | -1,544.86 |
|  | XM_021462839.1 | PREDICTED: G-type lectin S-receptor-like serine/threonine-protein kinase At2g19130 (LOC8073019), mRNA | Down | -1,354.50 |
|  | XM_021447136.1 | PREDICTED: abscisic acid 8'-hydroxylase 4 (LOC8066580), transcript variant X1, mRNA | Down | -1,208.97 |
|  | XM_021453655.1 | PREDICTED: uncharacterized LOC8080981 (LOC8080981), transcript variant X2, mRNA | Down | -1,139.65 |
| *S. macrospermum 18 d Control to 18 d water-stressed growth* | XM_021459660.1 | PREDICTED: RNA-binding protein cabeza (LOC110434870), transcript variant X1, mRNA | Up | 6,284.51 |
|  | XR_002448965.1 | PREDICTED: uncharacterized LOC110431526 (LOC110431526), misc_RNA | Up | 4,983.52 |
|  | XM_021456802.1 | PREDICTED: homeobox-DDT domain protein RLT2 (LOC8061841), transcript variant X3, mRNA | Up | 4,669.82 |
|  | XM_021452670.1 | PREDICTED: uncharacterized LOC8074192 (LOC8074192), transcript variant X3, mRNA | Up | 2,550.57 |
|  | XM_002452709.2 | PREDICTED: oil body-associated protein 1A (LOC8073213), mRNA | Up | 2,395.05 |
|  | XM_021456563.1 | PREDICTED: protein LNK2 (LOC8074905), transcript variant X4, mRNA | Up | 2,220.49 |
|  | XM_021464354.1 | PREDICTED: alcohol dehydrogenase 1 (LOC110436814), transcript variant X3, mRNA | Up | 1,848.17 |
|  | XM_021448992.1 | PREDICTED: HEAT repeat-containing protein 5B (LOC8058820), transcript variant X3, mRNA | Up | 1,398.99 |
|  | XM_021459025.1 | PREDICTED: peptidyl-prolyl cis-trans isomerase CYP95 (LOC110434624), transcript variant X3, mRNA | Up | 1,326.31 |
|  | XM_021458858.1 | PREDICTED: uncharacterized LOC8068922 (LOC8068922), transcript variant X8, mRNA | Up | 1,304.60 |
|  | XM_021450534.1 | PREDICTED: uncharacterized RNA-binding protein C17H9.04c (LOC8068430), transcript variant X3, mRNA | Down | -5,133.26 |
|  | XM_021459666.1 | PREDICTED: RNA-binding protein cabeza (LOC110434870), transcript variant X7, mRNA | Down | -3,274.20 |
|  | XM_002448494.2 | PREDICTED: germin-like protein 4-1 (LOC8066791), mRNA | Down | -1,964.41 |
|  | XM_021453747.1 | PREDICTED: protein FAR1-RELATED SEQUENCE 9 (LOC8080743), transcript variant X1, mRNA | Down | -1,941.45 |
|  | XM_002465356.2 | PREDICTED: uncharacterized LOC8081111 (LOC8081111), transcript variant X2, mRNA | Down | -1,847.10 |
|  | XR_002451360.1 | PREDICTED: uncharacterized LOC8057729 (LOC8057729), transcript variant X4, misc_RNA | Down | -1,777.90 |
|  | XM_021463473.1 | PREDICTED: uncharacterized LOC8072321 (LOC8072321), transcript variant X5, mRNA | Down | -1,385.85 |
|  | XM_021446561.1 | PREDICTED: transcription factor PIF4 (LOC8070136), transcript variant X4, mRNA | Down | -1,201.07 |
|  | XM_021460426.1 | PREDICTED: protein STRUBBELIG-RECEPTOR FAMILY 7 (LOC8077089), transcript variant X2, mRNA | Down | -1,100.66 |
|  | XM_021461081.1 | PREDICTED: uncharacterized LOC110435491 (LOC110435491), transcript variant X3, mRNA | Down | -1,015.94 |

**Table S7:** Description of the common genes which were differentially expressed in all the comparison groups

| **Comparison group** | **Up-/Down-regulated** | **Common gene ID** | **Description** | **Function** |
| --- | --- | --- | --- | --- |
| *S. bicolor 10 d Control to 47 d Control,  S. bicolor 10 d water-stressed to 47 d water-stressed growth, S. bicolor 10 d Control to 10 d water-stressed growth and S. bicolor 47 d Control to 47 d water-stressed growth* | Up | XM_021462260.1 | PREDICTED: homeobox-leucine zipper protein ROC4 (LOC8057552), mRNA | Involves in transcription and transcription regulation (DNA binding and lipid binding) |
|  |  | XM_021451123.1 | PREDICTED: uncharacterized WD repeat-containing protein C2A9.03 (LOC8080108), transcript variant X1, mRNA | protein binding |
|  |  | XM_021451124.1 | PREDICTED: uncharacterized WD repeat-containing protein C2A9.03 (LOC8080108), transcript variant X2, mRNA | protein binding |
|  |  | XM_021451127.1 | PREDICTED: uncharacterized WD repeat-containing protein C2A9.03 (LOC8080108), transcript variant X5, mRNA | protein binding |
|  |  | XM_021451128.1 | PREDICTED: uncharacterized WD repeat-containing protein C2A9.03 (LOC8080108), transcript variant X6, mRNA | protein binding |
|  |  | XM_002441266.2 | PREDICTED: EID1-like F-box protein 3 (LOC8064182), mRNA | protein binding |
|  |  | XM_021447178.1 | PREDICTED: (S)-beta-macrocarpene synthase (LOC8067303), transcript variant X1, mRNA | protein binding |
|  |  | XM_002466708.2 | PREDICTED: 9-cis-epoxycarotenoid dioxygenase 1, chloroplastic (LOC8062208), mRNA | Involves in carotene catabolic process (carotenoid dioxygenase activity and metal ion binding) |
|  |  | XM_002466192.2 | PREDICTED: probable galactinol--sucrose galactosyltransferase 2 (LOC8057100), mRNA | Involves in carbohydrate metabolic process (raffinose alpha-galactosidase activity) |
|  |  | XM_021447906.1 | PREDICTED: probable protein phosphatase 2C 49 (LOC110430337), mRNA | metal ion binding, protein serine phosphatase activity and protein threonine phosphatase activity |
| *S. bicolor 10 d Control to 47 d Control,*  *S. bicolor 10 d water-stressed to 47 d water-stressed growth,*  *S. bicolor 10 d Control to 10 d water-stressed growth and*  *S. bicolor 47 d Control to 47 d water-stressed growth* | Down | XM_002458701.2 | PREDICTED: laccase-4 (LOC8060080), mRNA | Involves in lignin catabolic process (copper ion binding, hydroquinone: oxygen oxidoreductase activity and oxidoreductase activity) |
|  |  | XM_002439869.2 | PREDICTED: flavonoid 3'-monooxygenase (LOC8072541), mRNA | heme binding, iron ion binding, monooxygenase activity and oxidoreductase activity |
| *S. macrospermum 10 d Control to 18 d Control, S. macrospermum 10 d water- stressed to 18 d water-stressed growth, S. macrospermum 10 d Control to 10 d water-stressed growth,* and *S. macrospermum 18 d Control to 18 d water-stressed growth* | Up | none | none |  |
| *S. macrospermum 10 d Control to 18 d Control, S. macrospermum 10 d water- stressed to 18 d water-stressed growth, S. macrospermum 10 d Control to 10 d water-stressed growth, and S. macrospermum 18 d Control to 18 d water-stressed growth* | Down | XM_002454531.2 | PREDICTED: salutaridine reductase (LOC8078894), mRNA | oxidoreductase activity |

**Table S8:** Expression values and the transcripts per million of the cyanogenesis related genes for the two species under all the conditions

| **Sample** | **Name** | **Gene** | **Expression value** | **Transcripts Per Million** |
| --- | --- | --- | --- | --- |
| 01SbC10P1Rep1 | XM_002466054.2 | tyrosine N-monooxygenase - *CYP79A1* | 17289.00 | 222.70 |
|  | XM_002466052.2 | 4-hydroxyphenylacetaldehyde oxime monooxygenase-like - *CYP71E1* | 8594.00 | 111.48 |
|  | XM_002463473.2 | cyanohydrin beta-glucosyltransferase – *UGT85B1* | 3370.00 | 51.43 |
|  | XM_002441984.2 | 4-hydroxy-7-methoxy-3-oxo-3,4-dihydro-2H-1,4-benzoxazin-2-yl glucoside beta-D-glucosidase 2, chloroplastic - Dhurrinase 1 | 106.00 | 1.44 |
|  | XM_002443028.2 | 4-hydroxy-7-methoxy-3-oxo-3,4-dihydro-2H-1,4-benzoxazin-2-yl glucoside beta-D-glucosidase 2, chloroplastic - Dhurrinase 2 | 7715.00 | 113.16 |
|  | XM_021446455.1 | Dhurrinase –like 3 | 12.00 | 0.61 |
|  | XM_021460447.1 | P-(S)-hydroxymandelonitrile lyase - *HNL* | 16916.00 | 237.88 |
|  | XM_002447428.2 | bifunctional L-3-cyanoalanine synthase/cysteine synthase 2, mitochondrial - *CAS C1* | 14350.00 | 241.27 |
|  | XM_002452453.2 | bifunctional nitrilase/nitrile hydratase - *NIT4A* | 3286.00 | 55.72 |
|  | XM_021459324.1 | bifunctional nitrilase/nitrile hydratase - *NIT4B2* | 30.00 | 0.45 |
|  | XM_002447182.2 | probable isoaspartyl peptidase/L-asparaginase 2 | 1116.00 | 21.88 |
|  | XM_002464220.2 | isoaspartyl peptidase/L-asparaginase 1 | 96.00 | 1.69 |
|  | XM_021447365.1 | *SbMATE2* | 1951.00 | 26.71 |
|  | XM_002464023.2 | *SbCGTR1* | 2491.00 | 30.56 |
| 02SbC10P2Rep2 | XM_002466054.2 | tyrosine N-monooxygenase - *CYP79A1* | 11766.00 | 158.58 |
|  | XM_002466052.2 | 4-hydroxyphenylacetaldehyde oxime monooxygenase-like - *CYP71E1* | 5919.00 | 80.34 |
|  | XM_002463473.2 | cyanohydrin beta-glucosyltransferase – *UGT85B1* | 2398.00 | 38.29 |
|  | XM_002441984.2 | 4-hydroxy-7-methoxy-3-oxo-3,4-dihydro-2H-1,4-benzoxazin-2-yl glucoside beta-D-glucosidase 2, chloroplastic - Dhurrinase 1 | 48.00 | 0.68 |
|  | XM_002443028.2 | 4-hydroxy-7-methoxy-3-oxo-3,4-dihydro-2H-1,4-benzoxazin-2-yl glucoside beta-D-glucosidase 2, chloroplastic - Dhurrinase 2 | 9105.00 | 139.74 |
|  | XM_021446455.1 | Dhurrinase –like 3 | 6.00 | 0.32 |
|  | XM_021460447.1 | P-(S)-hydroxymandelonitrile lyase - *HNL* | 12940.00 | 190.40 |
|  | XM_002447428.2 | bifunctional L-3-cyanoalanine synthase/cysteine synthase 2, mitochondrial - *CAS C1* | 15393.00 | 270.80 |
|  | XM_002452453.2 | bifunctional nitrilase/nitrile hydratase - *NIT4A* | 2967.00 | 52.65 |
|  | XM_021459324.1 | bifunctional nitrilase/nitrile hydratase - *NIT4B2* | 35.00 | 0.55 |
|  | XM_002447182.2 | probable isoaspartyl peptidase/L-asparaginase 2 | 1134.00 | 23.27 |
|  | XM_002464220.2 | isoaspartyl peptidase/L-asparaginase 1 | 76.00 | 1.40 |
|  | XM_021447365.1 | *SbMATE2* | 1415.00 | 20.27 |
|  | XM_002464023.2 | *SbCGTR1* | 631.00 | 8.10 |
| 03SbC10P3Rep3 | XM_002466054.2 | tyrosine N-monooxygenase - *CYP79A1* | 2161.00 | 28.81 |
|  | XM_002466052.2 | 4-hydroxyphenylacetaldehyde oxime monooxygenase-like - *CYP71E1* | 1426.00 | 19.15 |
|  | XM_002463473.2 | cyanohydrin beta-glucosyltransferase – *UGT85B1* | 737.00 | 11.64 |
|  | XM_002441984.2 | 4-hydroxy-7-methoxy-3-oxo-3,4-dihydro-2H-1,4-benzoxazin-2-yl glucoside beta-D-glucosidase 2, chloroplastic - Dhurrinase 1 | 11.00 | 0.16 |
|  | XM_002443028.2 | 4-hydroxy-7-methoxy-3-oxo-3,4-dihydro-2H-1,4-benzoxazin-2-yl glucoside beta-D-glucosidase 2, chloroplastic - Dhurrinase 2 | 4663.00 | 70.79 |
|  | XM_021446455.1 | Dhurrinase –like 3 | 1.00 | 0.05 |
|  | XM_021460447.1 | P-(S)-hydroxymandelonitrile lyase - *HNL* | 9717.00 | 141.43 |
|  | XM_002447428.2 | bifunctional L-3-cyanoalanine synthase/cysteine synthase 2, mitochondrial - *CAS C1* | 16626.00 | 289.33 |
|  | XM_002452453.2 | bifunctional nitrilase/nitrile hydratase - *NIT4A* | 4713.00 | 82.72 |
|  | XM_021459324.1 | bifunctional nitrilase/nitrile hydratase - *NIT4B2* | 88.00 | 1.37 |
|  | XM_002447182.2 | probable isoaspartyl peptidase/L-asparaginase 2 | 1502.00 | 30.49 |
|  | XM_002464220.2 | isoaspartyl peptidase/L-asparaginase 1 | 34.00 | 0.62 |
|  | XM_021447365.1 | *SbMATE2* | 685.00 | 9.71 |
|  | XM_002464023.2 | *SbCGTR1* | 760.00 | 9.65 |
| 04SbC10P4Rep4 | XM_002466054.2 | tyrosine N-monooxygenase - *CYP79A1* | 6020.00 | 80.00 |
|  | XM_002466052.2 | 4-hydroxyphenylacetaldehyde oxime monooxygenase-like - *CYP71E1* | 3758.00 | 50.29 |
|  | XM_002463473.2 | cyanohydrin beta-glucosyltransferase – *UGT85B1* | 1689.00 | 26.59 |
|  | XM_002441984.2 | 4-hydroxy-7-methoxy-3-oxo-3,4-dihydro-2H-1,4-benzoxazin-2-yl glucoside beta-D-glucosidase 2, chloroplastic - Dhurrinase 1 | 56.00 | 0.79 |
|  | XM_002443028.2 | 4-hydroxy-7-methoxy-3-oxo-3,4-dihydro-2H-1,4-benzoxazin-2-yl glucoside beta-D-glucosidase 2, chloroplastic - Dhurrinase 2 | 2593.00 | 39.24 |
|  | XM_021446455.1 | Dhurrinase –like 3 | 6.00 | 0.31 |
|  | XM_021460447.1 | P-(S)-hydroxymandelonitrile lyase - *HNL* | 7873.00 | 114.22 |
|  | XM_002447428.2 | bifunctional L-3-cyanoalanine synthase/cysteine synthase 2, mitochondrial - *CAS C1* | 13871.00 | 240.61 |
|  | XM_002452453.2 | bifunctional nitrilase/nitrile hydratase - *NIT4A* | 4077.00 | 71.33 |
|  | XM_021459324.1 | bifunctional nitrilase/nitrile hydratase - *NIT4B2* | 80.00 | 1.24 |
|  | XM_002447182.2 | probable isoaspartyl peptidase/L-asparaginase 2 | 1855.00 | 37.53 |
|  | XM_002464220.2 | isoaspartyl peptidase/L-asparaginase 1 | 125.00 | 2.26 |
|  | XM_021447365.1 | *SbMATE2* | 1323.00 | 18.69 |
|  | XM_002464023.2 | *SbCGTR1* | 1001.00 | 12.67 |
| 05SbC10P5Rep5 | XM_002466054.2 | tyrosine N-monooxygenase - *CYP79A1* | 2748.00 | 30.20 |
|  | XM_002466052.2 | 4-hydroxyphenylacetaldehyde oxime monooxygenase-like - *CYP71E1* | 1915.00 | 21.19 |
|  | XM_002463473.2 | cyanohydrin beta-glucosyltransferase – *UGT85B1* | 783.00 | 10.19 |
|  | XM_002441984.2 | 4-hydroxy-7-methoxy-3-oxo-3,4-dihydro-2H-1,4-benzoxazin-2-yl glucoside beta-D-glucosidase 2, chloroplastic - Dhurrinase 1 | 13.00 | 0.15 |
|  | XM_002443028.2 | 4-hydroxy-7-methoxy-3-oxo-3,4-dihydro-2H-1,4-benzoxazin-2-yl glucoside beta-D-glucosidase 2, chloroplastic - Dhurrinase 2 | 1613.00 | 20.18 |
|  | XM_021446455.1 | Dhurrinase –like 3 | 3.00 | 0.13 |
|  | XM_021460447.1 | P-(S)-hydroxymandelonitrile lyase - *HNL* | 5701.00 | 68.39 |
|  | XM_002447428.2 | bifunctional L-3-cyanoalanine synthase/cysteine synthase 2, mitochondrial - *CAS C1* | 16534.00 | 237.15 |
|  | XM_002452453.2 | bifunctional nitrilase/nitrile hydratase - *NIT4A* | 5380.00 | 77.83 |
|  | XM_021459324.1 | bifunctional nitrilase/nitrile hydratase - *NIT4B2* | 38.00 | 0.49 |
|  | XM_002447182.2 | probable isoaspartyl peptidase/L-asparaginase 2 | 1571.00 | 26.28 |
|  | XM_002464220.2 | isoaspartyl peptidase/L-asparaginase 1 | 170.00 | 2.55 |
|  | XM_021447365.1 | *SbMATE2* | 750.00 | 8.76 |
|  | XM_002464023.2 | *SbCGTR1* | 773.00 | 8.09 |
| 06SbS10P1Rep1 | XM_002466054.2 | tyrosine N-monooxygenase - *CYP79A1* | 7207.00 | 101.54 |
|  | XM_002466052.2 | 4-hydroxyphenylacetaldehyde oxime monooxygenase-like - *CYP71E1* | 2964.00 | 42.06 |
|  | XM_002463473.2 | cyanohydrin beta-glucosyltransferase – *UGT85B1* | 1362.00 | 22.73 |
|  | XM_002441984.2 | 4-hydroxy-7-methoxy-3-oxo-3,4-dihydro-2H-1,4-benzoxazin-2-yl glucoside beta-D-glucosidase 2, chloroplastic - Dhurrinase 1 | 18.00 | 0.27 |
|  | XM_002443028.2 | 4-hydroxy-7-methoxy-3-oxo-3,4-dihydro-2H-1,4-benzoxazin-2-yl glucoside beta-D-glucosidase 2, chloroplastic - Dhurrinase 2 | 14332.00 | 229.95 |
|  | XM_021446455.1 | Dhurrinase –like 3 | 1.00 | 0.06 |
|  | XM_021460447.1 | P-(S)-hydroxymandelonitrile lyase - *HNL* | 14618.00 | 224.85 |
|  | XM_002447428.2 | bifunctional L-3-cyanoalanine synthase/cysteine synthase 2, mitochondrial - *CAS C1* | 14929.00 | 274.56 |
|  | XM_002452453.2 | bifunctional nitrilase/nitrile hydratase - *NIT4A* | 3939.00 | 73.06 |
|  | XM_021459324.1 | bifunctional nitrilase/nitrile hydratase - *NIT4B2* | 22.00 | 0.36 |
|  | XM_002447182.2 | probable isoaspartyl peptidase/L-asparaginase 2 | 1456.00 | 31.23 |
|  | XM_002464220.2 | isoaspartyl peptidase/L-asparaginase 1 | 136.00 | 2.61 |
|  | XM_021447365.1 | *SbMATE2* | 1111.00 | 16.64 |
|  | XM_002464023.2 | *SbCGTR1* | 740.00 | 9.93 |
| 07SbS10P2Rep2 | XM_002466054.2 | tyrosine N-monooxygenase - *CYP79A1* | 4659.00 | 59.92 |
|  | XM_002466052.2 | 4-hydroxyphenylacetaldehyde oxime monooxygenase-like - *CYP71E1* | 2291.00 | 29.67 |
|  | XM_002463473.2 | cyanohydrin beta-glucosyltransferase – *UGT85B1* | 813.00 | 12.39 |
|  | XM_002441984.2 | 4-hydroxy-7-methoxy-3-oxo-3,4-dihydro-2H-1,4-benzoxazin-2-yl glucoside beta-D-glucosidase 2, chloroplastic - Dhurrinase 1 | 26.00 | 0.35 |
|  | XM_002443028.2 | 4-hydroxy-7-methoxy-3-oxo-3,4-dihydro-2H-1,4-benzoxazin-2-yl glucoside beta-D-glucosidase 2, chloroplastic - Dhurrinase 2 | 4272.00 | 62.57 |
|  | XM_021446455.1 | Dhurrinase –like 3 | 4.00 | 0.20 |
|  | XM_021460447.1 | P-(S)-hydroxymandelonitrile lyase - *HNL* | 10933.00 | 153.52 |
|  | XM_002447428.2 | bifunctional L-3-cyanoalanine synthase/cysteine synthase 2, mitochondrial - *CAS C1* | 14830.00 | 248.97 |
|  | XM_002452453.2 | bifunctional nitrilase/nitrile hydratase - *NIT4A* | 3689.00 | 62.46 |
|  | XM_021459324.1 | bifunctional nitrilase/nitrile hydratase - *NIT4B2* | 72.00 | 1.08 |
|  | XM_002447182.2 | probable isoaspartyl peptidase/L-asparaginase 2 | 1535.00 | 30.06 |
|  | XM_002464220.2 | isoaspartyl peptidase/L-asparaginase 1 | 106.00 | 1.86 |
|  | XM_021447365.1 | *SbMATE2* | 1091.00 | 14.92 |
|  | XM_002464023.2 | *SbCGTR1* | 595.00 | 7.29 |
| 08SbS10P3Rep3 | XM_002466054.2 | tyrosine N-monooxygenase - *CYP79A1* | 3166.00 | 40.71 |
|  | XM_002466052.2 | 4-hydroxyphenylacetaldehyde oxime monooxygenase-like - *CYP71E1* | 2429.00 | 31.45 |
|  | XM_002463473.2 | cyanohydrin beta-glucosyltransferase – *UGT85B1* | 1297.00 | 19.76 |
|  | XM_002441984.2 | 4-hydroxy-7-methoxy-3-oxo-3,4-dihydro-2H-1,4-benzoxazin-2-yl glucoside beta-D-glucosidase 2, chloroplastic - Dhurrinase 1 | 18.00 | 0.24 |
|  | XM_002443028.2 | 4-hydroxy-7-methoxy-3-oxo-3,4-dihydro-2H-1,4-benzoxazin-2-yl glucoside beta-D-glucosidase 2, chloroplastic - Dhurrinase 2 | 2053.00 | 30.06 |
|  | XM_021446455.1 | Dhurrinase –like 3 | 4.00 | 0.20 |
|  | XM_021460447.1 | P-(S)-hydroxymandelonitrile lyase - *HNL* | 16261.00 | 228.27 |
|  | XM_002447428.2 | bifunctional L-3-cyanoalanine synthase/cysteine synthase 2, mitochondrial - *CAS C1* | 17381.00 | 291.73 |
|  | XM_002452453.2 | bifunctional nitrilase/nitrile hydratase - *NIT4A* | 14283.00 | 241.79 |
|  | XM_021459324.1 | bifunctional nitrilase/nitrile hydratase - *NIT4B2* | 116.00 | 1.74 |
|  | XM_002447182.2 | probable isoaspartyl peptidase/L-asparaginase 2 | 996.00 | 19.50 |
|  | XM_002464220.2 | isoaspartyl peptidase/L-asparaginase 1 | 126.00 | 2.21 |
|  | XM_021447365.1 | *SbMATE2* | 1024.00 | 14.00 |
|  | XM_002464023.2 | *SbCGTR1* | 823.00 | 10.08 |
| 09SbS10P4Rep4 | XM_002466054.2 | tyrosine N-monooxygenase - *CYP79A1* | 10334.00 | 126.39 |
|  | XM_002466052.2 | 4-hydroxyphenylacetaldehyde oxime monooxygenase-like - *CYP71E1* | 3856.00 | 47.49 |
|  | XM_002463473.2 | cyanohydrin beta-glucosyltransferase – *UGT85B1* | 1211.00 | 17.55 |
|  | XM_002441984.2 | 4-hydroxy-7-methoxy-3-oxo-3,4-dihydro-2H-1,4-benzoxazin-2-yl glucoside beta-D-glucosidase 2, chloroplastic - Dhurrinase 1 | 8.00 | 0.10 |
|  | XM_002443028.2 | 4-hydroxy-7-methoxy-3-oxo-3,4-dihydro-2H-1,4-benzoxazin-2-yl glucoside beta-D-glucosidase 2, chloroplastic - Dhurrinase 2 | 3761.00 | 52.38 |
|  | XM_021446455.1 | Dhurrinase –like 3 | 0.00 | 0.00 |
|  | XM_021460447.1 | P-(S)-hydroxymandelonitrile lyase - *HNL* | 9928.00 | 132.57 |
|  | XM_002447428.2 | bifunctional L-3-cyanoalanine synthase/cysteine synthase 2, mitochondrial - *CAS C1* | 17501.00 | 279.40 |
|  | XM_002452453.2 | bifunctional nitrilase/nitrile hydratase - *NIT4A* | 4850.00 | 78.09 |
|  | XM_021459324.1 | bifunctional nitrilase/nitrile hydratase - *NIT4B2* | 30.00 | 0.43 |
|  | XM_002447182.2 | probable isoaspartyl peptidase/L-asparaginase 2 | 1136.00 | 21.15 |
|  | XM_002464220.2 | isoaspartyl peptidase/L-asparaginase 1 | 188.00 | 3.14 |
|  | XM_021447365.1 | *SbMATE2* | 1434.00 | 18.64 |
|  | XM_002464023.2 | *SbCGTR1* | 492.00 | 5.73 |
| 10SbS10P5Rep5 | XM_002466054.2 | tyrosine N-monooxygenase - *CYP79A1* | 2743.00 | 36.97 |
|  | XM_002466052.2 | 4-hydroxyphenylacetaldehyde oxime monooxygenase-like - *CYP71E1* | 3740.00 | 50.76 |
|  | XM_002463473.2 | cyanohydrin beta-glucosyltransferase – *UGT85B1* | 3413.00 | 54.50 |
|  | XM_002441984.2 | 4-hydroxy-7-methoxy-3-oxo-3,4-dihydro-2H-1,4-benzoxazin-2-yl glucoside beta-D-glucosidase 2, chloroplastic - Dhurrinase 1 | 0.00 | 0.00 |
|  | XM_002443028.2 | 4-hydroxy-7-methoxy-3-oxo-3,4-dihydro-2H-1,4-benzoxazin-2-yl glucoside beta-D-glucosidase 2, chloroplastic - Dhurrinase 2 | 574.00 | 8.81 |
|  | XM_021446455.1 | Dhurrinase –like 3 | 0.00 | 0.00 |
|  | XM_021460447.1 | P-(S)-hydroxymandelonitrile lyase - *HNL* | 24176.00 | 355.75 |
|  | XM_002447428.2 | bifunctional L-3-cyanoalanine synthase/cysteine synthase 2, mitochondrial - *CAS C1* | 11268.00 | 198.24 |
|  | XM_002452453.2 | bifunctional nitrilase/nitrile hydratase - *NIT4A* | 12990.00 | 230.50 |
|  | XM_021459324.1 | bifunctional nitrilase/nitrile hydratase - *NIT4B2* | 50.00 | 0.79 |
|  | XM_002447182.2 | probable isoaspartyl peptidase/L-asparaginase 2 | 926.00 | 19.00 |
|  | XM_002464220.2 | isoaspartyl peptidase/L-asparaginase 1 | 73.00 | 1.34 |
|  | XM_021447365.1 | *SbMATE2* | 1255.00 | 17.98 |
|  | XM_002464023.2 | *SbCGTR1* | 453.00 | 5.82 |
| 11SbC47P6Rep1 | XM_002466054.2 | tyrosine N-monooxygenase - *CYP79A1* | 88.00 | 1.02 |
|  | XM_002466052.2 | 4-hydroxyphenylacetaldehyde oxime monooxygenase-like - *CYP71E1* | 425.00 | 4.98 |
|  | XM_002463473.2 | cyanohydrin beta-glucosyltransferase – *UGT85B1* | 224.00 | 3.09 |
|  | XM_002441984.2 | 4-hydroxy-7-methoxy-3-oxo-3,4-dihydro-2H-1,4-benzoxazin-2-yl glucoside beta-D-glucosidase 2, chloroplastic - Dhurrinase 1 | 43.00 | 0.53 |
|  | XM_002443028.2 | 4-hydroxy-7-methoxy-3-oxo-3,4-dihydro-2H-1,4-benzoxazin-2-yl glucoside beta-D-glucosidase 2, chloroplastic - Dhurrinase 2 | 5569.00 | 73.75 |
|  | XM_021446455.1 | Dhurrinase –like 3 | 3.00 | 0.14 |
|  | XM_021460447.1 | P-(S)-hydroxymandelonitrile lyase - *HNL* | 3224.00 | 40.93 |
|  | XM_002447428.2 | bifunctional L-3-cyanoalanine synthase/cysteine synthase 2, mitochondrial - *CAS C1* | 9755.00 | 148.08 |
|  | XM_002452453.2 | bifunctional nitrilase/nitrile hydratase - *NIT4A* | 7899.00 | 120.94 |
|  | XM_021459324.1 | bifunctional nitrilase/nitrile hydratase - *NIT4B2* | 88.00 | 1.20 |
|  | XM_002447182.2 | probable isoaspartyl peptidase/L-asparaginase 2 | 15629.00 | 276.70 |
|  | XM_002464220.2 | isoaspartyl peptidase/L-asparaginase 1 | 874.00 | 13.86 |
|  | XM_021447365.1 | *SbMATE2* | 284.00 | 3.51 |
|  | XM_002464023.2 | *SbCGTR1* | 1947.00 | 21.56 |
| 12SbC47P7Rep2 | XM_002466054.2 | tyrosine N-monooxygenase - *CYP79A1* | 29.00 | 0.36 |
|  | XM_002466052.2 | 4-hydroxyphenylacetaldehyde oxime monooxygenase-like - *CYP71E1* | 265.00 | 3.28 |
|  | XM_002463473.2 | cyanohydrin beta-glucosyltransferase – *UGT85B1* | 117.00 | 1.70 |
|  | XM_002441984.2 | 4-hydroxy-7-methoxy-3-oxo-3,4-dihydro-2H-1,4-benzoxazin-2-yl glucoside beta-D-glucosidase 2, chloroplastic - Dhurrinase 1 | 5.00 | 0.06 |
|  | XM_002443028.2 | 4-hydroxy-7-methoxy-3-oxo-3,4-dihydro-2H-1,4-benzoxazin-2-yl glucoside beta-D-glucosidase 2, chloroplastic - Dhurrinase 2 | 1915.00 | 26.77 |
|  | XM_021446455.1 | Dhurrinase –like 3 | 3.00 | 0.14 |
|  | XM_021460447.1 | P-(S)-hydroxymandelonitrile lyase - *HNL* | 7463.00 | 100.02 |
|  | XM_002447428.2 | bifunctional L-3-cyanoalanine synthase/cysteine synthase 2, mitochondrial - *CAS C1* | 12018.00 | 192.58 |
|  | XM_002452453.2 | bifunctional nitrilase/nitrile hydratase - *NIT4A* | 9366.00 | 151.37 |
|  | XM_021459324.1 | bifunctional nitrilase/nitrile hydratase - *NIT4B2* | 84.00 | 1.21 |
|  | XM_002447182.2 | probable isoaspartyl peptidase/L-asparaginase 2 | 17963.00 | 335.72 |
|  | XM_002464220.2 | isoaspartyl peptidase/L-asparaginase 1 | 1104.00 | 18.48 |
|  | XM_021447365.1 | *SbMATE2* | 390.00 | 5.09 |
|  | XM_002464023.2 | *SbCGTR1* | 1164.00 | 13.61 |
| 13SbC47P8Rep3 | XM_002466054.2 | tyrosine N-monooxygenase - *CYP79A1* | 749.00 | 9.70 |
|  | XM_002466052.2 | 4-hydroxyphenylacetaldehyde oxime monooxygenase-like - *CYP71E1* | 778.00 | 10.15 |
|  | XM_002463473.2 | cyanohydrin beta-glucosyltransferase – *UGT85B1* | 311.00 | 4.77 |
|  | XM_002441984.2 | 4-hydroxy-7-methoxy-3-oxo-3,4-dihydro-2H-1,4-benzoxazin-2-yl glucoside beta-D-glucosidase 2, chloroplastic - Dhurrinase 1 | 82.00 | 1.12 |
|  | XM_002443028.2 | 4-hydroxy-7-methoxy-3-oxo-3,4-dihydro-2H-1,4-benzoxazin-2-yl glucoside beta-D-glucosidase 2, chloroplastic - Dhurrinase 2 | 16143.00 | 238.09 |
|  | XM_021446455.1 | Dhurrinase –like 3 | 8.00 | 0.41 |
|  | XM_021460447.1 | P-(S)-hydroxymandelonitrile lyase - *HNL* | 8866.00 | 125.36 |
|  | XM_002447428.2 | bifunctional L-3-cyanoalanine synthase/cysteine synthase 2, mitochondrial - *CAS C1* | 10265.00 | 173.54 |
|  | XM_002452453.2 | bifunctional nitrilase/nitrile hydratase - *NIT4A* | 4880.00 | 83.21 |
|  | XM_021459324.1 | bifunctional nitrilase/nitrile hydratase - *NIT4B2* | 62.00 | 0.94 |
|  | XM_002447182.2 | probable isoaspartyl peptidase/L-asparaginase 2 | 21671.00 | 427.30 |
|  | XM_002464220.2 | isoaspartyl peptidase/L-asparaginase 1 | 1286.00 | 22.71 |
|  | XM_021447365.1 | *SbMATE2* | 579.00 | 7.97 |
|  | XM_002464023.2 | *SbCGTR1* | 1825.00 | 22.51 |
| 14SbC47P9Rep4 | XM_002466054.2 | tyrosine N-monooxygenase - *CYP79A1* | 1706.00 | 22.78 |
|  | XM_002466052.2 | 4-hydroxyphenylacetaldehyde oxime monooxygenase-like - *CYP71E1* | 1561.00 | 20.99 |
|  | XM_002463473.2 | cyanohydrin beta-glucosyltransferase – *UGT85B1* | 781.00 | 12.36 |
|  | XM_002441984.2 | 4-hydroxy-7-methoxy-3-oxo-3,4-dihydro-2H-1,4-benzoxazin-2-yl glucoside beta-D-glucosidase 2, chloroplastic - Dhurrinase 1 | 30.00 | 0.42 |
|  | XM_002443028.2 | 4-hydroxy-7-methoxy-3-oxo-3,4-dihydro-2H-1,4-benzoxazin-2-yl glucoside beta-D-glucosidase 2, chloroplastic - Dhurrinase 2 | 9165.00 | 139.36 |
|  | XM_021446455.1 | Dhurrinase –like 3 | 1.00 | 0.05 |
|  | XM_021460447.1 | P-(S)-hydroxymandelonitrile lyase - *HNL* | 5989.00 | 87.31 |
|  | XM_002447428.2 | bifunctional L-3-cyanoalanine synthase/cysteine synthase 2, mitochondrial - *CAS C1* | 7383.00 | 128.68 |
|  | XM_002452453.2 | bifunctional nitrilase/nitrile hydratase - *NIT4A* | 4445.00 | 78.14 |
|  | XM_021459324.1 | bifunctional nitrilase/nitrile hydratase - *NIT4B2* | 54.00 | 0.84 |
|  | XM_002447182.2 | probable isoaspartyl peptidase/L-asparaginase 2 | 12591.00 | 255.95 |
|  | XM_002464220.2 | isoaspartyl peptidase/L-asparaginase 1 | 1030.00 | 18.75 |
|  | XM_021447365.1 | *SbMATE2* | 583.00 | 8.28 |
|  | XM_002464023.2 | *SbCGTR1* | 1531.00 | 19.47 |
| 15SbC47P10Rep5 | XM_002466054.2 | tyrosine N-monooxygenase - *CYP79A1* | 49.00 | 0.73 |
|  | XM_002466052.2 | 4-hydroxyphenylacetaldehyde oxime monooxygenase-like - *CYP71E1* | 407.00 | 6.10 |
|  | XM_002463473.2 | cyanohydrin beta-glucosyltransferase – *UGT85B1* | 197.00 | 3.47 |
|  | XM_002441984.2 | 4-hydroxy-7-methoxy-3-oxo-3,4-dihydro-2H-1,4-benzoxazin-2-yl glucoside beta-D-glucosidase 2, chloroplastic - Dhurrinase 1 | 48.00 | 0.76 |
|  | XM_002443028.2 | 4-hydroxy-7-methoxy-3-oxo-3,4-dihydro-2H-1,4-benzoxazin-2-yl glucoside beta-D-glucosidase 2, chloroplastic - Dhurrinase 2 | 1497.00 | 25.38 |
|  | XM_021446455.1 | Dhurrinase –like 3 | 5.00 | 0.29 |
|  | XM_021460447.1 | P-(S)-hydroxymandelonitrile lyase - *HNL* | 2020.00 | 32.83 |
|  | XM_002447428.2 | bifunctional L-3-cyanoalanine synthase/cysteine synthase 2, mitochondrial - *CAS C1* | 5348.00 | 103.92 |
|  | XM_002452453.2 | bifunctional nitrilase/nitrile hydratase - *NIT4A* | 6235.00 | 122.20 |
|  | XM_021459324.1 | bifunctional nitrilase/nitrile hydratase - *NIT4B2* | 46.00 | 0.80 |
|  | XM_002447182.2 | probable isoaspartyl peptidase/L-asparaginase 2 | 19944.00 | 452.01 |
|  | XM_002464220.2 | isoaspartyl peptidase/L-asparaginase 1 | 1941.00 | 39.40 |
|  | XM_021447365.1 | *SbMATE2* | 679.00 | 10.75 |
|  | XM_002464023.2 | *SbCGTR1* | 1557.00 | 22.08 |
| 16SbS47P6Rep1 | XM_002466054.2 | tyrosine N-monooxygenase - *CYP79A1* | 869.00 | 14.99 |
|  | XM_002466052.2 | 4-hydroxyphenylacetaldehyde oxime monooxygenase-like - *CYP71E1* | 707.00 | 12.28 |
|  | XM_002463473.2 | cyanohydrin beta-glucosyltransferase – *UGT85B1* | 1059.00 | 21.64 |
|  | XM_002441984.2 | 4-hydroxy-7-methoxy-3-oxo-3,4-dihydro-2H-1,4-benzoxazin-2-yl glucoside beta-D-glucosidase 2, chloroplastic - Dhurrinase 1 | 19.00 | 0.35 |
|  | XM_002443028.2 | 4-hydroxy-7-methoxy-3-oxo-3,4-dihydro-2H-1,4-benzoxazin-2-yl glucoside beta-D-glucosidase 2, chloroplastic - Dhurrinase 2 | 1117.00 | 21.94 |
|  | XM_021446455.1 | Dhurrinase –like 3 | 1.00 | 0.07 |
|  | XM_021460447.1 | P-(S)-hydroxymandelonitrile lyase - *HNL* | 108309.00 | 2039.29 |
|  | XM_002447428.2 | bifunctional L-3-cyanoalanine synthase/cysteine synthase 2, mitochondrial - *CAS C1* | 7658.00 | 172.39 |
|  | XM_002452453.2 | bifunctional nitrilase/nitrile hydratase - *NIT4A* | 3724.00 | 84.55 |
|  | XM_021459324.1 | bifunctional nitrilase/nitrile hydratase - *NIT4B2* | 116.00 | 2.34 |
|  | XM_002447182.2 | probable isoaspartyl peptidase/L-asparaginase 2 | 1336.00 | 35.08 |
|  | XM_002464220.2 | isoaspartyl peptidase/L-asparaginase 1 | 46.00 | 1.08 |
|  | XM_021447365.1 | *SbMATE2* | 621.00 | 11.38 |
|  | XM_002464023.2 | *SbCGTR1* | 491.00 | 8.07 |
| 17SbS47P7Rep2 | XM_002466054.2 | tyrosine N-monooxygenase - *CYP79A1* | 488.00 | 6.47 |
|  | XM_002466052.2 | 4-hydroxyphenylacetaldehyde oxime monooxygenase-like - *CYP71E1* | 391.00 | 5.22 |
|  | XM_002463473.2 | cyanohydrin beta-glucosyltransferase – *UGT85B1* | 639.00 | 10.03 |
|  | XM_002441984.2 | 4-hydroxy-7-methoxy-3-oxo-3,4-dihydro-2H-1,4-benzoxazin-2-yl glucoside beta-D-glucosidase 2, chloroplastic - Dhurrinase 1 | 5.00 | 0.07 |
|  | XM_002443028.2 | 4-hydroxy-7-methoxy-3-oxo-3,4-dihydro-2H-1,4-benzoxazin-2-yl glucoside beta-D-glucosidase 2, chloroplastic - Dhurrinase 2 | 1109.00 | 16.74 |
|  | XM_021446455.1 | Dhurrinase –like 3 | 1.00 | 0.05 |
|  | XM_021460447.1 | P-(S)-hydroxymandelonitrile lyase - *HNL* | 113964.00 | 1649.13 |
|  | XM_002447428.2 | bifunctional L-3-cyanoalanine synthase/cysteine synthase 2, mitochondrial - *CAS C1* | 17661.00 | 305.56 |
|  | XM_002452453.2 | bifunctional nitrilase/nitrile hydratase - *NIT4A* | 6512.00 | 113.64 |
|  | XM_021459324.1 | bifunctional nitrilase/nitrile hydratase - *NIT4B2* | 80.00 | 1.24 |
|  | XM_002447182.2 | probable isoaspartyl peptidase/L-asparaginase 2 | 1780.00 | 35.92 |
|  | XM_002464220.2 | isoaspartyl peptidase/L-asparaginase 1 | 68.00 | 1.23 |
|  | XM_021447365.1 | *SbMATE2* | 573.00 | 8.07 |
|  | XM_002464023.2 | *SbCGTR1* | 632.00 | 7.98 |
| 18SbS47P8Rep3 | XM_002466054.2 | tyrosine N-monooxygenase - *CYP79A1* | 564.00 | 7.78 |
|  | XM_002466052.2 | 4-hydroxyphenylacetaldehyde oxime monooxygenase-like - *CYP71E1* | 672.00 | 9.34 |
|  | XM_002463473.2 | cyanohydrin beta-glucosyltransferase – *UGT85B1* | 963.00 | 15.75 |
|  | XM_002441984.2 | 4-hydroxy-7-methoxy-3-oxo-3,4-dihydro-2H-1,4-benzoxazin-2-yl glucoside beta-D-glucosidase 2, chloroplastic - Dhurrinase 1 | 5.00 | 0.07 |
|  | XM_002443028.2 | 4-hydroxy-7-methoxy-3-oxo-3,4-dihydro-2H-1,4-benzoxazin-2-yl glucoside beta-D-glucosidase 2, chloroplastic - Dhurrinase 2 | 2047.00 | 32.17 |
|  | XM_021446455.1 | Dhurrinase –like 3 | 0.00 | 0.00 |
|  | XM_021460447.1 | P-(S)-hydroxymandelonitrile lyase - *HNL* | 64208.00 | 967.51 |
|  | XM_002447428.2 | bifunctional L-3-cyanoalanine synthase/cysteine synthase 2, mitochondrial - *CAS C1* | 12056.00 | 217.20 |
|  | XM_002452453.2 | bifunctional nitrilase/nitrile hydratase - *NIT4A* | 4478.00 | 81.37 |
|  | XM_021459324.1 | bifunctional nitrilase/nitrile hydratase - *NIT4B2* | 43.00 | 0.69 |
|  | XM_002447182.2 | probable isoaspartyl peptidase/L-asparaginase 2 | 3946.00 | 82.91 |
|  | XM_002464220.2 | isoaspartyl peptidase/L-asparaginase 1 | 120.00 | 2.26 |
|  | XM_021447365.1 | *SbMATE2* | 707.00 | 10.37 |
|  | XM_002464023.2 | *SbCGTR1* | 851.00 | 11.19 |
| 19SbS47P9Rep4 | XM_002466054.2 | tyrosine N-monooxygenase - *CYP79A1* | 3017.00 | 35.93 |
|  | XM_002466052.2 | 4-hydroxyphenylacetaldehyde oxime monooxygenase-like - *CYP71E1* | 2434.00 | 29.19 |
|  | XM_002463473.2 | cyanohydrin beta-glucosyltransferase – *UGT85B1* | 1926.00 | 27.17 |
|  | XM_002441984.2 | 4-hydroxy-7-methoxy-3-oxo-3,4-dihydro-2H-1,4-benzoxazin-2-yl glucoside beta-D-glucosidase 2, chloroplastic - Dhurrinase 1 | 1.00 | 0.01 |
|  | XM_002443028.2 | 4-hydroxy-7-methoxy-3-oxo-3,4-dihydro-2H-1,4-benzoxazin-2-yl glucoside beta-D-glucosidase 2, chloroplastic - Dhurrinase 2 | 20.00 | 0.27 |
|  | XM_021446455.1 | Dhurrinase –like 3 | 0.00 | 0.00 |
|  | XM_021460447.1 | P-(S)-hydroxymandelonitrile lyase - *HNL* | 81236.00 | 1056.19 |
|  | XM_002447428.2 | bifunctional L-3-cyanoalanine synthase/cysteine synthase 2, mitochondrial - *CAS C1* | 10390.00 | 161.51 |
|  | XM_002452453.2 | bifunctional nitrilase/nitrile hydratase - *NIT4A* | 14407.00 | 225.88 |
|  | XM_021459324.1 | bifunctional nitrilase/nitrile hydratase - *NIT4B2* | 84.00 | 1.17 |
|  | XM_002447182.2 | probable isoaspartyl peptidase/L-asparaginase 2 | 895.00 | 16.23 |
|  | XM_002464220.2 | isoaspartyl peptidase/L-asparaginase 1 | 67.00 | 1.09 |
|  | XM_021447365.1 | *SbMATE2* | 888.00 | 11.24 |
|  | XM_002464023.2 | *SbCGTR1* | 614.00 | 6.96 |
| 20SbS47P10Rep5 | XM_002466054.2 | tyrosine N-monooxygenase - *CYP79A1* | 2531.00 | 36.27 |
|  | XM_002466052.2 | 4-hydroxyphenylacetaldehyde oxime monooxygenase-like - *CYP71E1* | 2514.00 | 36.28 |
|  | XM_002463473.2 | cyanohydrin beta-glucosyltransferase – *UGT85B1* | 2799.00 | 47.52 |
|  | XM_002441984.2 | 4-hydroxy-7-methoxy-3-oxo-3,4-dihydro-2H-1,4-benzoxazin-2-yl glucoside beta-D-glucosidase 2, chloroplastic - Dhurrinase 1 | 4.00 | 0.06 |
|  | XM_002443028.2 | 4-hydroxy-7-methoxy-3-oxo-3,4-dihydro-2H-1,4-benzoxazin-2-yl glucoside beta-D-glucosidase 2, chloroplastic - Dhurrinase 2 | 541.00 | 8.83 |
|  | XM_021446455.1 | Dhurrinase –like 3 | 0.00 | 0.00 |
|  | XM_021460447.1 | P-(S)-hydroxymandelonitrile lyase - *HNL* | 117857.00 | 1843.98 |
|  | XM_002447428.2 | bifunctional L-3-cyanoalanine synthase/cysteine synthase 2, mitochondrial - *CAS C1* | 7723.00 | 144.47 |
|  | XM_002452453.2 | bifunctional nitrilase/nitrile hydratase - *NIT4A* | 5928.00 | 111.85 |
|  | XM_021459324.1 | bifunctional nitrilase/nitrile hydratase - *NIT4B2* | 51.00 | 0.85 |
|  | XM_002447182.2 | probable isoaspartyl peptidase/L-asparaginase 2 | 1081.00 | 23.58 |
|  | XM_002464220.2 | isoaspartyl peptidase/L-asparaginase 1 | 37.00 | 0.72 |
|  | XM_021447365.1 | *SbMATE2* | 1346.00 | 20.51 |
|  | XM_002464023.2 | *SbCGTR1* | 635 | 8.67 |
| 21SmC10P1Rep1 | XM_002466054.2 | tyrosine N-monooxygenase - *CYP79A1* | 0.0 | 0.0 |
|  | XM_002466052.2 | 4-hydroxyphenylacetaldehyde oxime monooxygenase-like - *CYP71E1* | 41.0 | 0.5 |
|  | XM_002463473.2 | cyanohydrin beta-glucosyltransferase – *UGT85B1* | 3.0 | 0.0 |
|  | XM_002441984.2 | 4-hydroxy-7-methoxy-3-oxo-3,4-dihydro-2H-1,4-benzoxazin-2-yl glucoside beta-D-glucosidase 2, chloroplastic - Dhurrinase 1 | 344.0 | 4.3 |
|  | XM_002443028.2 | 4-hydroxy-7-methoxy-3-oxo-3,4-dihydro-2H-1,4-benzoxazin-2-yl glucoside beta-D-glucosidase 2, chloroplastic - Dhurrinase 2 | 261.0 | 3.5 |
|  | XM_021446455.1 | Dhurrinase –like 3 | 107.0 | 4.9 |
|  | XM_021460447.1 | P-(S)-hydroxymandelonitrile lyase - *HNL* | 1.0 | 0.0 |
|  | XM_002447428.2 | bifunctional L-3-cyanoalanine synthase/cysteine synthase 2, mitochondrial - *CAS C1* | 9356.0 | 142.8 |
|  | XM_002452453.2 | bifunctional nitrilase/nitrile hydratase - *NIT4A* | 2865.0 | 44.1 |
|  | XM_021459324.1 | bifunctional nitrilase/nitrile hydratase - *NIT4B2* | 42.0 | 0.6 |
|  | XM_002447182.2 | probable isoaspartyl peptidase/L-asparaginase 2 | 5507.0 | 98.0 |
|  | XM_002464220.2 | isoaspartyl peptidase/L-asparaginase 1 | 872.0 | 13.9 |
|  | XM_021447365.1 | *SbMATE2* | 85.0 | 1.1 |
|  | XM_002464023.2 | *SbCGTR1* | 6453.0 | 71.9 |
| 22SmC10P2Rep2 | XM_002466054.2 | tyrosine N-monooxygenase - *CYP79A1* | 1.0 | 0.0 |
|  | XM_002466052.2 | 4-hydroxyphenylacetaldehyde oxime monooxygenase-like - *CYP71E1* | 67.0 | 0.8 |
|  | XM_002463473.2 | cyanohydrin beta-glucosyltransferase – *UGT85B1* | 0.0 | 0.0 |
|  | XM_002441984.2 | 4-hydroxy-7-methoxy-3-oxo-3,4-dihydro-2H-1,4-benzoxazin-2-yl glucoside beta-D-glucosidase 2, chloroplastic - Dhurrinase 1 | 527.0 | 6.9 |
|  | XM_002443028.2 | 4-hydroxy-7-methoxy-3-oxo-3,4-dihydro-2H-1,4-benzoxazin-2-yl glucoside beta-D-glucosidase 2, chloroplastic - Dhurrinase 2 | 335.0 | 4.7 |
|  | XM_021446455.1 | Dhurrinase –like 3 | 144.0 | 7.0 |
|  | XM_021460447.1 | P-(S)-hydroxymandelonitrile lyase - *HNL* | 0.0 | 0.0 |
|  | XM_002447428.2 | bifunctional L-3-cyanoalanine synthase/cysteine synthase 2, mitochondrial - *CAS C1* | 12485.0 | 200.9 |
|  | XM_002452453.2 | bifunctional nitrilase/nitrile hydratase - *NIT4A* | 5309.0 | 86.2 |
|  | XM_021459324.1 | bifunctional nitrilase/nitrile hydratase - *NIT4B2* | 68.0 | 1.0 |
|  | XM_002447182.2 | probable isoaspartyl peptidase/L-asparaginase 2 | 11700.0 | 219.6 |
|  | XM_002464220.2 | isoaspartyl peptidase/L-asparaginase 1 | 245.0 | 4.1 |
|  | XM_021447365.1 | *SbMATE2* | 87.0 | 1.1 |
|  | XM_002464023.2 | *SbCGTR1* | 12662.0 | 148.7 |
| 23SmC10P3Rep3 | XM_002466054.2 | tyrosine N-monooxygenase - *CYP79A1* | 4.0 | 0.1 |
|  | XM_002466052.2 | 4-hydroxyphenylacetaldehyde oxime monooxygenase-like - *CYP71E1* | 33.0 | 0.4 |
|  | XM_002463473.2 | cyanohydrin beta-glucosyltransferase – *UGT85B1* | 4.0 | 0.1 |
|  | XM_002441984.2 | 4-hydroxy-7-methoxy-3-oxo-3,4-dihydro-2H-1,4-benzoxazin-2-yl glucoside beta-D-glucosidase 2, chloroplastic - Dhurrinase 1 | 365.0 | 5.0 |
|  | XM_002443028.2 | 4-hydroxy-7-methoxy-3-oxo-3,4-dihydro-2H-1,4-benzoxazin-2-yl glucoside beta-D-glucosidase 2, chloroplastic - Dhurrinase 2 | 204.0 | 3.0 |
|  | XM_021446455.1 | Dhurrinase –like 3 | 85.0 | 4.3 |
|  | XM_021460447.1 | P-(S)-hydroxymandelonitrile lyase - *HNL* | 1.0 | 0.0 |
|  | XM_002447428.2 | bifunctional L-3-cyanoalanine synthase/cysteine synthase 2, mitochondrial - *CAS C1* | 7206.0 | 120.5 |
|  | XM_002452453.2 | bifunctional nitrilase/nitrile hydratase - *NIT4A* | 4195.0 | 70.8 |
|  | XM_021459324.1 | bifunctional nitrilase/nitrile hydratase - *NIT4B2* | 100.0 | 1.5 |
|  | XM_002447182.2 | probable isoaspartyl peptidase/L-asparaginase 2 | 6714.0 | 131.0 |
|  | XM_002464220.2 | isoaspartyl peptidase/L-asparaginase 1 | 411.0 | 7.2 |
|  | XM_021447365.1 | *SbMATE2* | 55.0 | 0.8 |
|  | XM_002464023.2 | *SbCGTR1* | 6899.0 | 84.2 |
| 24SmC10P4Rep4 | XM_002466054.2 | tyrosine N-monooxygenase - *CYP79A1* | 2.0 | 0.0 |
|  | XM_002466052.2 | 4-hydroxyphenylacetaldehyde oxime monooxygenase-like - *CYP71E1* | 30.0 | 0.4 |
|  | XM_002463473.2 | cyanohydrin beta-glucosyltransferase – *UGT85B1* | 2.0 | 0.0 |
|  | XM_002441984.2 | 4-hydroxy-7-methoxy-3-oxo-3,4-dihydro-2H-1,4-benzoxazin-2-yl glucoside beta-D-glucosidase 2, chloroplastic - Dhurrinase 1 | 1131.0 | 14.5 |
|  | XM_002443028.2 | 4-hydroxy-7-methoxy-3-oxo-3,4-dihydro-2H-1,4-benzoxazin-2-yl glucoside beta-D-glucosidase 2, chloroplastic - Dhurrinase 2 | 686.0 | 9.4 |
|  | XM_021446455.1 | Dhurrinase –like 3 | 244.0 | 11.6 |
|  | XM_021460447.1 | P-(S)-hydroxymandelonitrile lyase - *HNL* | 1.0 | 0.0 |
|  | XM_002447428.2 | bifunctional L-3-cyanoalanine synthase/cysteine synthase 2, mitochondrial - *CAS C1* | 8911.0 | 140.6 |
|  | XM_002452453.2 | bifunctional nitrilase/nitrile hydratase - *NIT4A* | 3077.0 | 49.0 |
|  | XM_021459324.1 | bifunctional nitrilase/nitrile hydratase - *NIT4B2* | 30.0 | 0.4 |
|  | XM_002447182.2 | probable isoaspartyl peptidase/L-asparaginase 2 | 7942.0 | 146.1 |
|  | XM_002464220.2 | isoaspartyl peptidase/L-asparaginase 1 | 528.0 | 8.7 |
|  | XM_021447365.1 | *SbMATE2* | 81.0 | 1.0 |
|  | XM_002464023.2 | *SbCGTR1* | 6188.0 | 71.2 |
| 25SmS10P1Rep1 | XM_002466054.2 | tyrosine N-monooxygenase - *CYP79A1* | 0.0 | 0.0 |
|  | XM_002466052.2 | 4-hydroxyphenylacetaldehyde oxime monooxygenase-like - *CYP71E1* | 13.0 | 0.2 |
|  | XM_002463473.2 | cyanohydrin beta-glucosyltransferase – *UGT85B1* | 11.0 | 0.2 |
|  | XM_002441984.2 | 4-hydroxy-7-methoxy-3-oxo-3,4-dihydro-2H-1,4-benzoxazin-2-yl glucoside beta-D-glucosidase 2, chloroplastic - Dhurrinase 1 | 746.0 | 9.9 |
|  | XM_002443028.2 | 4-hydroxy-7-methoxy-3-oxo-3,4-dihydro-2H-1,4-benzoxazin-2-yl glucoside beta-D-glucosidase 2, chloroplastic - Dhurrinase 2 | 503.0 | 7.2 |
|  | XM_021446455.1 | Dhurrinase –like 3 | 206.0 | 10.2 |
|  | XM_021460447.1 | P-(S)-hydroxymandelonitrile lyase - *HNL* | 0.0 | 0.0 |
|  | XM_002447428.2 | bifunctional L-3-cyanoalanine synthase/cysteine synthase 2, mitochondrial - *CAS C1* | 2040.0 | 33.5 |
|  | XM_002452453.2 | bifunctional nitrilase/nitrile hydratase - *NIT4A* | 17257.0 | 285.6 |
|  | XM_021459324.1 | bifunctional nitrilase/nitrile hydratase - *NIT4B2* | 221.0 | 3.3 |
|  | XM_002447182.2 | probable isoaspartyl peptidase/L-asparaginase 2 | 400.0 | 7.7 |
|  | XM_002464220.2 | isoaspartyl peptidase/L-asparaginase 1 | 46.0 | 0.8 |
|  | XM_021447365.1 | *SbMATE2* | 24.0 | 0.3 |
|  | XM_002464023.2 | *SbCGTR1* | 2350.0 | 28.1 |
| 26SmS10P2Rep2 | XM_002466054.2 | tyrosine N-monooxygenase - *CYP79A1* | 0.0 | 0.0 |
|  | XM_002466052.2 | 4-hydroxyphenylacetaldehyde oxime monooxygenase-like - *CYP71E1* | 12.0 | 0.2 |
|  | XM_002463473.2 | cyanohydrin beta-glucosyltransferase – *UGT85B1* | 0.0 | 0.0 |
|  | XM_002441984.2 | 4-hydroxy-7-methoxy-3-oxo-3,4-dihydro-2H-1,4-benzoxazin-2-yl glucoside beta-D-glucosidase 2, chloroplastic - Dhurrinase 1 | 5513.0 | 84.4 |
|  | XM_002443028.2 | 4-hydroxy-7-methoxy-3-oxo-3,4-dihydro-2H-1,4-benzoxazin-2-yl glucoside beta-D-glucosidase 2, chloroplastic - Dhurrinase 2 | 3556.0 | 58.7 |
|  | XM_021446455.1 | Dhurrinase –like 3 | 1370.0 | 78.0 |
|  | XM_021460447.1 | P-(S)-hydroxymandelonitrile lyase - *HNL* | 7.0 | 0.1 |
|  | XM_002447428.2 | bifunctional L-3-cyanoalanine synthase/cysteine synthase 2, mitochondrial - *CAS C1* | 3474.0 | 65.7 |
|  | XM_002452453.2 | bifunctional nitrilase/nitrile hydratase - *NIT4A* | 8901.0 | 169.7 |
|  | XM_021459324.1 | bifunctional nitrilase/nitrile hydratase - *NIT4B2* | 102.0 | 1.7 |
|  | XM_002447182.2 | probable isoaspartyl peptidase/L-asparaginase 2 | 64.0 | 1.4 |
|  | XM_002464220.2 | isoaspartyl peptidase/L-asparaginase 1 | 24.0 | 0.5 |
|  | XM_021447365.1 | *SbMATE2* | 27.0 | 0.4 |
|  | XM_002464023.2 | *SbCGTR1* | 2817.0 | 38.9 |
| 27SmS10P3Rep3 | XM_002466054.2 | tyrosine N-monooxygenase - *CYP79A1* | 0.0 | 0.0 |
|  | XM_002466052.2 | 4-hydroxyphenylacetaldehyde oxime monooxygenase-like - *CYP71E1* | 17.0 | 0.2 |
|  | XM_002463473.2 | cyanohydrin beta-glucosyltransferase – *UGT85B1* | 7.0 | 0.1 |
|  | XM_002441984.2 | 4-hydroxy-7-methoxy-3-oxo-3,4-dihydro-2H-1,4-benzoxazin-2-yl glucoside beta-D-glucosidase 2, chloroplastic - Dhurrinase 1 | 7137.0 | 95.7 |
|  | XM_002443028.2 | 4-hydroxy-7-methoxy-3-oxo-3,4-dihydro-2H-1,4-benzoxazin-2-yl glucoside beta-D-glucosidase 2, chloroplastic - Dhurrinase 2 | 5024.0 | 72.5 |
|  | XM_021446455.1 | Dhurrinase –like 3 | 2083.0 | 103.8 |
|  | XM_021460447.1 | P-(S)-hydroxymandelonitrile lyase - *HNL* | 2.0 | 0.0 |
|  | XM_002447428.2 | bifunctional L-3-cyanoalanine synthase/cysteine synthase 2, mitochondrial - *CAS C1* | 2109.0 | 34.9 |
|  | XM_002452453.2 | bifunctional nitrilase/nitrile hydratase - *NIT4A* | 10234.0 | 170.8 |
|  | XM_021459324.1 | bifunctional nitrilase/nitrile hydratase - *NIT4B2* | 210.0 | 3.1 |
|  | XM_002447182.2 | probable isoaspartyl peptidase/L-asparaginase 2 | 175.0 | 3.4 |
|  | XM_002464220.2 | isoaspartyl peptidase/L-asparaginase 1 | 38.0 | 0.7 |
|  | XM_021447365.1 | *SbMATE2* | 25.0 | 0.3 |
|  | XM_002464023.2 | *SbCGTR1* | 1684.0 | 20.3 |
| 28SmS10P4Rep4 | XM_002466054.2 | tyrosine N-monooxygenase - *CYP79A1* | 0.0 | 0.0 |
|  | XM_002466052.2 | 4-hydroxyphenylacetaldehyde oxime monooxygenase-like - *CYP71E1* | 6.0 | 0.1 |
|  | XM_002463473.2 | cyanohydrin beta-glucosyltransferase – *UGT85B1* | 0.0 | 0.0 |
|  | XM_002441984.2 | 4-hydroxy-7-methoxy-3-oxo-3,4-dihydro-2H-1,4-benzoxazin-2-yl glucoside beta-D-glucosidase 2, chloroplastic - Dhurrinase 1 | 1480.0 | 23.9 |
|  | XM_002443028.2 | 4-hydroxy-7-methoxy-3-oxo-3,4-dihydro-2H-1,4-benzoxazin-2-yl glucoside beta-D-glucosidase 2, chloroplastic - Dhurrinase 2 | 809.0 | 14.0 |
|  | XM_021446455.1 | Dhurrinase –like 3 | 332.0 | 19.9 |
|  | XM_021460447.1 | P-(S)-hydroxymandelonitrile lyase - *HNL* | 0.0 | 0.0 |
|  | XM_002447428.2 | bifunctional L-3-cyanoalanine synthase/cysteine synthase 2, mitochondrial - *CAS C1* | 4113.0 | 81.8 |
|  | XM_002452453.2 | bifunctional nitrilase/nitrile hydratase - *NIT4A* | 28177.0 | 565.4 |
|  | XM_021459324.1 | bifunctional nitrilase/nitrile hydratase - *NIT4B2* | 176.0 | 3.1 |
|  | XM_002447182.2 | probable isoaspartyl peptidase/L-asparaginase 2 | 76.0 | 1.8 |
|  | XM_002464220.2 | isoaspartyl peptidase/L-asparaginase 1 | 16.0 | 0.3 |
|  | XM_021447365.1 | *SbMATE2* | 24.0 | 0.4 |
|  | XM_002464023.2 | *SbCGTR1* | 1980.0 | 28.7 |
| 29SmC18P5Rep1 | XM_002466054.2 | tyrosine N-monooxygenase - *CYP79A1* | 1.0 | 0.0 |
|  | XM_002466052.2 | 4-hydroxyphenylacetaldehyde oxime monooxygenase-like - *CYP71E1* | 61.0 | 0.8 |
|  | XM_002463473.2 | cyanohydrin beta-glucosyltransferase – *UGT85B1* | 1.0 | 0.0 |
|  | XM_002441984.2 | 4-hydroxy-7-methoxy-3-oxo-3,4-dihydro-2H-1,4-benzoxazin-2-yl glucoside beta-D-glucosidase 2, chloroplastic - Dhurrinase 1 | 241.0 | 3.3 |
|  | XM_002443028.2 | 4-hydroxy-7-methoxy-3-oxo-3,4-dihydro-2H-1,4-benzoxazin-2-yl glucoside beta-D-glucosidase 2, chloroplastic - Dhurrinase 2 | 132.0 | 2.0 |
|  | XM_021446455.1 | Dhurrinase –like 3 | 44.0 | 2.2 |
|  | XM_021460447.1 | P-(S)-hydroxymandelonitrile lyase - *HNL* | 1.0 | 0.0 |
|  | XM_002447428.2 | bifunctional L-3-cyanoalanine synthase/cysteine synthase 2, mitochondrial - *CAS C1* | 7131.0 | 120.5 |
|  | XM_002452453.2 | bifunctional nitrilase/nitrile hydratase - *NIT4A* | 1761.0 | 30.0 |
|  | XM_021459324.1 | bifunctional nitrilase/nitrile hydratase - *NIT4B2* | 50.0 | 0.8 |
|  | XM_002447182.2 | probable isoaspartyl peptidase/L-asparaginase 2 | 5883.0 | 116.0 |
|  | XM_002464220.2 | isoaspartyl peptidase/L-asparaginase 1 | 797.0 | 14.1 |
|  | XM_021447365.1 | *SbMATE2* | 81.0 | 1.1 |
|  | XM_002464023.2 | *SbCGTR1* | 5438.0 | 67.1 |
| 30SmC18P6Rep2 | XM_002466054.2 | tyrosine N-monooxygenase - *CYP79A1* | 0.0 | 0.0 |
|  | XM_002466052.2 | 4-hydroxyphenylacetaldehyde oxime monooxygenase-like - *CYP71E1* | 81.0 | 1.2 |
|  | XM_002463473.2 | cyanohydrin beta-glucosyltransferase – *UGT85B1* | 2.0 | 0.0 |
|  | XM_002441984.2 | 4-hydroxy-7-methoxy-3-oxo-3,4-dihydro-2H-1,4-benzoxazin-2-yl glucoside beta-D-glucosidase 2, chloroplastic - Dhurrinase 1 | 231.0 | 3.5 |
|  | XM_002443028.2 | 4-hydroxy-7-methoxy-3-oxo-3,4-dihydro-2H-1,4-benzoxazin-2-yl glucoside beta-D-glucosidase 2, chloroplastic - Dhurrinase 2 | 134.0 | 2.2 |
|  | XM_021446455.1 | Dhurrinase –like 3 | 46.0 | 2.6 |
|  | XM_021460447.1 | P-(S)-hydroxymandelonitrile lyase - *HNL* | 0.0 | 0.0 |
|  | XM_002447428.2 | bifunctional L-3-cyanoalanine synthase/cysteine synthase 2, mitochondrial - *CAS C1* | 7254.0 | 135.0 |
|  | XM_002452453.2 | bifunctional nitrilase/nitrile hydratase - *NIT4A* | 2496.0 | 46.9 |
|  | XM_021459324.1 | bifunctional nitrilase/nitrile hydratase - *NIT4B2* | 61.0 | 1.0 |
|  | XM_002447182.2 | probable isoaspartyl peptidase/L-asparaginase 2 | 9697.0 | 210.5 |
|  | XM_002464220.2 | isoaspartyl peptidase/L-asparaginase 1 | 1017.0 | 19.8 |
|  | XM_021447365.1 | *SbMATE2* | 118.0 | 1.8 |
|  | XM_002464023.2 | *SbCGTR1* | 9589.0 | 130.2 |
| 31SmC18P8Rep3 | XM_002466054.2 | tyrosine N-monooxygenase - *CYP79A1* | 0.0 | 0.0 |
|  | XM_002466052.2 | 4-hydroxyphenylacetaldehyde oxime monooxygenase-like - *CYP71E1* | 125.0 | 1.5 |
|  | XM_002463473.2 | cyanohydrin beta-glucosyltransferase – *UGT85B1* | 0.0 | 0.0 |
|  | XM_002441984.2 | 4-hydroxy-7-methoxy-3-oxo-3,4-dihydro-2H-1,4-benzoxazin-2-yl glucoside beta-D-glucosidase 2, chloroplastic - Dhurrinase 1 | 216.0 | 2.8 |
|  | XM_002443028.2 | 4-hydroxy-7-methoxy-3-oxo-3,4-dihydro-2H-1,4-benzoxazin-2-yl glucoside beta-D-glucosidase 2, chloroplastic - Dhurrinase 2 | 114.0 | 1.6 |
|  | XM_021446455.1 | Dhurrinase –like 3 | 48.0 | 2.3 |
|  | XM_021460447.1 | P-(S)-hydroxymandelonitrile lyase - *HNL* | 0.0 | 0.0 |
|  | XM_002447428.2 | bifunctional L-3-cyanoalanine synthase/cysteine synthase 2, mitochondrial - *CAS C1* | 7636.0 | 121.6 |
|  | XM_002452453.2 | bifunctional nitrilase/nitrile hydratase - *NIT4A* | 3045.0 | 48.9 |
|  | XM_021459324.1 | bifunctional nitrilase/nitrile hydratase - *NIT4B2* | 67.0 | 1.0 |
|  | XM_002447182.2 | probable isoaspartyl peptidase/L-asparaginase 2 | 11380.0 | 211.3 |
|  | XM_002464220.2 | isoaspartyl peptidase/L-asparaginase 1 | 309.0 | 5.1 |
|  | XM_021447365.1 | *SbMATE2* | 111.0 | 1.4 |
|  | XM_002464023.2 | *SbCGTR1* | 4653.0 | 54.0 |
| 32SmC18P9Rep4 | XM_002466054.2 | tyrosine N-monooxygenase - *CYP79A1* | 0.0 | 0.0 |
|  | XM_002466052.2 | 4-hydroxyphenylacetaldehyde oxime monooxygenase-like - *CYP71E1* | 175.0 | 2.4 |
|  | XM_002463473.2 | cyanohydrin beta-glucosyltransferase – *UGT85B1* | 0.0 | 0.0 |
|  | XM_002441984.2 | 4-hydroxy-7-methoxy-3-oxo-3,4-dihydro-2H-1,4-benzoxazin-2-yl glucoside beta-D-glucosidase 2, chloroplastic - Dhurrinase 1 | 198.0 | 2.9 |
|  | XM_002443028.2 | 4-hydroxy-7-methoxy-3-oxo-3,4-dihydro-2H-1,4-benzoxazin-2-yl glucoside beta-D-glucosidase 2, chloroplastic - Dhurrinase 2 | 116.0 | 1.8 |
|  | XM_021446455.1 | Dhurrinase –like 3 | 48.0 | 2.6 |
|  | XM_021460447.1 | P-(S)-hydroxymandelonitrile lyase - *HNL* | 2.0 | 0.0 |
|  | XM_002447428.2 | bifunctional L-3-cyanoalanine synthase/cysteine synthase 2, mitochondrial - *CAS C1* | 2569.0 | 46.4 |
|  | XM_002452453.2 | bifunctional nitrilase/nitrile hydratase - *NIT4A* | 2619.0 | 47.7 |
|  | XM_021459324.1 | bifunctional nitrilase/nitrile hydratase - *NIT4B2* | 19.0 | 0.3 |
|  | XM_002447182.2 | probable isoaspartyl peptidase/L-asparaginase 2 | 5919.0 | 124.7 |
|  | XM_002464220.2 | isoaspartyl peptidase/L-asparaginase 1 | 759.0 | 14.3 |
|  | XM_021447365.1 | *SbMATE2* | 59.0 | 0.9 |
|  | XM_002464023.2 | *SbCGTR1* | 5658.0 | 74.5 |
| 33SmS18P5Rep1 | XM_002466054.2 | tyrosine N-monooxygenase - *CYP79A1* | 0.0 | 0.0 |
|  | XM_002466052.2 | 4-hydroxyphenylacetaldehyde oxime monooxygenase-like - *CYP71E1* | 518.0 | 8.5 |
|  | XM_002463473.2 | cyanohydrin beta-glucosyltransferase – *UGT85B1* | 4.0 | 0.1 |
|  | XM_002441984.2 | 4-hydroxy-7-methoxy-3-oxo-3,4-dihydro-2H-1,4-benzoxazin-2-yl glucoside beta-D-glucosidase 2, chloroplastic - Dhurrinase 1 | 3821.0 | 65.6 |
|  | XM_002443028.2 | 4-hydroxy-7-methoxy-3-oxo-3,4-dihydro-2H-1,4-benzoxazin-2-yl glucoside beta-D-glucosidase 2, chloroplastic - Dhurrinase 2 | 2581.0 | 47.7 |
|  | XM_021446455.1 | Dhurrinase –like 3 | 881.0 | 56.2 |
|  | XM_021460447.1 | P-(S)-hydroxymandelonitrile lyase - *HNL* | 2.0 | 0.0 |
|  | XM_002447428.2 | bifunctional L-3-cyanoalanine synthase/cysteine synthase 2, mitochondrial - *CAS C1* | 6571.0 | 139.3 |
|  | XM_002452453.2 | bifunctional nitrilase/nitrile hydratase - *NIT4A* | 7051.0 | 150.8 |
|  | XM_021459324.1 | bifunctional nitrilase/nitrile hydratase - *NIT4B2* | 97.0 | 1.8 |
|  | XM_002447182.2 | probable isoaspartyl peptidase/L-asparaginase 2 | 301.0 | 7.4 |
|  | XM_002464220.2 | isoaspartyl peptidase/L-asparaginase 1 | 29.0 | 0.6 |
|  | XM_021447365.1 | *SbMATE2* | 61.0 | 1.1 |
|  | XM_002464023.2 | *SbCGTR1* | 2072.0 | 32.1 |
| 34SmS18P6Rep2 | XM_002466054.2 | tyrosine N-monooxygenase - *CYP79A1* | 0.0 | 0.0 |
|  | XM_002466052.2 | 4-hydroxyphenylacetaldehyde oxime monooxygenase-like - *CYP71E1* | 6.0 | 0.1 |
|  | XM_002463473.2 | cyanohydrin beta-glucosyltransferase – *UGT85B1* | 27.0 | 0.6 |
|  | XM_002441984.2 | 4-hydroxy-7-methoxy-3-oxo-3,4-dihydro-2H-1,4-benzoxazin-2-yl glucoside beta-D-glucosidase 2, chloroplastic - Dhurrinase 1 | 4795.0 | 91.7 |
|  | XM_002443028.2 | 4-hydroxy-7-methoxy-3-oxo-3,4-dihydro-2H-1,4-benzoxazin-2-yl glucoside beta-D-glucosidase 2, chloroplastic - Dhurrinase 2 | 2887.0 | 59.5 |
|  | XM_021446455.1 | Dhurrinase –like 3 | 1277.0 | 90.8 |
|  | XM_021460447.1 | P-(S)-hydroxymandelonitrile lyase - *HNL* | 0.0 | 0.0 |
|  | XM_002447428.2 | bifunctional L-3-cyanoalanine synthase/cysteine synthase 2, mitochondrial - *CAS C1* | 2096.0 | 49.5 |
|  | XM_002452453.2 | bifunctional nitrilase/nitrile hydratase - *NIT4A* | 2582.0 | 61.5 |
|  | XM_021459324.1 | bifunctional nitrilase/nitrile hydratase - *NIT4B2* | 17.0 | 0.4 |
|  | XM_002447182.2 | probable isoaspartyl peptidase/L-asparaginase 2 | 721.0 | 19.9 |
|  | XM_002464220.2 | isoaspartyl peptidase/L-asparaginase 1 | 34.0 | 0.8 |
|  | XM_021447365.1 | *SbMATE2* | 32.0 | 0.6 |
|  | XM_002464023.2 | *SbCGTR1* | 2719.0 | 46.8 |
| 35SmS18P7Rep3 | XM_002466054.2 | tyrosine N-monooxygenase - *CYP79A1* | 0.0 | 0.0 |
|  | XM_002466052.2 | 4-hydroxyphenylacetaldehyde oxime monooxygenase-like - *CYP71E1* | 30.0 | 0.4 |
|  | XM_002463473.2 | cyanohydrin beta-glucosyltransferase – *UGT85B1* | 4.0 | 0.1 |
|  | XM_002441984.2 | 4-hydroxy-7-methoxy-3-oxo-3,4-dihydro-2H-1,4-benzoxazin-2-yl glucoside beta-D-glucosidase 2, chloroplastic - Dhurrinase 1 | 6113.0 | 91.0 |
|  | XM_002443028.2 | 4-hydroxy-7-methoxy-3-oxo-3,4-dihydro-2H-1,4-benzoxazin-2-yl glucoside beta-D-glucosidase 2, chloroplastic - Dhurrinase 2 | 4098.0 | 65.7 |
|  | XM_021446455.1 | Dhurrinase –like 3 | 1655.0 | 91.6 |
|  | XM_021460447.1 | P-(S)-hydroxymandelonitrile lyase - *HNL* | 1.0 | 0.0 |
|  | XM_002447428.2 | bifunctional L-3-cyanoalanine synthase/cysteine synthase 2, mitochondrial - *CAS C1* | 4368.0 | 80.3 |
|  | XM_002452453.2 | bifunctional nitrilase/nitrile hydratase - *NIT4A* | 3221.0 | 59.7 |
|  | XM_021459324.1 | bifunctional nitrilase/nitrile hydratase - *NIT4B2* | 32.0 | 0.5 |
|  | XM_002447182.2 | probable isoaspartyl peptidase/L-asparaginase 2 | 252.0 | 5.4 |
|  | XM_002464220.2 | isoaspartyl peptidase/L-asparaginase 1 | 28.0 | 0.5 |
|  | XM_021447365.1 | *SbMATE2* | 59.0 | 0.9 |
|  | XM_002464023.2 | *SbCGTR1* | 3408.0 | 45.7 |
| 36SmS18P6Rep4 | XM_002466054.2 | tyrosine N-monooxygenase - *CYP79A1* | 0.0 | 0.0 |
|  | XM_002466052.2 | 4-hydroxyphenylacetaldehyde oxime monooxygenase-like - *CYP71E1* | 166.0 | 2.8 |
|  | XM_002463473.2 | cyanohydrin beta-glucosyltransferase – *UGT85B1* | 9.0 | 0.2 |
|  | XM_002441984.2 | 4-hydroxy-7-methoxy-3-oxo-3,4-dihydro-2H-1,4-benzoxazin-2-yl glucoside beta-D-glucosidase 2, chloroplastic - Dhurrinase 1 | 5549.0 | 96.5 |
|  | XM_002443028.2 | 4-hydroxy-7-methoxy-3-oxo-3,4-dihydro-2H-1,4-benzoxazin-2-yl glucoside beta-D-glucosidase 2, chloroplastic - Dhurrinase 2 | 3630.0 | 68.0 |
|  | XM_021446455.1 | Dhurrinase –like 3 | 1413.0 | 91.3 |
|  | XM_021460447.1 | P-(S)-hydroxymandelonitrile lyase - *HNL* | 2.0 | 0.0 |
|  | XM_002447428.2 | bifunctional L-3-cyanoalanine synthase/cysteine synthase 2, mitochondrial - *CAS C1* | 4373.0 | 93.9 |
|  | XM_002452453.2 | bifunctional nitrilase/nitrile hydratase - *NIT4A* | 3404.0 | 73.7 |
|  | XM_021459324.1 | bifunctional nitrilase/nitrile hydratase - *NIT4B2* | 23.0 | 0.4 |
|  | XM_002447182.2 | probable isoaspartyl peptidase/L-asparaginase 2 | 1011.0 | 25.3 |
|  | XM_002464220.2 | isoaspartyl peptidase/L-asparaginase 1 | 50.0 | 1.1 |
|  | XM_021447365.1 | *SbMATE2* | 24.0 | 0.4 |
|  | XM_002464023.2 | *SbCGTR1* | 3810.0 | 59.7 |

**Note:** Sb: *S. bicolor*, Sm: *S. macrospermum*, C: Control, S: Water-stressed, P: Plant, Rep: replicate

**Table S9:** Differentially expressed genes of in dhurrin biosynthesis, recycling, bio-activation and HCN detoxification in dependence of the age of the plant issue and imposed water-stressed growth of *S. bicolor* and *S. macrospermum*

| ***Comparison group*** | **Enzyme Code** | **Gene ID** | **Description** | **Log2 Fold Change** | **Up-/down-regulated** |
| --- | --- | --- | --- | --- | --- |
| *S. bicolor 10 d Control to 47 d Control* | 1.14.14.36 | **XM_002466054.2** | tyrosine N-monooxygenase - *CYP79A1* | -4.28 | Down |
|  |  | Sobic_001G012300 |  |  |  |
|  | 1.14.14.37 | **XM_002466052.2** | 4-hydroxyphenylacetaldehyde oxime monooxygenase-like - *CYP71E1* | -3.01 | Down |
|  |  | Sobic.001G012200 |  |  |  |
|  | 2.4.1.85 | **XM_002463473.2** | cyanohydrin beta-glucosyltransferase – *UGT85B1* | -2.81 | Down |
|  |  | Sobic.001G012400 |  |  |  |
|  | 3.2.1.21 | **XM_002441984.2** | 4-hydroxy-7-methoxy-3-oxo-3,4-dihydro-2H-1,4-benzoxazin-2-yl glucoside beta-D-glucosidase 2, chloroplastic - Dhurrinase 1 | - | - |
|  |  | Sobic.008G079800 |  |  |  |
|  |  | **XM_002443028.2** | 4-hydroxy-7-methoxy-3-oxo-3,4-dihydro-2H-1,4-benzoxazin-2-yl glucoside beta-D-glucosidase 2, chloroplastic - Dhurrinase 2 | - | - |
|  |  | Sobic.008G080400 |  |  |  |
|  |  | **XM_021446455.1** | Dhurrinase –like 3 | - | - |
|  |  | Sobic.008G080100 |  |  |  |
|  | 4.1.2.11 | **XM_021460447.1** | P-(S)-hydroxymandelonitrile lyase - *HNL* | -1.36 | Down |
|  |  | Sobic.004G335500 |  |  |  |
|  | 4.4.1.9 | **XM_002447428.2** | bifunctional L-3-cyanoalanine synthase/cysteine synthase 2, mitochondrial - *CAS C1* | -1.18 | Down |
|  |  | Sobic.006G016900 |  |  |  |
|  | 3.5.5.1 | **XM_002452453.2** | bifunctional nitrilase/nitrile hydratase - *NIT4A* | - | - |
|  |  | Sobic.004G225200 |  |  |  |
|  |  | **XM_021459324.1** | bifunctional nitrilase/nitrile hydratase - *NIT4B2* | - | - |
|  |  | Sobic.004G225100 |  |  |  |
|  | 3.5.1.1 | **XM_002447182.2** | probable isoaspartyl peptidase/L-asparaginase 2 | - | - |
|  |  | Sobic.006G243200 |  |  |  |
|  |  | **XM_002464220.2** | isoaspartyl peptidase/L-asparaginase 1 | - | - |
|  |  | Sobic.001G174700 |  |  |  |
|  |  | **XM_021447365.1** | *SbMATE2* | -1.63 | Down |
|  |  | Sobic.001G012600 |  |  |  |
|  |  | **XM_002464023.2** | *SbCGTR1* | - | - |
|  |  | Sobic.001G133900 |  |  |  |
| *S. bicolor 10 d water-stressed to 47 d water-stressed growth* | 1.14.14.36 | **XM_002466054.2** | tyrosine N-monooxygenase - *CYP79A1* | -2.19 | Down |
|  |  | Sobic_001G012300 |  |  |  |
|  | 1.14.14.37 | **XM_002466052.2** | 4-hydroxyphenylacetaldehyde oxime monooxygenase-like - *CYP71E1* | -1.14 | Down |
|  |  | Sobic.001G012200 |  |  |  |
|  | 2.4.1.85 | **XM_002463473.2** | cyanohydrin beta-glucosyltransferase – *UGT85B1* | - | - |
|  |  | Sobic.001G012400 |  |  |  |
|  | 3.2.1.21 | **XM_002441984.2** | 4-hydroxy-7-methoxy-3-oxo-3,4-dihydro-2H-1,4-benzoxazin-2-yl glucoside beta-D-glucosidase 2, chloroplastic - Dhurrinase 1 | - | - |
|  |  | Sobic.008G079800 |  |  |  |
|  |  | **XM_002443028.2** | 4-hydroxy-7-methoxy-3-oxo-3,4-dihydro-2H-1,4-benzoxazin-2-yl glucoside beta-D-glucosidase 2, chloroplastic - Dhurrinase 2 | -2.58 | Down |
|  |  | Sobic.008G080400 |  |  |  |
|  |  | **XM_021446455.1** | Dhurrinase –like 3 | - | - |
|  |  | Sobic.008G080100 |  |  |  |
|  | 4.1.2.11 | **XM_021460447.1** | P-(S)-hydroxymandelonitrile lyase - *HNL* | 2.59 | Up |
|  |  | Sobic.004G335500 |  |  |  |
|  | 4.4.1.9 | **XM_002447428.2** | bifunctional L-3-cyanoalanine synthase/cysteine synthase 2, mitochondrial - *CAS C1* | - | - |
|  |  | Sobic.006G016900 |  |  |  |
|  | 3.5.5.1 | **XM_002452453.2** | bifunctional nitrilase/nitrile hydratase - *NIT4A* | - | - |
|  |  | Sobic.004G225200 |  |  |  |
|  |  | **XM_021459324.1** | bifunctional nitrilase/nitrile hydratase - *NIT4B2* | - | - |
|  |  | Sobic.004G225100 |  |  |  |
|  | 3.5.1.1 | **XM_002447182.2** | probable isoaspartyl peptidase/L-asparaginase 2 | - | - |
|  |  | Sobic.006G243200 |  |  |  |
|  |  | **XM_002464220.2** | isoaspartyl peptidase/L-asparaginase 1 | - | - |
|  |  | Sobic.001G174700 |  |  |  |
|  |  | **XM_021447365.1** | *SbMATE2* | - | - |
|  |  | Sobic.001G012600 |  |  |  |
|  |  | **XM_002464023.2** | *SbCGTR1* | - | - |
|  |  | Sobic.001G133900 |  |  |  |
| *S. bicolor 10 d Control to 10 d water-stressed growth* | 1.14.14.36 | **XM_002466054.2** | tyrosine N-monooxygenase - *CYP79A1* | - | - |
|  |  | Sobic_001G012300 |  |  |  |
|  | 1.14.14.37 | **XM_002466052.2** | 4-hydroxyphenylacetaldehyde oxime monooxygenase-like - *CYP71E1* | - | - |
|  |  | Sobic.001G012200 |  |  |  |
|  | 2.4.1.85 | **XM_002463473.2** | cyanohydrin beta-glucosyltransferase – *UGT85B1* | - | - |
|  |  | Sobic.001G012400 |  |  |  |
|  | 3.2.1.21 | **XM_002441984.2** | 4-hydroxy-7-methoxy-3-oxo-3,4-dihydro-2H-1,4-benzoxazin-2-yl glucoside beta-D-glucosidase 2, chloroplastic - Dhurrinase 1 | - | - |
|  |  | Sobic.008G079800 |  |  |  |
|  |  | **XM_002443028.2** | 4-hydroxy-7-methoxy-3-oxo-3,4-dihydro-2H-1,4-benzoxazin-2-yl glucoside beta-D-glucosidase 2, chloroplastic - Dhurrinase 2 | - | - |
|  |  | Sobic.008G080400 |  |  |  |
|  |  | **XM_021446455.1** | Dhurrinase –like 3 | - | - |
|  |  | Sobic.008G080100 |  |  |  |
|  | 4.1.2.11 | **XM_021460447.1** | P-(S)-hydroxymandelonitrile lyase - *HNL* | - | - |
|  |  | Sobic.004G335500 |  |  |  |
|  | 4.4.1.9 | **XM_002447428.2** | bifunctional L-3-cyanoalanine synthase/cysteine synthase 2, mitochondrial - *CAS C1* | - | - |
|  |  | Sobic.006G016900 |  |  |  |
|  | 3.5.5.1 | **XM_002452453.2** | bifunctional nitrilase/nitrile hydratase - *NIT4A* | - | - |
|  |  | Sobic.004G225200 |  |  |  |
|  |  | **XM_021459324.1** | bifunctional nitrilase/nitrile hydratase - *NIT4B2* | - | - |
|  |  | Sobic.004G225100 |  |  |  |
|  | 3.5.1.1 | **XM_002447182.2** | probable isoaspartyl peptidase/L-asparaginase 2 | - | - |
|  |  | Sobic.006G243200 |  |  |  |
|  |  | **XM_002464220.2** | isoaspartyl peptidase/L-asparaginase 1 | - | - |
|  |  | Sobic.001G174700 |  |  |  |
|  |  | **XM_021447365.1** | *SbMATE2* | - | - |
|  |  | Sobic.001G012600 |  |  |  |
|  |  | **XM_002464023.2** | *SbCGTR1* | - | - |
|  |  | Sobic.001G133900 |  |  |  |
| *S. bicolor 47 d Control to 47 d water-stressed growth* | 1.14.14.36 | **XM_002466054.2** | tyrosine N-monooxygenase - *CYP79A1* | - | - |
|  |  | Sobic_001G012300 |  |  |  |
|  | 1.14.14.37 | **XM_002466052.2** | 4-hydroxyphenylacetaldehyde oxime monooxygenase-like - *CYP71E1* | - | - |
|  |  | Sobic.001G012200 |  |  |  |
|  | 2.4.1.85 | **XM_002463473.2** | cyanohydrin beta-glucosyltransferase – *UGT85B1* | 2.19 | Up |
|  |  | Sobic.001G012400 |  |  |  |
|  | 3.2.1.21 | **XM_002441984.2** | 4-hydroxy-7-methoxy-3-oxo-3,4-dihydro-2H-1,4-benzoxazin-2-yl glucoside beta-D-glucosidase 2, chloroplastic - Dhurrinase 1 | - | - |
|  |  | Sobic.008G079800 |  |  |  |
|  |  | **XM_002443028.2** | 4-hydroxy-7-methoxy-3-oxo-3,4-dihydro-2H-1,4-benzoxazin-2-yl glucoside beta-D-glucosidase 2, chloroplastic - Dhurrinase 2 | - | - |
|  |  | Sobic.008G080400 |  |  |  |
|  |  | **XM_021446455.1** | Dhurrinase –like 3 | - | - |
|  |  | Sobic.008G080100 |  |  |  |
|  | 4.1.2.11 | **XM_021460447.1** | P-(S)-hydroxymandelonitrile lyase - *HNL* | 4.26 | Up |
|  |  | Sobic.004G335500 |  |  |  |
|  | 4.4.1.9 | **XM_002447428.2** | bifunctional L-3-cyanoalanine synthase/cysteine synthase 2, mitochondrial - *CAS C1* | - | - |
|  |  | Sobic.006G016900 |  |  |  |
|  | 3.5.5.1 | **XM_002452453.2** | bifunctional nitrilase/nitrile hydratase - *NIT4A* | - | - |
|  |  | Sobic.004G225200 |  |  |  |
|  |  | **XM_021459324.1** | bifunctional nitrilase/nitrile hydratase - *NIT4B2* | - | - |
|  |  | Sobic.004G225100 |  |  |  |
|  | 3.5.1.1 | **XM_002447182.2** | probable isoaspartyl peptidase/L-asparaginase 2 | -3.21 | Down |
|  |  | Sobic.006G243200 |  |  |  |
|  |  | **XM_002464220.2** | isoaspartyl peptidase/L-asparaginase 1 | -4.19 | Down |
|  |  | Sobic.001G174700 |  |  |  |
|  |  | **XM_021447365.1** | *SbMATE2* | - | - |
|  |  | Sobic.001G012600 |  |  |  |
|  |  | **XM_002464023.2** | *SbCGTR1* | - | - |
|  |  | Sobic.001G133900 |  |  |  |
| *S. macrospermum 10 d Control to 18 d Control* | 1.14.14.36 | **XM_002466054.2** | tyrosine N-monooxygenase - *CYP79A1* | - | - |
|  |  | Sobic_001G012300 |  |  |  |
|  | 1.14.14.37 | **XM_002466052.2** | 4-hydroxyphenylacetaldehyde oxime monooxygenase-like - *CYP71E1* | - | - |
|  |  | Sobic.001G012200 |  |  |  |
|  | 2.4.1.85 | **XM_002463473.2** | cyanohydrin beta-glucosyltransferase – *UGT85B1* | - | - |
|  |  | Sobic.001G012400 |  |  |  |
|  | 3.2.1.21 | **XM_002441984.2** | 4-hydroxy-7-methoxy-3-oxo-3,4-dihydro-2H-1,4-benzoxazin-2-yl glucoside beta-D-glucosidase 2, chloroplastic - Dhurrinase 1 | - | - |
|  |  | Sobic.008G079800 |  |  |  |
|  |  | **XM_002443028.2** | 4-hydroxy-7-methoxy-3-oxo-3,4-dihydro-2H-1,4-benzoxazin-2-yl glucoside beta-D-glucosidase 2, chloroplastic - Dhurrinase 2 | - | - |
|  |  | Sobic.008G080400 |  |  |  |
|  |  | **XM_021446455.1** | Dhurrinase –like 3 | - | - |
|  |  | Sobic.008G080100 |  |  |  |
|  | 4.1.2.11 | **XM_021460447.1** | P-(S)-hydroxymandelonitrile lyase - *HNL* | - | - |
|  |  | Sobic.004G335500 |  |  |  |
|  | 4.4.1.9 | **XM_002447428.2** | bifunctional L-3-cyanoalanine synthase/cysteine synthase 2, mitochondrial - *CAS C1* | - | - |
|  |  | Sobic.006G016900 |  |  |  |
|  | 3.5.5.1 | **XM_002452453.2** | bifunctional nitrilase/nitrile hydratase - *NIT4A* | - | - |
|  |  | Sobic.004G225200 |  |  |  |
|  |  | **XM_021459324.1** | bifunctional nitrilase/nitrile hydratase - *NIT4B2* | - | - |
|  |  | Sobic.004G225100 |  |  |  |
|  | 3.5.1.1 | **XM_002447182.2** | probable isoaspartyl peptidase/L-asparaginase 2 | - | - |
|  |  | Sobic.006G243200 |  |  |  |
|  |  | **XM_002464220.2** | isoaspartyl peptidase/L-asparaginase 1 | - | - |
|  |  | Sobic.001G174700 |  |  |  |
|  |  | **XM_021447365.1** | *SbMATE2* | - | - |
|  |  | Sobic.001G012600 |  |  |  |
|  |  | **XM_002464023.2** | *SbCGTR1* | - | - |
|  |  | Sobic.001G133900 |  |  |  |
| *S. macrospermum 10 d water- stressed to 18 d water-stressed growth* | 1.14.14.36 | **XM_002466054.2** | tyrosine N-monooxygenase - *CYP79A1* | - | - |
|  |  | Sobic_001G012300 |  |  |  |
|  | 1.14.14.37 | **XM_002466052.2** | 4-hydroxyphenylacetaldehyde oxime monooxygenase-like - *CYP71E1* | 3.79 | Up |
|  |  | Sobic.001G012200 |  |  |  |
|  | 2.4.1.85 | **XM_002463473.2** | cyanohydrin beta-glucosyltransferase – *UGT85B1* | - | - |
|  |  | Sobic.001G012400 |  |  |  |
|  | 3.2.1.21 | **XM_002441984.2** | 4-hydroxy-7-methoxy-3-oxo-3,4-dihydro-2H-1,4-benzoxazin-2-yl glucoside beta-D-glucosidase 2, chloroplastic - Dhurrinase 1 | - | - |
|  |  | Sobic.008G079800 |  |  |  |
|  |  | **XM_002443028.2** | 4-hydroxy-7-methoxy-3-oxo-3,4-dihydro-2H-1,4-benzoxazin-2-yl glucoside beta-D-glucosidase 2, chloroplastic - Dhurrinase 2 | - | - |
|  |  | Sobic.008G080400 |  |  |  |
|  |  | **XM_021446455.1** | Dhurrinase –like 3 | - | - |
|  |  | Sobic.008G080100 |  |  |  |
|  | 4.1.2.11 | **XM_021460447.1** | P-(S)-hydroxymandelonitrile lyase - *HNL* | - | - |
|  |  | Sobic.004G335500 |  |  |  |
|  | 4.4.1.9 | **XM_002447428.2** | bifunctional L-3-cyanoalanine synthase/cysteine synthase 2, mitochondrial - *CAS C1* | - | - |
|  |  | Sobic.006G016900 |  |  |  |
|  | 3.5.5.1 | **XM_002452453.2** | bifunctional nitrilase/nitrile hydratase - *NIT4A* | -1.97 | Down |
|  |  | Sobic.004G225200 |  |  |  |
|  |  | **XM_021459324.1** | bifunctional nitrilase/nitrile hydratase - *NIT4B2* |  |  |
|  |  | Sobic.004G225100 |  |  |  |
|  | 3.5.1.1 | **XM_002447182.2** | probable isoaspartyl peptidase/L-asparaginase 2 | - | - |
|  |  | Sobic.006G243200 |  |  |  |
|  |  | **XM_002464220.2** | isoaspartyl peptidase/L-asparaginase 1 | - | - |
|  |  | Sobic.001G174700 |  |  |  |
|  |  | **XM_021447365.1** | *SbMATE2* | - | - |
|  |  | Sobic.001G012600 |  |  |  |
|  |  | **XM_002464023.2** | *SbCGTR1* | - | - |
|  |  | Sobic.001G133900 |  |  |  |
| *S. macrospermum 10 d Control to 10 d water-stressed growth* | 1.14.14.36 | **XM_002466054.2** | tyrosine N-monooxygenase - *CYP79A1* | - | - |
|  |  | Sobic_001G012300 |  |  |  |
|  | 1.14.14.37 | **XM_002466052.2** | 4-hydroxyphenylacetaldehyde oxime monooxygenase-like - *CYP71E1* | - | - |
|  |  | Sobic.001G012200 |  |  |  |
|  | 2.4.1.85 | **XM_002463473.2** | cyanohydrin beta-glucosyltransferase – *UGT85B1* | - | - |
|  |  | Sobic.001G012400 |  |  |  |
|  | 3.2.1.21 | **XM_002441984.2** | 4-hydroxy-7-methoxy-3-oxo-3,4-dihydro-2H-1,4-benzoxazin-2-yl glucoside beta-D-glucosidase 2, chloroplastic - Dhurrinase 1 | 2.48 | Up |
|  |  | Sobic.008G079800 |  |  |  |
|  |  | **XM_002443028.2** | 4-hydroxy-7-methoxy-3-oxo-3,4-dihydro-2H-1,4-benzoxazin-2-yl glucoside beta-D-glucosidase 2, chloroplastic - Dhurrinase 2 | 2.57 | Up |
|  |  | Sobic.008G080400 |  |  |  |
|  |  | **XM_021446455.1** | Dhurrinase –like 3 | 2.62 | Up |
|  |  | Sobic.008G080100 |  |  |  |
|  | 4.1.2.11 | **XM_021460447.1** | P-(S)-hydroxymandelonitrile lyase - *HNL* | - | - |
|  |  | Sobic.004G335500 |  |  |  |
|  | 4.4.1.9 | **XM_002447428.2** | bifunctional L-3-cyanoalanine synthase/cysteine synthase 2, mitochondrial - *CAS C1* | -1.97 | Down |
|  |  | Sobic.006G016900 |  |  |  |
|  | 3.5.5.1 | **XM_002452453.2** | bifunctional nitrilase/nitrile hydratase - *NIT4A* | 1.82 | Up |
|  |  | Sobic.004G225200 |  |  |  |
|  |  | **XM_021459324.1** | bifunctional nitrilase/nitrile hydratase - *NIT4B2* | - | - |
|  |  | Sobic.004G225100 |  |  |  |
|  | 3.5.1.1 | **XM_002447182.2** | probable isoaspartyl peptidase/L-asparaginase 2 | -5.65 | Down |
|  |  | Sobic.006G243200 |  |  |  |
|  |  | **XM_002464220.2** | isoaspartyl peptidase/L-asparaginase 1 | -4.32 | Down |
|  |  | Sobic.001G174700 |  |  |  |
|  |  | **XM_021447365.1** | *SbMATE2* | - | - |
|  |  | Sobic.001G012600 |  |  |  |
|  |  | **XM_002464023.2** | *SbCGTR1* | -2.13 | Down |
|  |  | Sobic.001G133900 |  |  |  |
| *S. macrospermum 18 d Control to 18 d water-stressed growth* | 1.14.14.36 | **XM_002466054.2** | tyrosine N-monooxygenase - *CYP79A1* | - | - |
|  |  | Sobic_001G012300 |  |  |  |
|  | 1.14.14.37 | **XM_002466052.2** | 4-hydroxyphenylacetaldehyde oxime monooxygenase-like - *CYP71E1* | - | - |
|  |  | Sobic.001G012200 |  |  |  |
|  | 2.4.1.85 | **XM_002463473.2** | cyanohydrin beta-glucosyltransferase – *UGT85B1* | - | - |
|  |  | Sobic.001G012400 |  |  |  |
|  | 3.2.1.21 | **XM_002441984.2** | 4-hydroxy-7-methoxy-3-oxo-3,4-dihydro-2H-1,4-benzoxazin-2-yl glucoside beta-D-glucosidase 2, chloroplastic - Dhurrinase 1 | 4.27 | Up |
|  |  | Sobic.008G079800 |  |  |  |
|  |  | **XM_002443028.2** | 4-hydroxy-7-methoxy-3-oxo-3,4-dihydro-2H-1,4-benzoxazin-2-yl glucoside beta-D-glucosidase 2, chloroplastic - Dhurrinase 2 | 4.49 | Up |
|  |  | Sobic.008G080400 |  |  |  |
|  |  | **XM_021446455.1** | Dhurrinase –like 3 | 4.56 | Up |
|  |  | Sobic.008G080100 |  |  |  |
|  | 4.1.2.11 | **XM_021460447.1** | P-(S)-hydroxymandelonitrile lyase - *HNL* | - | - |
|  |  | Sobic.004G335500 |  |  |  |
|  | 4.4.1.9 | **XM_002447428.2** | bifunctional L-3-cyanoalanine synthase/cysteine synthase 2, mitochondrial - *CAS C1* | - | - |
|  |  | Sobic.006G016900 |  |  |  |
|  | 3.5.5.1 | **XM_002452453.2** | bifunctional nitrilase/nitrile hydratase - *NIT4A* | - | - |
|  |  | Sobic.004G225200 |  |  |  |
|  |  | **XM_021459324.1** | bifunctional nitrilase/nitrile hydratase - *NIT4B2* | - | - |
|  |  | Sobic.004G225100 |  |  |  |
|  | 3.5.1.1 | **XM_002447182.2** | probable isoaspartyl peptidase/L-asparaginase 2 | -4.21 | Down |
|  |  | Sobic.006G243200 |  |  |  |
|  |  | **XM_002464220.2** | isoaspartyl peptidase/L-asparaginase 1 | -4.63 | Down |
|  |  | Sobic.001G174700 |  |  |  |
|  |  | **XM_021447365.1** | *SbMATE2* | - | - |
|  |  | Sobic.001G012600 |  |  |  |
|  |  | **XM_002464023.2** | *SbCGTR1* | - | - |
|  |  | Sobic.001G133900 |  |  |  |

**
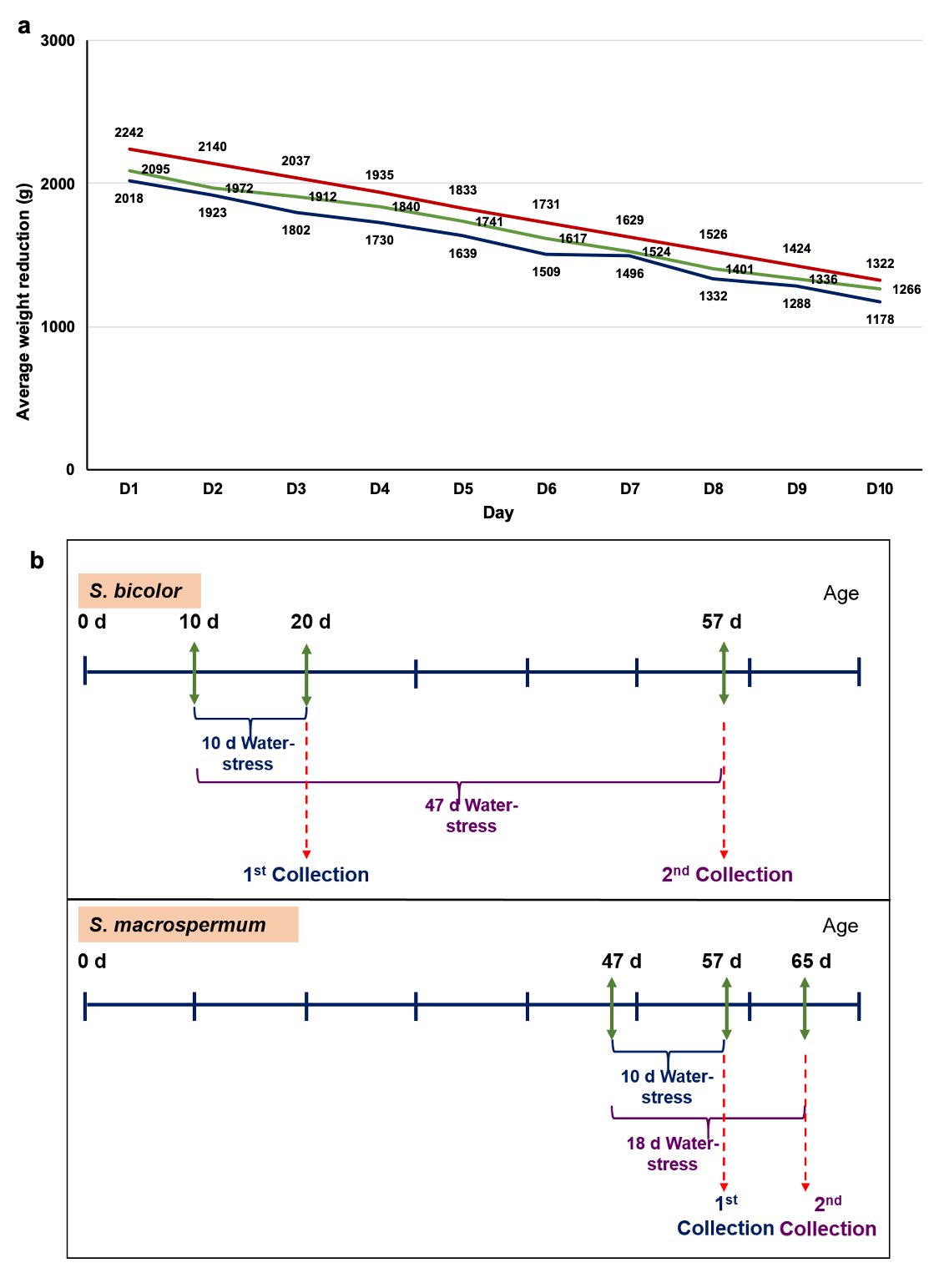
Fig. S1** The effect of water stress and timeline of the sorghum plants. a: Average weight reduction of the plants over 10 days. b: Timelines of the imposed water-stress for *S. bicolor* and *S. macrospermum*


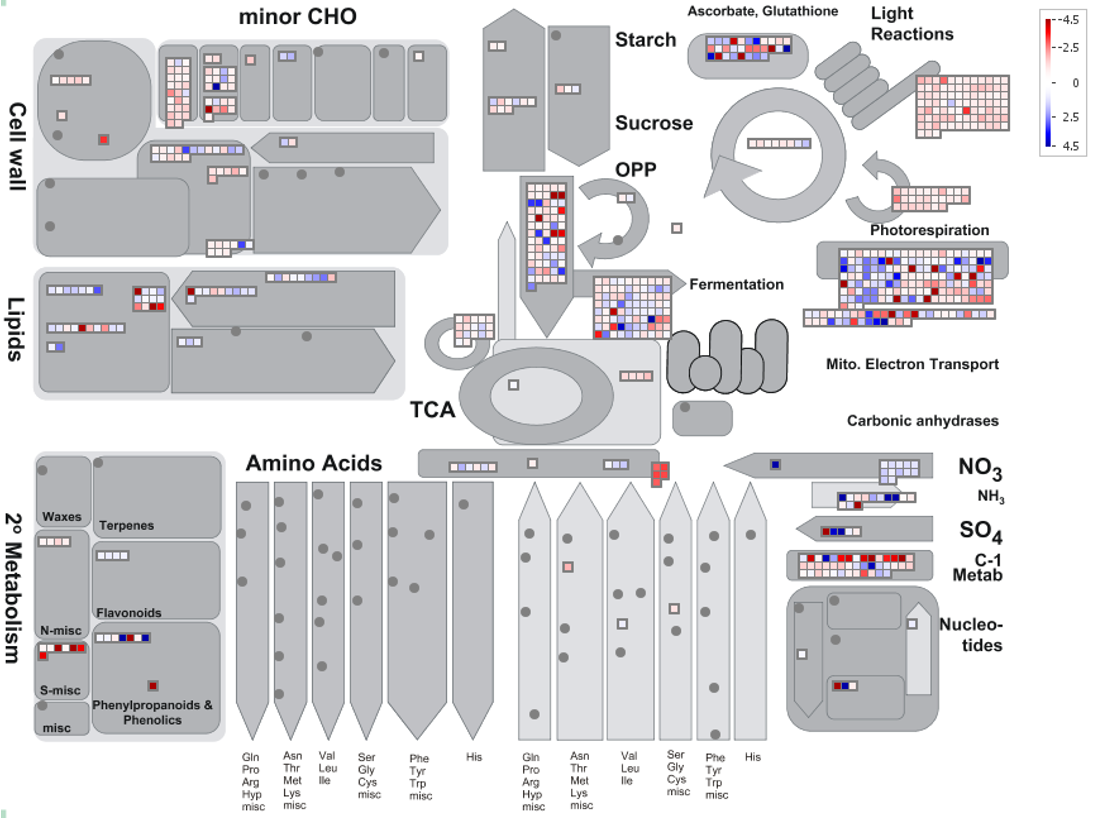


**Fig. S2** Metabolism based overview of differentially expressed genes in S. bicolor Control 10 d to 47 d depending on plant tissue age and water-stressed growth. Red: Down-regulated gene transcript. Blue: Up-regulated gene transcripts

**
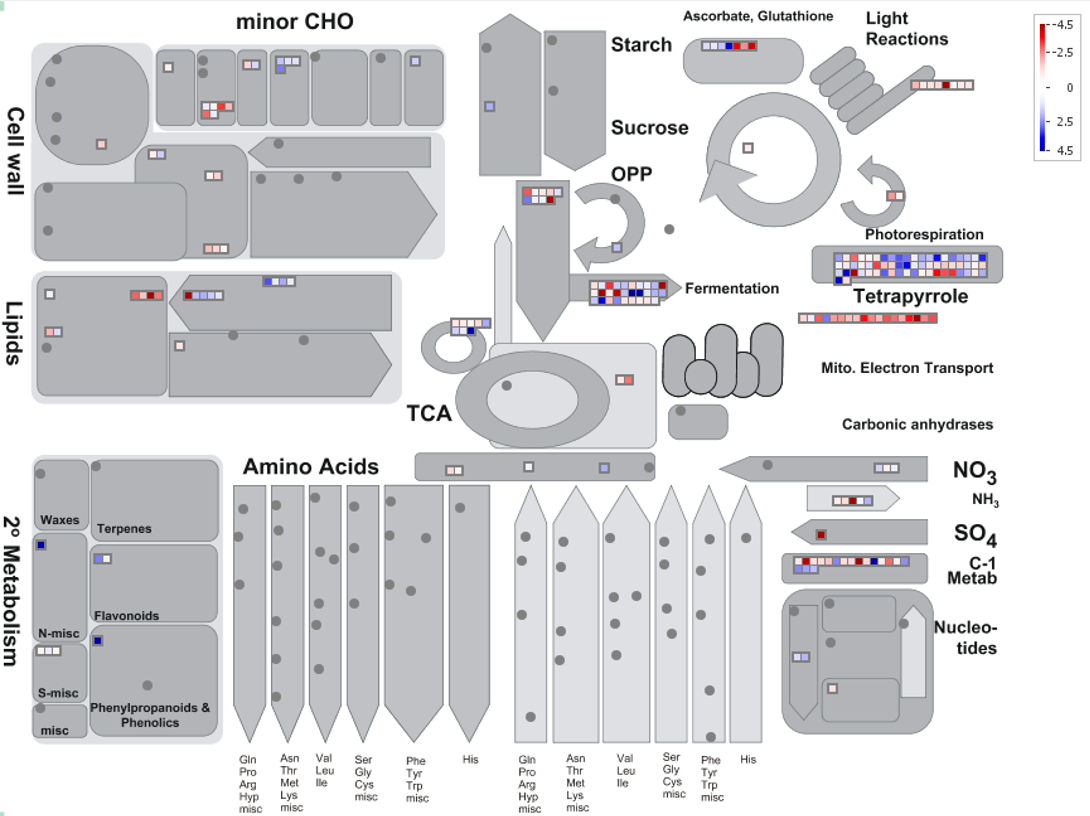
**

**Fig. S3** Metabolism based overview of differentially expressed genes in S. bicolor Water-stressed growth 10 d to 47 d depending on plant tissue age and water-stressed growth. Red: Down-regulated gene transcript. Blue: Up-regulated gene transcripts

**
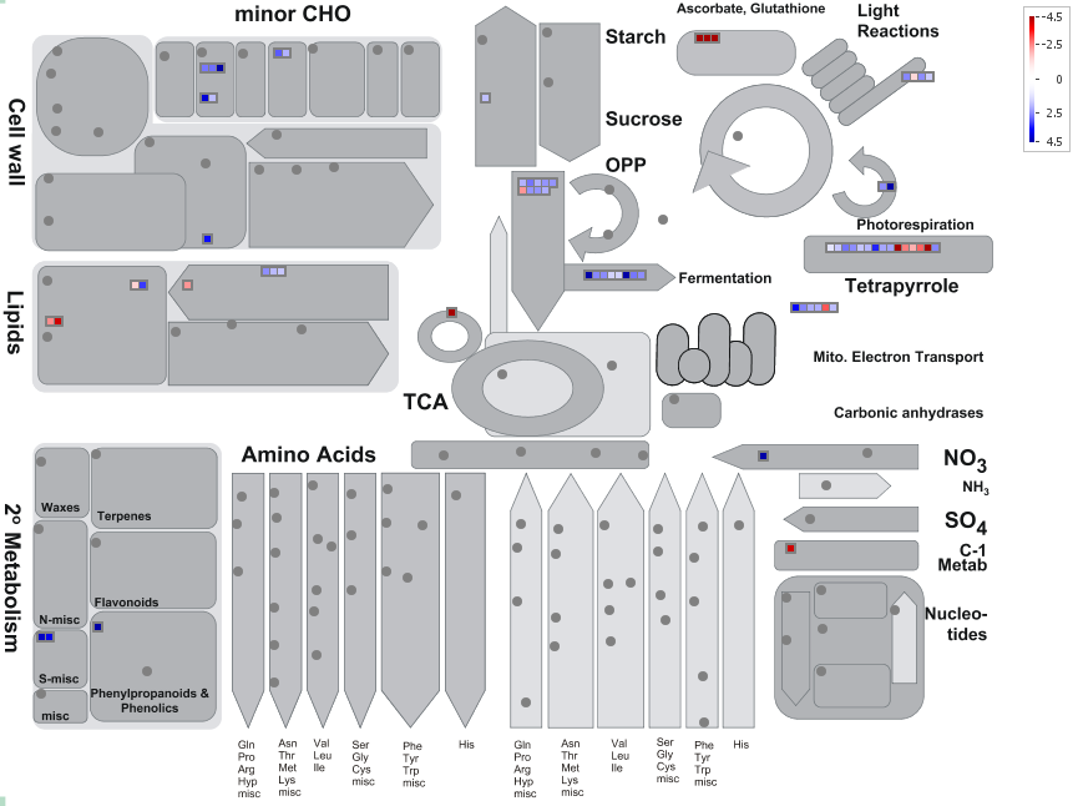
**

**Fig. S4** Metabolism based overview of differentially expressed genes in S. bicolor Control 10 d to water-stressed growth 10 d depending on plant tissue age and water-stressed growth. Red: Down-regulated gene transcript. Blue: Up-regulated gene transcripts

**
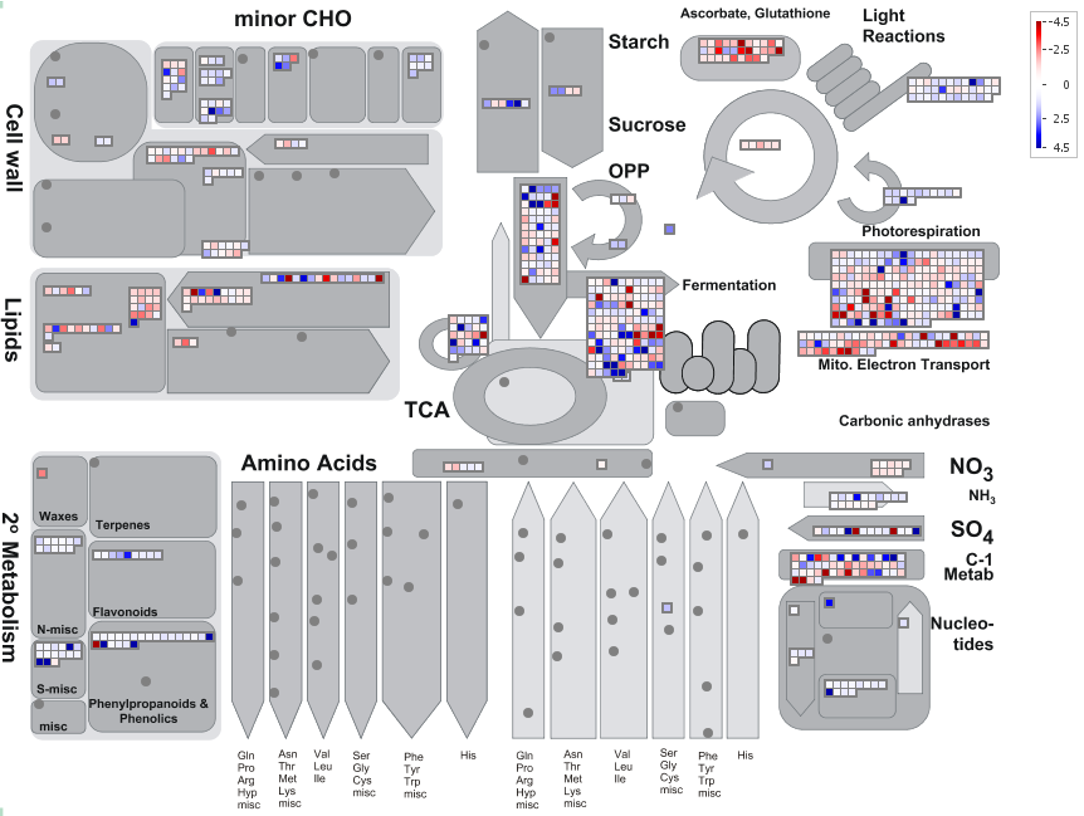
**

**Fig. S5** Metabolism based overview of differentially expressed genes in S. bicolor Control 47 d to water-stressed growth d 47 depending on plant tissue age and water-stressed growth. Red: Down-regulated gene transcript. Blue: Up-regulated gene transcripts

**
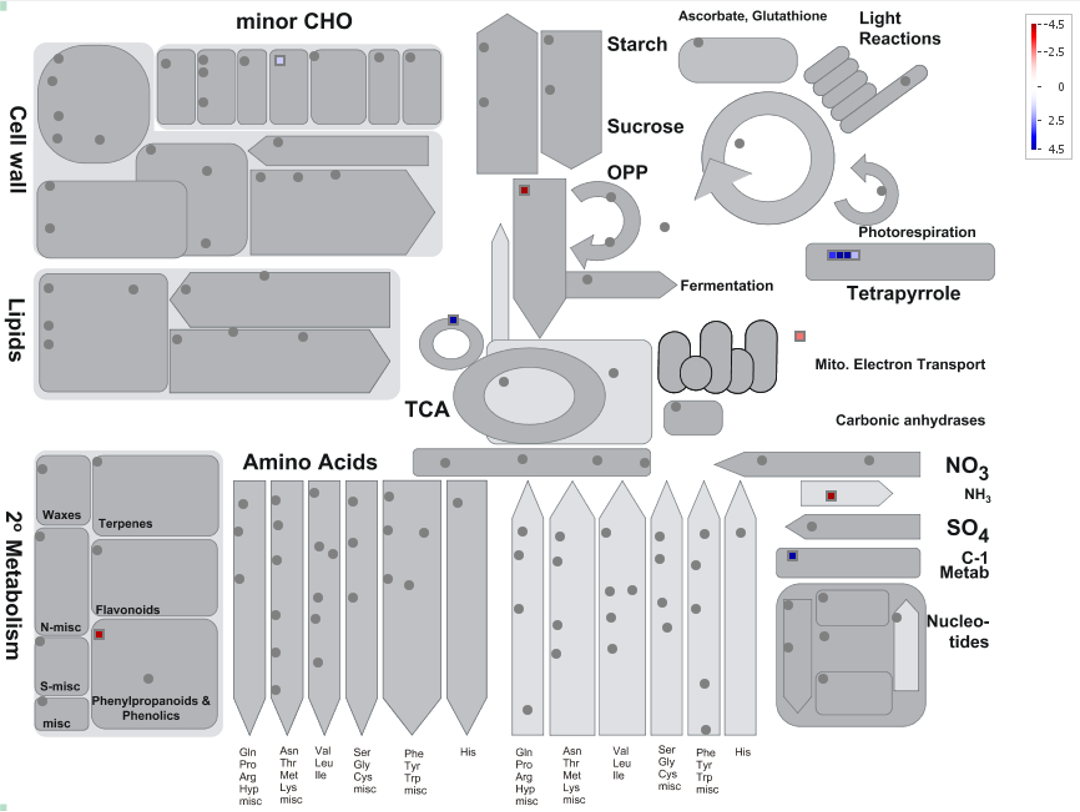
**

**Fig. S6** Metabolism based overview of differentially expressed genes in S. macrospermum Control 10 d to 18 d depending on plant age and imposed water-stressed growth. Red: Down-regulated gene transcript. Blue: Up-regulated gene transcript

**
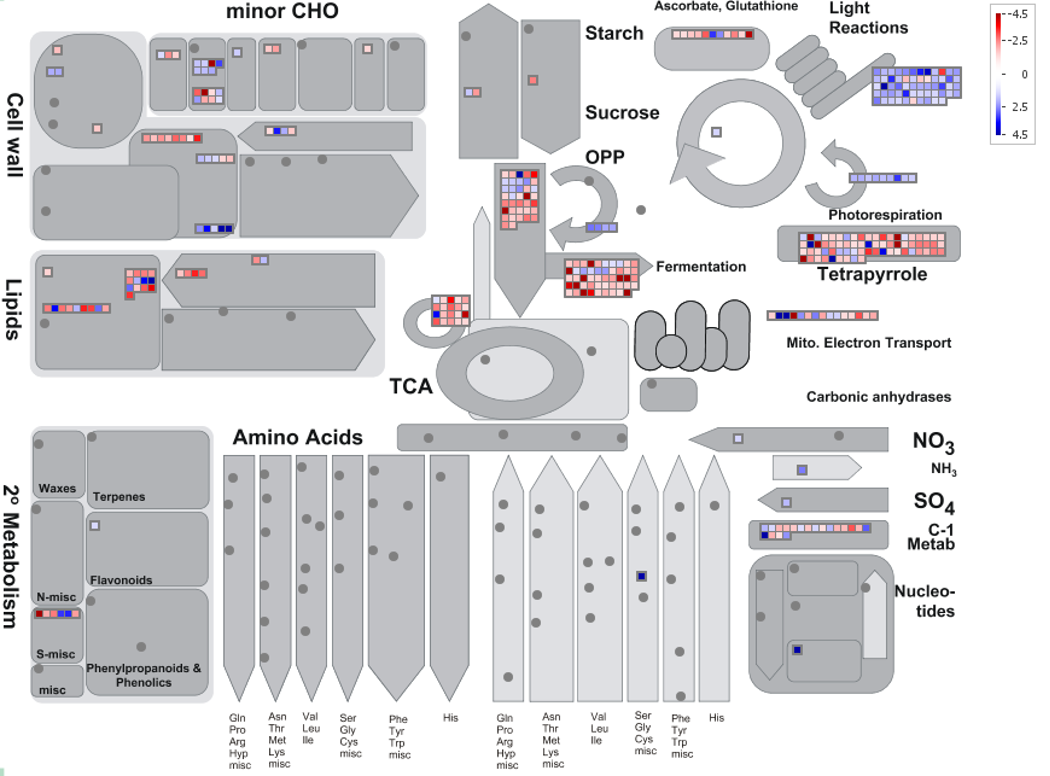
**

**Fig. S7** Metabolism based overview of differentially expressed genes in S. macrospermum Water-stressed growth 10 d to 18 d depending on plant age and imposed water-stressed growth. Red: Down-regulated gene transcript. Blue: Up-regulated gene transcript

**
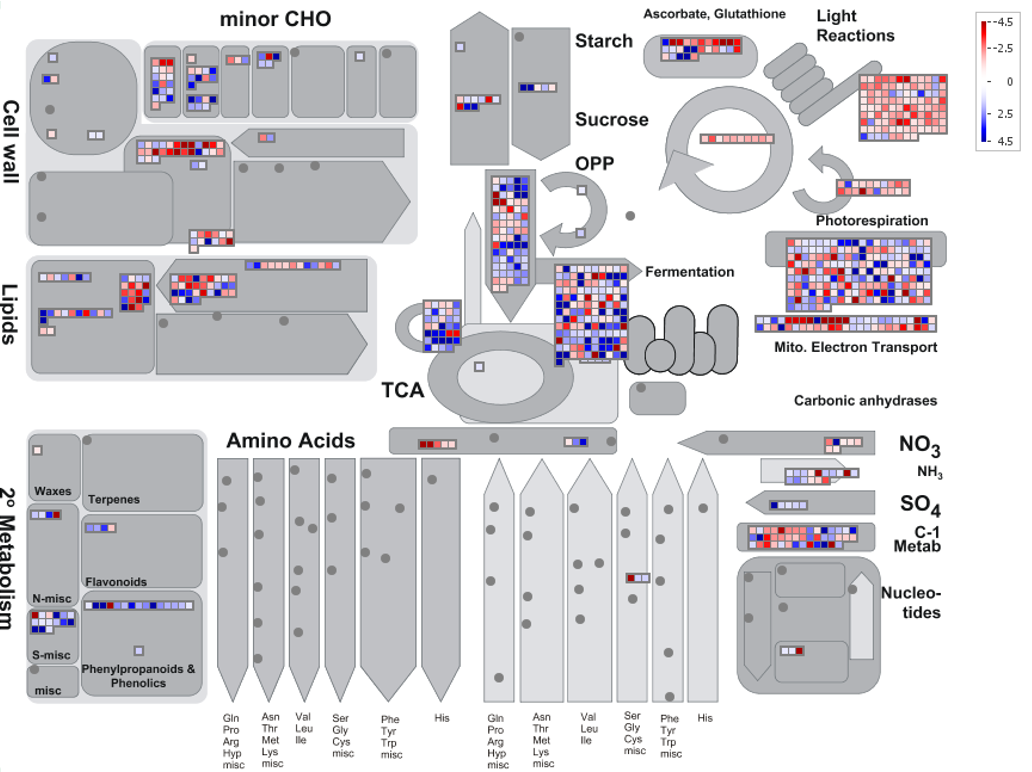
**

**Fig. S8** Metabolism based overview of differentially expressed genes in S. macrospermum Control 10 d to water-stressed growth 10 d depending on plant age and imposed water-stressed growth. Red: Down-regulated gene transcript. Blue: Up-regulated gene transcript


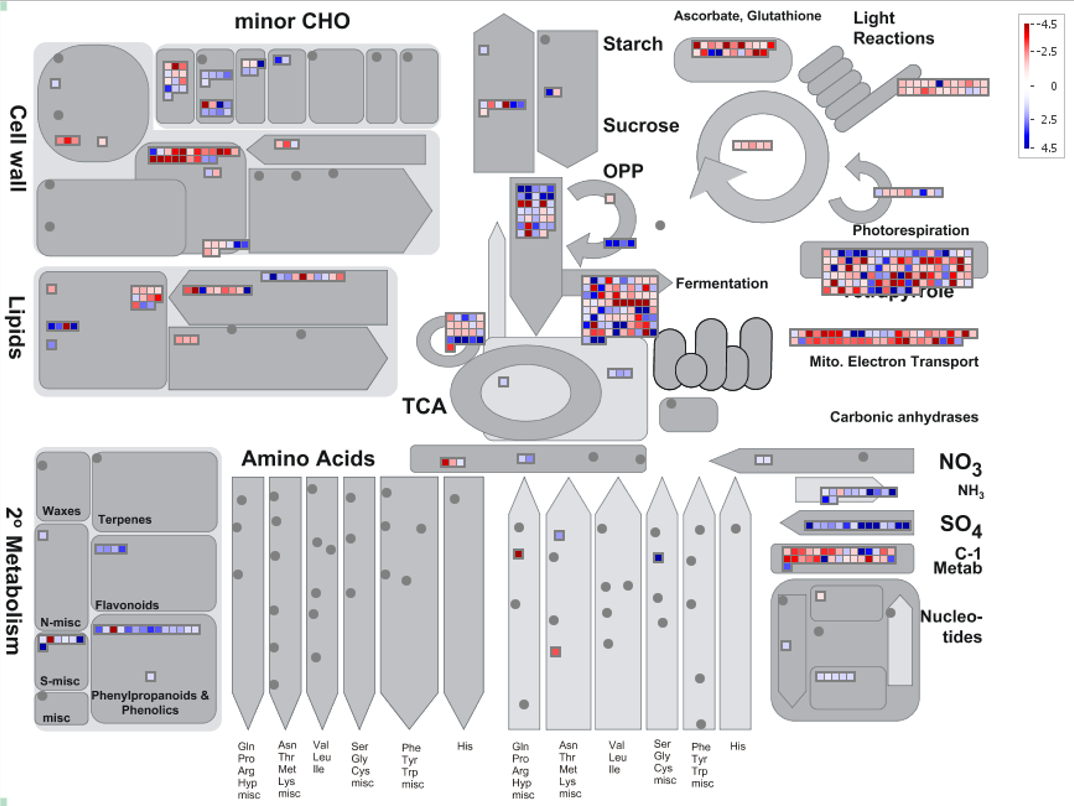


**Fig. S9** Metabolism based overview of differentially expressed genes in S. macrospermum Control 18 d to water-stressed growth 18 d depending on plant age and imposed water-stressed growth. Red: Down-regulated gene transcript. Blue: Up-regulated gene transcript
